# Synthesis and Antiproliferative Evaluation of 3-Chloroazetidin-2-ones with Antimitotic Activity: Heterocyclic Bridged Analogues of Combretastatin A-4

**DOI:** 10.3390/ph14111119

**Published:** 2021-10-31

**Authors:** Azizah M. Malebari, Shu Wang, Thomas F. Greene, Niamh M. O’Boyle, Darren Fayne, Mohemmed Faraz Khan, Seema M. Nathwani, Brendan Twamley, Thomas McCabe, Daniela M. Zisterer, Mary J. Meegan

**Affiliations:** 1 Department of Pharmaceutical Chemistry, College of Pharmacy, King Abdulaziz University, Jeddah 21589, Saudi Arabia; amelibary@kau.edu.sa; 2 School of Pharmacy and Pharmaceutical Sciences, Trinity College Dublin, Trinity Biomedical Sciences Institute, 152-160 Pearse Street, Dublin 2, DO2R590 Dublin, Ireland; wangsh@tcd.ie (S.W.); tgreene@tcd.ie (T.F.G.); niamh.oboyle@tcd.ie (N.M.O.); 3 Molecular Design Group, School of Biochemistry and Immunology, Trinity College Dublin, Trinity Biomedical Sciences Institute, 152-160 Pearse Street, Dublin 2, DO2R590 Dublin, Ireland; fayned@tcd.ie (D.F.); mfkhan@tcd.ie (M.F.K.); 4 School of Biochemistry and Immunology, Trinity College Dublin, Trinity Biomedical Sciences Institute, 152-160 Pearse Street, Dublin 2, DO2R590 Dublin, Ireland; seema.nathwani@outlook.com (S.M.N.); dzistrer@tcd.ie (D.M.Z.); 5 School of Chemistry, Trinity College Dublin, Dublin 2, DO2R590 Dublin, Ireland; twamleyb@tcd.ie (B.T.); tmccabe@tcd.ie (T.M.)

**Keywords:** β-lactam, 3-chloroazetidin-2-ones, antimitotic, antiproliferative activity, breast cancer, tubulin polymerisation, colchicine-binding site, combretastatin A-4

## Abstract

Antimitotic drugs that target tubulin are among the most widely used chemotherapeutic agents; however, the development of multidrug resistance has limited their clinical activity. We report the synthesis and biological properties of a series of novel 3-chloro-β-lactams and 3,3-dichloro-β-lactams (2-azetidinones) that are structurally related to the tubulin polymerisation inhibitor and vascular targeting agent, Combretastatin A-4. These compounds were evaluated as potential tubulin polymerisation inhibitors and for their antiproliferative effects in breast cancer cells. A number of the compounds showed potent activity in MCF-7 breast cancer cells, e.g., compound **10n** (3-chloro-4-(3-hydroxy-4-methoxy-phenyl)-1-(3,4,5-trimethoxyphenyl)azetidin-2-one) and compound **11n** (3,3-dichloro-4-(3-hydroxy-4-methoxyphenyl)-1-(3,4,5-trimethoxyphenyl)-azetidin-2-one), with IC_50_ values of 17 and 31 nM, respectively, and displayed comparable cellular effects to those of Combretastatin A-4. Compound **10n** demonstrated minimal cytotoxicity against non-tumorigenic HEK-293T cells and inhibited the in vitro polymerisation of tubulin with significant G_2_/M phase cell cycle arrest. Immunofluorescence staining of MCF-7 cells confirmed that β-lactam **10n** caused a mitotic catastrophe by targeting tubulin. In addition, compound **10n** promoted apoptosis by regulating the expression of pro-apoptotic protein BAX and anti-apoptotic proteins Bcl-2 and Mcl-1. Molecular docking was used to explore the potential molecular interactions between novel 3-chloro-β-lactams and the amino acid residues of the colchicine binding active site cavity of β-tubulin. Collectively, these results suggest that 3-chloro-2-azetidinones, such as compound **10n**, could be promising lead compounds for further clinical anti-cancer drug development.

## 1. Introduction

Microtubules play an essential role in many cellular functions, such as cell division and mitosis, and are investigated as attractive drug targets in anti-cancer chemotherapy. Many structurally diverse compounds that interfere with microtubule dynamics and spindle formation have been identified. Microtubule-targeting agents (MTAs) have an important role as cancer chemotherapy drugs, e.g., taxol, which stabilises microtubules, and the vinca alkaloids vincristine, vinblastine and vinorelbine, which inhibit the formation of microtubules in the mitotic spindle [1]. These drugs arrest cells in the G_2_/M phase of the cell cycle and effectively target and disrupt mitosis. However, the clinical application of MTAs is restricted by their severe adverse effects [2]. Recent developments in antibody–drug conjugates (ADCs) have resulted in the introduction of effective MTAs for therapeutic use, e.g., ado-trastuzumab emtansine (T-DM1, Kadcyla^®^), comprised of the humanised anti-HER2 IgG1 trastuzumab linked to the anti-mitotic agent, mertansine [3].

**Figure 1:**
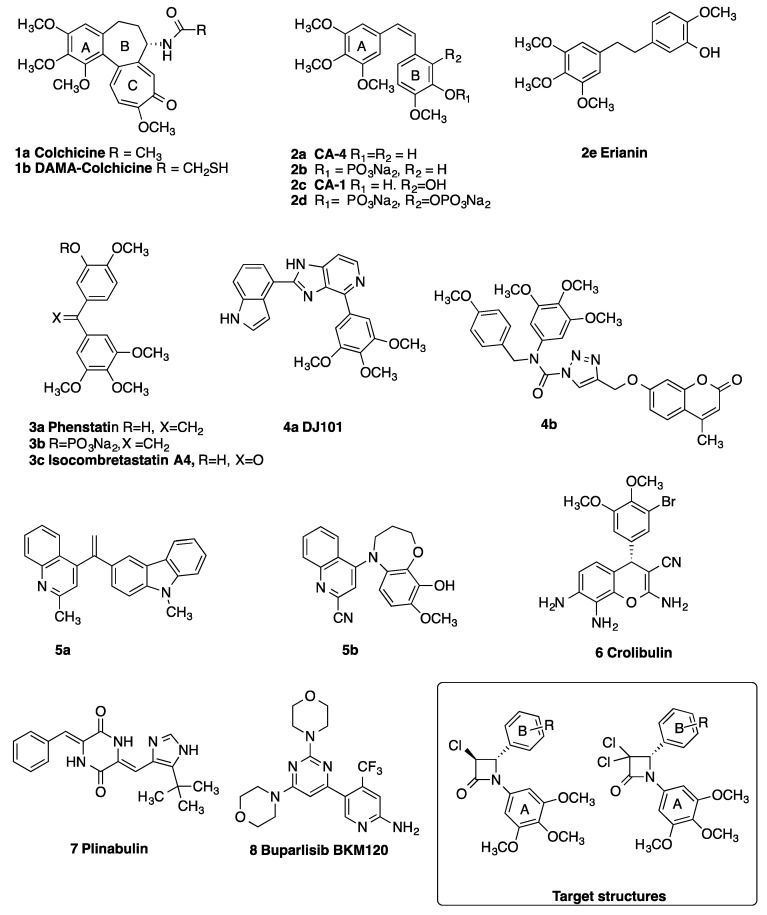
Colchicine (**1a**), DAMA-Colchicine **1b**, Combretastatins **2a**–**2d**, erianin **2e**, phenstatins **3a**, **3b**, isocombretastatin **3c**, colchicine binding site inhibitors **4a (DJ101)**, **4b**, quinolines **5a**, **5b**, Crolibulin **6**, Plinabulin **7**, Buparlisib BKM120 **8** and target β-lactam structures.

MTAs that interact with the following primary tubulin binding sites have been identified: the vinca alkaloid, laulimalide, paclitaxel, epothilone, maytansine, rhizoxin, pironetin, PM060184 [4], colchicine **1a** and other sites (Figure 1) [5]. Ravelli reported the first structural description of DAMA-colchicine **1b** binding in tubulin in 2004 [6]. To date, the structures of many diverse ligands complexed at the colchicine binding site of tubulin have been characterised using X-ray crystallography. Colchicine is not in clinical use as a drug in cancer treatment due to its narrow therapeutic index [7]. Many structural modifications of colchicine are reported in vitro with the objective of lowering colchicine toxicity [8,9], while the various colchicine binding site ligands, such as combretastatin A-4 **2a**, are an extensively investigated group of MTAs, with several currently in clinical trials. In addition, molecules such as CA-4 that bind to the colchicine site have been intensively investigated as vascular-targeting agents (VTAs) [10]. 

The stilbene combretastatin A-4 (CA-4) **2a** isolated in 1989 by Pettit from the bark of the South African Bush tree *Combretum caffrum* [11] has provided a scaffold structure for the extensive investigation of structure-activity relationships in the stilbene series. CA-4P (fosbretabulin **2b**, Figure 1), a water-soluble phosphate prodrug of CA-4 is in phase II/III clinical trials, either alone or in combination with chemotherapeutic agents such as cisplatin [12,13]. Potent anti-vascular properties have also been demonstrated for CA-1(**2c**) and the related phosphate prodrug (**2d**), OXi4503 (Figure 1) [10,14]. The bibenzyl compound erianin **2e** is a novel apoptosis-inducing anti-angiogenic agent [15]. The synthetic benzophenone phenstatin **3a** and phosphate prodrug **3b** show potent cytotoxic activity in cancer cells together with microtubule-destabilising activity [16], while the *iso*Combretastatin **3c**, a stable non-natural isomer of CA-4, shows equivalent anti-cancer properties to CA-4 [17].

To prevent the *cis-trans* isomerisation associated with the storage, metabolism and administration of CA-4 [18,19], bridging heterocyclic analogues of CA-4 have been developed with the objective of restricting the *cis* configuration and optimising the solubility and bioactivity. Examples of diverse carbocyclic and heterocyclic ring systems have been reported, which replace the alkene of CA-4, constrain the aryl rings A and B in a *cis* configuration and demonstrate useful microtubule targeting and anti-cancer activity [20,21]. Many diverse heterocyclic compounds related in structure to colchicine have been identified as MTAs and some examples are illustrated in Figure 1. DJ101 **4a**, a novel metabolically stable indolylimidazopyridine containing the 3,4,5-trimethoxyphenyl substituent characteristic of colchicine and CA-4, depolymerises microtubules and is effective against a broad panel of metastatic melanomas and is effective in overcoming P-gp-mediated multidrug resistance (MDR) [22]. 

Trimethoxyphenyl-1,2,3-triazole hybrids, such as **4b**, containing the coumarin fragment, inhibit human gastric MGC803 cancer cell growth, induce G_2_/M phase arrest by down-regulating the expression of CDK1, promote apoptosis by regulating Death Receptor 5 (DR5) and the Bcl-2 family of proteins and inhibit tubulin polymerisation by interacting with the colchicine site [23]. The quinaldinyl-*iso*-carbazolyl compound **5a** is more active than CA-4 **1a** and *iso*CA-4 **3c** against A549, lung adenocarcinoma epithelial cells [24]. The novel benzoxazepine **5b**, related in structure to *iso*CA-4 **3c**, displays significant cytotoxicity against HCT116 and K562 cancer cell lines, and inhibits tubulin polymerisation and induces G_2_/M arrest (Figure 1) [25]. The 4-aryl-4*H*-chromene Crolibulin **6** binds to the colchicine-binding site on *beta*-tubulin and has undergone a Phase I/II clinical trial for anaplastic thyroid cancer [26]. The crystal structure of tubulin complexed with crolibulin **6** has been determined [27] and revealed that the chromene moiety of crolibulin adopts a similar position as the B and C rings of colchicine, while not containing the characteristic 3,4,5-trimethoxyphenyl ring. Although it is more deeply buried in β-tubulin, it is closer to some hydrophobic amino acids than colchicine. 

It is interesting to observe that the orally active diketopiperazine plinabulin **7** selectively targets and binds to the colchicine-binding site of tubulin [28], blocks tumour growth [29] and provides early protection against severe neutropenia induced by chemotherapy in patients with advanced NSCLC (Figure 1) [30]. BKM120 (Buparlisib) **8** is one of the most advanced phosphoinositide 3-kinase (PI3K) inhibitors for the treatment of cancer, but it has been shown to also interfere with microtubule polymerisation as an off-target effect [31]. Although considerable progress has been achieved in the discovery of targeted cancer therapies, both innate and acquired mechanisms of resistance are commonly observed for many successful cancer drugs [32]. 

We have reported the synthesis, antiproliferative and tubulin-binding effects of a series of 2-azetidinones (β-lactams), containing the structural features of CA-4, while retaining the necessary *cis* configuration of Rings A and B [33,34]. β-Lactam compounds containing aryl and heterocycles such as thiophene located at C-3 were found to be particularly effective [35,36,37]. In addition, both the anti-angiogenic and anti-migratory effects observed in MDA-MB-231 breast adenocarcinoma cells suggest a potential anti-metastatic role for these compounds [38]. We have identified the *β*-lactam heterocycle as a potential scaffold for the development of new anti-tumour agents and wished to establish the structural requirements for substituents at C-3. 

The anti-cancer activity of structurally diverse monocyclic β-lactam compounds has been previously reported [39,40,41,42,43,44,45,46]. Chiral azetidin-2-ones were designed as non-isomerisable CA-4 analogues disrupting tubulin polymerisation, inducing cellular apoptosis and suppressing angiogenesis [47,48,49]. 3-Hydroxy-1,4-diaryl-2-azetidinones induce apoptosis, with the activation of AMP-activated protein kinase (AMPK) in colon cancer [50], while 3-methoxy-β-lactams show a significant decrease in AKT kinase activity, a cell survival pathway identified in breast cancer [51]. Piperazine modified azetidinone derivatives suppress proliferation and migration in human cervical cancer HeLa cells [52]. Interestingly, 1,3-disubstituted cyclobutane-containing analogues of combretastatin A4 were evaluated for their cytotoxic properties in human cancer cell lines HepG2 (hepatocarcinoma) and SK-N- DZ (neuroblastoma) [53]. Although β-lactam antibiotics such as penicillins and cephalosporins are best known for their antibacterial activities [54], antimicrobial [55,56], antifungal [57,58] and anti-filarial [59] activities have also been demonstrated for monocyclic β-lactams. 

We now report a series of novel 3-chloro-2-azetidinone and 3,3-dichloro-2-azetidinone compounds with an interesting profile, particularly in triple negative breast cancer, which could be considered for potential development as tubulin destabilising agents in preclinical studies of breast cancer (see Figure 1, target structures). A library of 1,4-diarylazetidin-2-ones that contain halogen substituents chloro, dichloro or bromo at C-3 was prepared for evaluation. The β-lactam ring forms a rigid scaffold for the hydrophobic CA-4 aryl rings A and B required for interaction with the colchicine binding site of tubulin. The effect of these C-3 halogen substituents on the biological activity of these compounds when the *cis* configuration (Rings A and B) is constrained into the 4-membered azetidin-2-one ring structure was investigated. The synthesis of the phosphate ester prodrugs of the most potent 3-chloro-1,4-diarylazetidin-2-one was also examined, to increase the potential bioavailability of the compound. The introduction of this halogen substituent at C-3 also allowed us to examine potential structure-activity relationships for the series, and to rationalise the effect of the introduction of the C-3 chlorine on the interaction with the colchicine binding site. We have now investigated a new series of novel 3-halo-2-azetidinone compounds with an improved biochemical profile, e.g., in triple negative breast cancer for potential development in the treatment of breast cancer as tubulin destabilising agents. These novel heterocyclic structures were further investigated for their effects on cell viability, cell cycle and tubulin polymerisation in MCF-7 breast cancer cells. 

## 2. Results and Discussion 

### 2.1. Chemistry

**Scheme 1:**
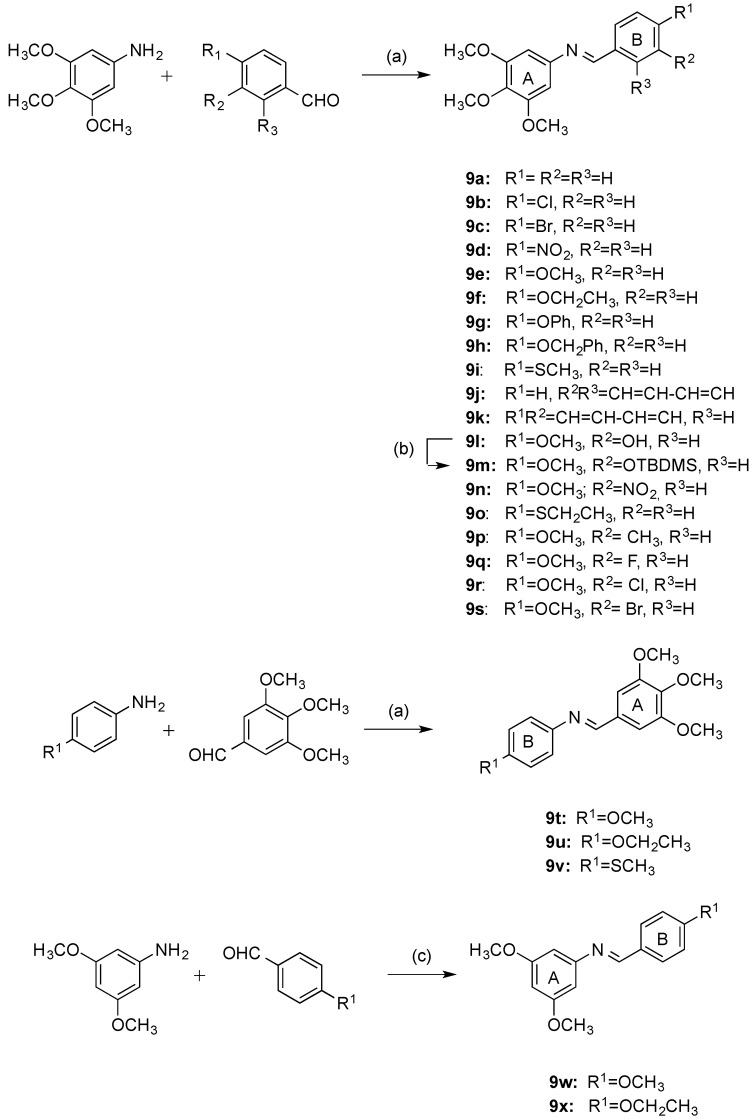
Synthesis of imines **9a**–**x**. Reagents and conditions: (**a**) EtOH, conc. H_2_SO_4_, reflux, 4 h (70–92%); (**b**) TBDMSCl, DBU, CH_2_Cl_2_, 20 °C (78%); (**c**) H_2_O, 30 min, 20 °C (85–97%).

**Figure 2:**
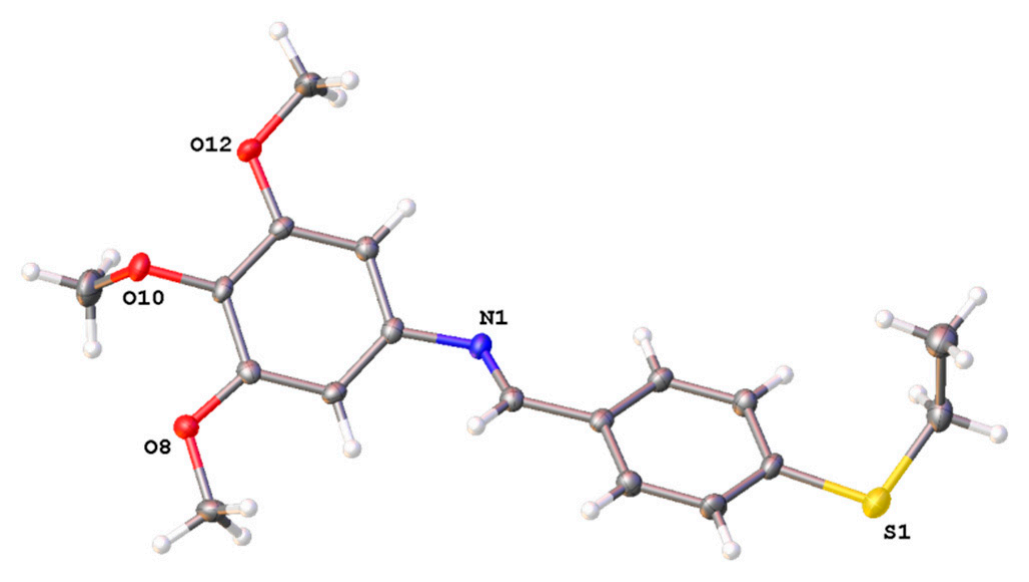
ORTEP representation of the X-ray crystal structure of compound **9o** with the thermal ellipsoids set at 50% probability.

The azetidinones required for the present study were prepared using the Staudinger ketene-imine cycloaddition reaction. The imines **9a**–**9s** were obtained using a condensation reaction of the 3,4,5-trimethoxyaniline with the appropriately substituted benzaldehyde, Scheme 1. The silyl ether compound **9m** was initially obtained by a reaction of **9l** with *tert*butyldimethylsilyl chloride (TBDMSCl) in a 78% yield; however, a cleaner silyl ether product **9m** was obtained if the protection was carried out first on 3-hydroxy-4-methoxybenzaldehyde (94%). 3-((*tert*-Butyldimethylsilyl)oxy)-4-methoxybenzaldehyde was then reacted with 3,4,5-trimethoxyaniline to afford the protected imine **9m** in a 76% yield. The silyl ether was removed under mild conditions at a later stage in the synthetic route without the degradation of the β-lactam ring. The compounds **9a**–**9s** contain a 3,4,5-trimethoxyaryl ring at N-1, present in ring A of CA-4, together with a second aryl ring positioned at the C-4 position of the β-lactam ring containing various substituents (Ring B). Alternative approaches were investigated for the preparation of the 3,5-dimethoxyphenyl substituted imines **9w** and **9x** to optimise the yield and purity of the product, e.g., use of isopropanol as a solvent, and sulfuric acid, benzoic acid or boric acid as a catalyst. However, optimum yields for compounds **9w** and **9x** (97 and 85%, respectively) were obtained when these reactions were carried out in aqueous conditions and at an ambient temperature for less than one hour, Scheme 1 [60]. Imines **9t**–**9v** were prepared using a condensation reaction of the 3,4,5-trimethoxybenzaldehyde with the appropriately substituted anilines and allowed for the positioning of the 3,4,5-trimethoxyaryl Ring A at C-4 of the β-lactam. Imines **9w** and **9x** are designed to replace the 3,4,5-trimethoxyaryl Ring A with the alternative 3,5-dimethoxyphenyl substitution. This substitution pattern is characteristic of stilbenes such as resveratrol (3,4′,5-trihydroxystilbene) identified as having therapeutic and chemopreventive roles in colorectal and skin cancers [61,62], and is also present in the related stilbenes pinosylvin, isorhapontigenin, pterostilbene [63,64], which also elicit anti-cancer properties. The structure of the imine **9o** was confirmed using X-ray crystallography (Figure 2 and Table 1) showing the *E* configuration of the imine with an N1-C2 bond length of 1.280(2) Å. The torsion angle between the N=C-C-phenyl ring was determined as −175.33°, while the torsion angel for the C=N-C- phenyl ring was −34.4°. The packing structure assumed by the products was centrosymmetric and monoclinic.

**Scheme 2:**
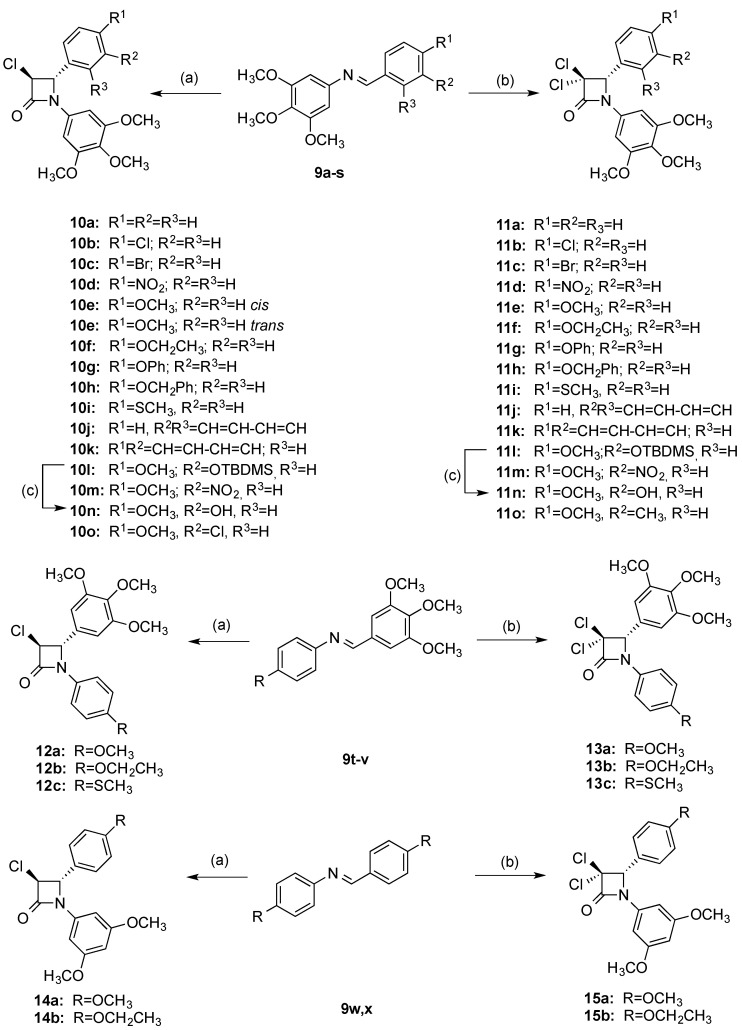
Synthesis of β-lactams **10a**–**o**, **11a**–**o**, **12a**–**c**, **13a**–**c**, **14a**–**b** and **15a**–**b**. Reagents and conditions: (**a**) (i) ClCH_2_COCl, triethylamine, CH_2_Cl_2_, reflux, 5 h (**10a**–**o**, 3–57%; **12a**–**c**, 45–59%; **14a**–**b**, 8–20%) or (ii) ClCH_2_COCl, triphosgene, CH_2_Cl_2_, reflux, 6 h (**10n**, 34%); (**b**) Cl_2_CHCOCl, triethylamine, CH_2_Cl_2_, reflux, 5 h (**11a**–**o**, 17–63%; **13a**–**c**, 18–47%; **15a**–**b**, 7–13%); (**c**) TBAF, dry THF, 0 °C, 30 min (28–34%).

The Staudinger reaction ([2+2] ketene–imine cycloaddition reaction) is a versatile method for the synthesis of 2-azetidinones. Ketenes are usually formed in situ by a reaction of acyl halides with tertiary amines. However, 2-azetidinones are also directly accessible from imines and carboxylic acids via mixed anhydrides [65], using activating agents such as methoxymethylene-*N*,*N*-dimethyliminium salt, [66], the Vilsmeier reagent, the Mukaiyama reagent and triphosgene [67]. 3-Chloro-3-thioaryl-β-lactams, obtained by the chlorination of 3-thioaryl-β-lactams with sulfuryl chloride, are reported as suitable substrates for Lewis acid catalysed nucleophilic substitution reactions [68]. In the present work, the Staudinger reaction of the imines **9a**–**9k** and **9m**–**9s** with chloroacetyl chloride in the presence of triethylamine (Scheme 2) afforded the β-lactam products **10a**–**m** and **10o** as racemic mixtures in yields of 3–57%. Compound **10l** was also obtained in a reaction of the imine **9l** with chloroacetic acid with triphosgene as the acid activating agent. Deprotection of the silyl ether **10l** with TBAF afforded the phenolic product **10n**. Low yields in some of the Staudinger reactions were due to the degradation of the imine commonly observed in these reaction mixtures. 

The additional 3-chloro-β-lactam products **12a**–**12c**, **14a** and **14b** were also obtained in a similar Staudinger reaction of the appropriate imines **9t**–**9v**, **9w** and **9x** with chloroacetyl chloride. The coupling constant for H-3 and H-4 of the azetidinone ring is generally useful in the identification of the stereochemistry of 2-azetidinones, with *J*_3,4_ usually of 4–6 Hz for the *cis* and *J*_3,4_ 1–2 Hz for the *trans* stereoisomers. The structural assignment of 3-monochloro β-lactam **10n** was particularly interesting for the assignments of H-3 and H-4 protons of the β-lactam compound. Two doublet signals were observed in the ^1^H NMR spectrum for H-4 and H-3 at δ 4.61 and 4.89 ppm, respectively (*J*_3,4_ = 1.52 Hz), which confirmed the *trans* isomer assignment) and were more downfield due to the adjacent electron withdrawing chlorine substituent. In the ^13^C NMR spectrum of **10n**, the corresponding C3 and C4 carbons of β-lactam appeared at 62.67 (C_3_) and 65.63 (C_4_) ppm, respectively, with the characteristic signal of carbonyl group of the β-lactam at δ 160.22 ppm. The compounds were isolated exclusively as the *trans* isomer; the only exception was compound **10e**, where both the *trans* and *cis* compounds were obtained (*trans:cis* ratio 1.9:1; *J*_3,4_ = 5.00 Hz *cis*, *J*_3,4_ = 2 Hz *trans*).

**Figure 3:**
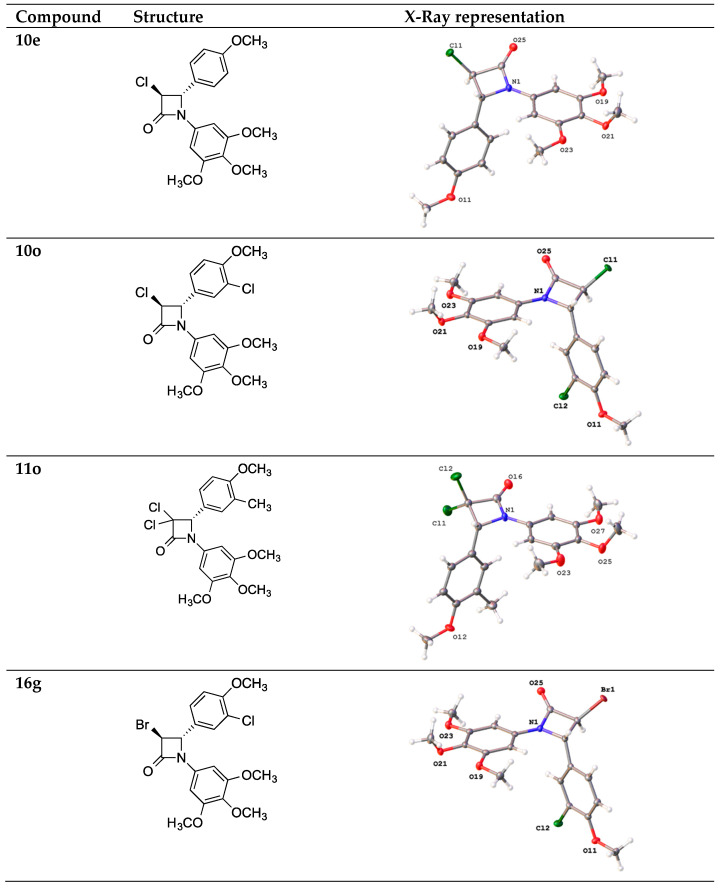
ORTEP representation of the X-ray crystal structure of compounds **10e**, **10o**, **11o** and **16g** with heteroatoms labelled and thermal ellipsoids set at 50% probability. Structure **11o** shows the majority occupied disordered 4-methoxyphenyl moiety (81% occupied). For structures **10o** and **16g**, the 3 position is substituted by both chloride (75%) and bromide (25%); distances restrained, and atomic displacement constrained.

Single crystal X-ray analysis was obtained for compounds **10e** and **10o** (recrystallised from dichloromethane/*n*-hexane), and the crystal structure is shown in Figure 3. The crystal data and structure refinement for the 3-chloro compounds **10e** and **10o** are displayed in Table 1 and Table 2. The *trans* stereochemistry for compounds **10e** and **10o** was confirmed from the X-ray crystal structures. The aryl rings at N-1 (Ring A) and C-4 are in a non-coplanar *cis-like* arrangement, while the phenyl ring at C4 (Ring B) and the 3-chloro substituent are in a *trans* configuration on opposite sides of the β-lactam ring. For compound **10e**, the distance between the centroid of ring A and ring B is ~5.2 Å, while the distance between ring B and the chloro group is ~5.0 Å. For compounds **10e** and **10o**, the torsional angle value ring B/C was calculated as 116.0(1)° and 112.0(3)°, respectively, which is consistent with the small *trans* coupling constant observed in the ^1^H NMR spectrum of 2.00 and 2.20 Hz, respectively, for H3/H4 in these compounds. The β-lactam C=O bond lengths are 1.2122(18) Å and 1.2122(19) Å for compounds **10e** and **10o**, respectively, which is consistent with the data previously reported for the carbonyl bond length of monocyclic β-lactams of 1.217(3) Å [69] and 1.207(2) Å [70]. The torsional angles (Ring A/B) observed for compounds **10e** and **10o** were calculated to be 60.7(2)° and 62.7(2)°, respectively; these values are slightly greater than the corresponding torsional angles for Ring A/B of 55° and 53° reported in DAMA-colchicine and CA-4, respectively [6,71]. The numbers in parentheses refer to the second crystallographically independent molecule in the asymmetric unit.

As an extension to this study, a further series of 3,3-dichloro-β-lactams (**11a**–**11o**, **13a**–**c**, **15a**, **15b**) with similar substituents in rings A and B as that described for the 3-chloro β-lactams was prepared (Scheme 2). The phenolic product **11n** was obtained by treating the silyl ether **11l** with TBAF. The compounds were obtained in moderate yields (27–63%), apart from compounds **15a** and **15b** (with 3,5-dimethoxy substitution pattern in the aryl A ring), which were isolated in low yields, 13 and 7%, respectively. When comparing the 3,3-dichloro-β-lactams with the mono chloro β-lactam compounds, the ^1^H NMR spectrum for the 3,3-dichloro β-lactam compound **11n** is relatively simple; the characteristic signal for 3,3-dichloro β-lactam compounds appeared as a singlet for the H4 proton at δ 5.39 ppm and it is further downfield than in the corresponding **10n** because of the electron withdrawing properties of the two chlorine atoms. In the ^13^C NMR spectrum, the high resonance signals at δ 83.68 and 73.54 ppm were assigned to C3 (with the dichloro substituent) and C4, respectively, while the resonances for C3 and C4 were observed at 62.67 (C_3_) and 65.63 (C_4_) ppm in the 3-monochloro compound **10n**. The X-ray crystal structure and data for the 3,3-dichloro β-lactam compound **11o** are presented in Figure 3 and Table 1 and show that rings A and B (located at N-1 and C-4 of the β-lactam ring) are not coplanar, with a torsional angle of 68.9° (see Table 2). A significant difference in the distances from the centroid of ring B to each of the two C3 chloro substituents (3.7 Å and 5.4 Å) was observed. This may be relevant in rationalising the differences in the antiproliferative activity observed for the monochloro and dichloro-β-lactam compounds. 

**Scheme 3:**
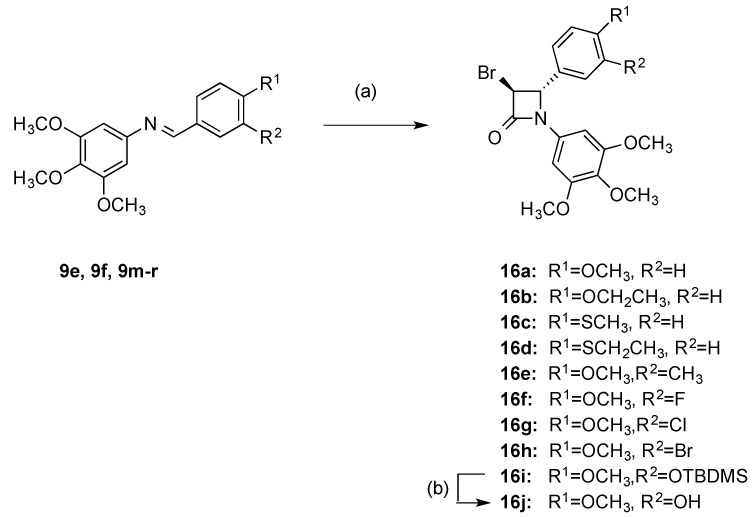
Synthesis of β-lactams **16a-i**. Reagents and conditions: (**a**) BrCH_2_COCl, toluene, reflux, 5 h (5–31%); (**b**) TBAF, dry THF, 0 °C, 30 min (32%).

As the 3-chloro-β-lactams exhibited an excellent antiproliferative profile, a further series of related 3-bromo β-lactams was investigated by using the Staudinger procedure with bromoacetyl chloride (Scheme 3). The preparation of 3-bromo β-lactams using bromoacetyl bromide [72], bromination of 3-azetidinone [48] and a ring expansion of aziridines with triphenylphosphine/NBS or triphenylphosphine dibromide has been reported [73]. The 3-bromo β-lactams (**16a**–**16i**) were initially obtained as a mixture with corresponding 3-chloro-β-lactams in a ratio of 1:2 in most cases in yields of 5–31%, due to the halogen exchange with the chlorinated solvent (dichloromethane), following purification by either recrystallisation or from the gradient column chromatography. The presence of the *trans* isomer of 3-bromo-β-lactam **16a** was confirmed from the ^1^H NMR spectrum that shows H-3 at δ 5.05 and H-4 at δ 4.65 (*J* 1.96 Hz) (in comparison, H-3 and H-4 of the 3-chloro β-lactam **10e** were observed at δ 4.95 and δ 4.63, *J* 2.00 Hz). The phenolic product **16j** was obtained by treating the silyl ether **19i** with TBAF. The asymmetric synthesis of **16j** (*3S,4S*) was previously reported [48]. The X-ray crystal structure and data for the 3-bromo-β-lactam compound **16g** (Figure 3, Table 1) again demonstrates that rings A and B (located at N-1 and C-4 of the β-lactam ring) are not coplanar (see Table 2) with Ring A/B having a torsional angle of 62.7(2)°, and Ring B/C(β-lactam) having a torsional angle value of 110.3°.

**Scheme 4:**
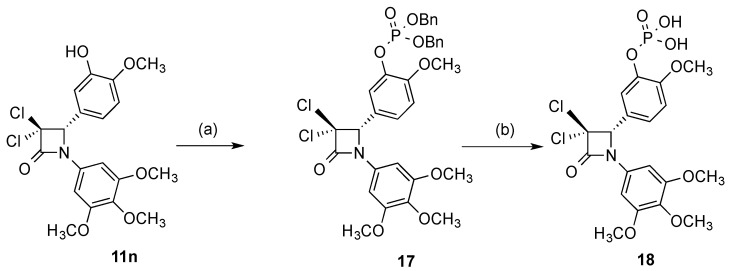
Synthesis of phosphate prodrug **18**. Reagents and conditions: (**a**) dibenzyl phosphate, DIPEA, DMAP, CCl_4_, CH_3_CN, -10–20 °C, 3h. (66%); (**b**) H_2_/Pd/C, ethanol-ethyl acetate, 1:1, 3 h, 20 °C (75%).

To improve the solubility and bioavailability of the compounds, the phenol **11n** was selected for phosphate ester prodrug preparation. The esterification of the phenol **11n** with dibenzyl phosphite using diisopropylethylamine and dimethylaminopyridine afforded dibenzyl phosphate β-lactam ester **17** (Scheme 4). The subsequent hydrogenation of **17** with a palladium/carbon catalyst removed the dibenzyl protecting groups to afford the phosphate ester **18** in a 75% yield, while the β-lactam ring remained intact.

HPLC stability studies at three different pH systems were performed on a representative compound **16a** to determine the stability at acidic pH 4, pH 7.4 and basic pH 9 (acid pH found in the stomach, basic found in the intestine and pH 7.4 in the plasma). The compound was stable at these buffered pH systems with a half-life of 18 h (pH 4), 20 h (pH 9) and 22 h (pH 7.4).

### 2.2. Biochemical Results 

#### 2.2.1. Activity of β-Lactam Compounds in MCF-7 Human Breast Cancer Cell Line

The antiproliferative potential of the β-lactams **10a**–**o**, **11a**–**o**, **12a**–**c**, **13a**–**c**, **18**, **14a**, **14b**, **15a**, **15b** and **16a**–**h** was initially evaluated in the CA-4 sensitive oestrogen receptor positive MCF-7 human breast cancer cell line. CA-4 was used as the control compound in the assay (Table 3), together with β-lactam compounds that we had previously reported [34,74]. The IC_50_ value obtained for CA-4 (0.0039 μM for MCF-7) is in good agreement with reported values [75]. The introduction of the halogen substituent at C-3 was examined in an effort to investigate the effect on the activity of this substituent, and subsequently optimising the cytotoxic effects against MCF-7 human breast cancer cells. The most potent analogues in MCF-7 cells were further screened in the MDA-MB-231, Hs578T and Hs578Ts(i)8 triple negative breast cancer cell lines, multiple myeloma (U266), acute myeloid leukaemia (HL60) and colon cancer (HT-29 and SW480) cell lines using the AlamarBlue assay. Compounds were initially assessed for antiproliferative activity in MCF-7 cells to determine the structure–activity relationship for these halogenated compounds and to identify the most potent compounds to progress for further investigation. 

In the series of compounds with 3-chloro substituent **10a**–**o**, a number of varied substituents were introduced at C-4 of Ring B, while retaining the 3,4,5-trimethoxy substitution for Ring A usually present in many colchicine binding-site type ligands [19]. The introduction of nitro (**10d**), chloro (**10b**) and bromo (**10c**) at C-4 of Ring B resulted in increased activity when compared with the unsubstituted **10a**, with the 4-methoxy **10e** and 4-thiomethyl **10i** showing excellent potency (IC_50_ 34 and 73 nM, respectively [74]). The *cis* isomer of **10e** demonstrated a nine-fold reduction in activity (IC_50_ 0.317 μM) when compared with the *trans* isomer (IC_50_ 34 nM). The bulkier 4-phenoxy **10g** and 4-benzyloxy **10h** substituents resulted in significantly reduced activity (IC_50_ 64.07 and 59.91 μM, respectively). Interestingly, the 2-naphthyl **10k** was much more potent (IC_50_ 0.20 μM) than the 1-naphthyl compound **10j** (IC_50_ 14.66 μM), possibly due to the steric interference by the 1-naphthyl at the colchicine binding site, while the 2-naphthyl was more easily accommodated. Compounds **10m** and **10o**, with an additional substituent at the *meta* position of B ring (**10m** nitro and **10o** chloro), retained moderate activity with IC_50_ values of 3.088 and 0.433 μM, respectively. 

The most potent compound in this series was identified as **10n**, with the characteristic CA-4 3-hydroxy-4-methoxyphenyl Ring B substitution (IC_50_ = 0.017 μM), which compares favourably with CA-4 (IC_50_ 0.004 μM). Analysis of the results from the 3,3-dichloro compound series **11a**–**n** showed that the compounds displayed a similar SAR profile to the 3-chloro compounds but with reduced potency, with compounds **11e**, **11f**, **11i**, **11k** and **11m** displaying IC_50_ values in the range 0.119–0.353 μM. The most potent compound in this series was identified as **11n**, again with the characteristic CA-4 3-hydroxy-4-methoxyphenyl Ring B substitution (IC_50_ = 0.031 μM). Interestingly, the prodrug **18** (the phosphate ester of the phenol **11n**) retained potent activity with IC_50_ = 0.077 μM. The introduction of the 3,4,5-trimethoxyaryl ring A at C-4 of the β-lactam in both the 3-chloro and 3,3-dichloro series (compounds **12a**–**c**, **13a**–**c**) resulted in significant decrease in activity, e.g., compound **10e** (IC_50_ = 0.034 μM (*trans*) and 0.317 μM (*cis*)) compared with **12a** (IC_50_ = 14.81 μM). The poor activity of compounds **12a**–**c** and **13a**–**c** where the 3,4,5-trimethoxyphenyl group A is located at C-4 could be due to the bulkiness of Ring A, which is unable to fit correctly in the target binding pocket of tubulin, in agreement with previous reports findings [33].

In compounds **14a**, **14b**, **15a** and **15b**, the 3,5-dimethoxyaryl ring is located at N-1 of β-lactam and replaces the usual 3,4,5-trimethoxyaryl ring A. Compound **14b** with the *para* OEt substituent in Ring B produces a remarkably better antiproliferative effect (IC_50_ = 0.045 μM) than the *para* OMe for both 3-chloro and 3,3-dichloro compounds. **14b** was identified as of particular interest and only slightly less potent than the corresponding 3,4,5-trimethoxy analogue **10e** (IC_50_ = 0.034 μM). The 3,4,5-trimethoxy substituted A Ring of CA-4 plays an important role in inhibiting tubulin polymerisation, confirmed by the crystal structure of CA-4 in tubulin [19]. It is interesting to see that the removal of the 4-methoxy group results in the retention of activity in the 3,5-dimethoxyaryl ring A compound **14b**. The introduction of the 3-bromo substituent to replace the chloro at C-3 of the β-lactam resulted in a significant reduction in the antiproliferative effect of the compounds in the series, e.g., comparing compound **16a** (IC_50_ = 0.579 μM) with the corresponding 3-chloro compound **10e** (IC_50_ = 0.034 μM) resulted in a 17-fold decrease in activity. 

The physicochemical properties and metabolic stability of the panel of compounds synthesised were evaluated to probe into the drug-relevant properties (see Supplementary Information Tables S1 and S2 for Tier 1 profiling screen). The physicochemical properties of the compounds complied with the Lipinski’s rule of five, thus ensuring a good lipophilic–hydrophilic balance and adequate membrane permeability. Most of the compounds followed Lipinski and Veber rules, i.e., molecular weight ranges from 347 to 457, hydrogen bond acceptor range between four and nine, hydrogen bond donor range between zero and two, lipophilicity (AlogP) appeared in the range 2.67–4.76 (apart from the 4-naphthyl compound **11k**, AlogP 5.84) and the number of rotatable bonds in the range 5–8. The calculated TPSA was between 48 and 130 Å^2^, which suggested good intestinal absorption. The pharmacokinetics results indicate that these compounds satisfy the criteria for good drug likeness parameters and good bioavailability. The compounds were free from alerts for Pan Assay Interfering substances (PAINS) [76] and are predicted to have excellent drug-like properties (e.g., metabolic stability, permeability, blood–brain barrier partition, plasma protein binding and human intestinal absorption properties), which encouraged us to perform further in vitro studies. 

#### 2.2.2. Antiproliferative Activity of Selected β-Lactam Compounds in the MDA-MB-231, Hs578T and Hs578Ts(i)8 Triple Negative Breast Cancer Cell Lines

Selected β-lactam compounds were next evaluated in the triple negative cell line MDA-MB-231 (Table 4). A total of 10–15% of breast cancers are classified as triple-negative breast cancers (TNBC) and include any breast cancer that does not express the genes for oestrogen and progesterone receptors (ER/PR) and HER2. In addition, MDA-MB-231 cells possess mutant p53. These cancers are difficult to treat since they are generally not responsive to hormone therapies such as the selective oestrogen receptor modulator (SERM) tamoxifen, or aromatase inhibitors such as anastrozole, or to the monoclonal antibody Herceptin, which targets the HER2 receptor. There are fewer treatment options available for TNBC compared with ER+, PR+ and HER2+ breast cancers and the prognosis is poorer [77,78]. The 3,3-dichloro-β-lactam **11n** (with 3-hydroxy-4-methoxyphenyl Ring B substitution) was identified as the most potent with IC_50_ = 0.0316 μM, similar to the IC_50_ value of 0.031 μM obtained for **11n** in the MCF-7 cell line. This result compares very favourably with the result obtained for CA-4 in the MDA-MB-231 cell line (IC_50_ = 0.043 μM), and is in good agreement with the reported values for CA-4 in this line [79]. The 3-chloro-β-lactam **10e** (with 4-methoxphenyl Ring B) was found to have slightly less activity in the MDA-MB-231 cells (IC_50_ = 0.0686 μM) but retain a comparable potency in the MCF-7 cell line (IC_50_ = 0.034 μM). The 4-ethoxyphenyl compound **10f** was also impressive in MDA-MB-231 cells with IC_50_ = 0.078 μM, while compounds **10k**, **11e** and **11f** were less active than in the MCF-7 cell lines with IC_50_ values of 0.205, 0.297 and 0.389 μM, respectively.

Compound **10e** was further evaluated in the triple-negative Hs578T breast cancer cell line together with its isogenic subclone Hs578Ts(i)8 cells. Hs578Ts(i)8 cells are three-fold more invasive than the parental cell line (Hs578T) and 2.5-fold more migratory. Hs578Ts(i)8 cells display enhanced invasive properties with 30% more CD44+/CD24-/low cells. They show an increased capacity to proliferate, migrate, invade through the extracellular matrix and produce tumours in nude mice [80]. Compound **10e** demonstrated significant anti-proliferative activity at nanomolar concentrations in Hs578T cells (IC_50_ 124 nM) with increased potency in the invasive Hs578Ts(i)8 cells (IC_50_ = 61 nM). These results compared favourably with CA-4 (IC_50_ = 8 nM in Hs578T and 20 nM in Hs578Ts(i)8 cells) and indicated the potential ability of the compound to inhibit tumour invasion and angiogenesis, which are characteristic features of tumour growth and metastasis in aggressive breast cancers. 

#### 2.2.3. Antiproliferative Activity of Selected β-Lactam Compounds in Multiple Myeloma (U266), Acute Myeloid Leukaemia (HL60) and Colon Cancer (HT-29 and SW480) Cell Lines

Compounds **10n** and **10e** were next evaluated for antiproliferation in multiple myeloma (U266) cells. Multiple myeloma, also known as plasma cell myeloma, is a malignant haematological disease characterised by the proliferation of clonal plasma cells predominantly in the bone marrow. U266 cells are considered to be the least sensitive multiple myeloma cells to nucleoside drug cladribine compared to RPMI8226 and MM1.S cells [81]. Compounds **10n** and **10e** as well as CA-4, demonstrated potent antiproliferative activity in the nanomolar range, with an IC_50_ value of 77 nM for the 3-chloro compound **10e** (with 4-methoxyphenyl Ring B substitution) and a more potent result for the 3-chloro analogue (with 3-hydroxy-4-methoxyphenyl Ring B substitution) **10n**, with IC_50_ = 31 nM, which compares favourably with CA-4 (IC_50_ = 35 nM) in this cell line, Table 5. These results demonstrated the sensitivity of U266 cells toward CA-4 and its 3-chloroazetidinone analogues **10e** and **10n**. 

The 3-chloro β-lactam **10n** was next evaluated in the HL-60 cell line (acute myeloid leukaemia), the colon adenocarcinoma cell line SW480 and also in the HT-29 human colorectal adenocarcinoma cell line. **10n** demonstrated a potent effect with IC_50_ values of 10, 37 and 631 nM, respectively, that compared favourably with the IC_50_ values for CA-4 we obtained in these cell lines of 2 nM (HL-60), 3 nM (SW480) and 4.165 mM (HT-29), respectively (Table 5) and are in agreement with the reported values for CA-4 in MCF-7 and human breast cancer and leukaemia cell lines [74,82,83]. The corresponding values for **10e** in these cell lines were 161 nM (HL-60), 55 nM (SW480) and 135 nM (HT-29). The 3,3-dichloro β-lactam **11n** was slightly less potent than **10n** in these cell lines with IC_50_ values of 16 nM (HL-60), 44 nM (SW480) and 941 nM (HT-29), respectively. These results are interesting as the control drug CA-4 (IC_50_ value of 4.165 μM) is much less active than compounds **10n** and **11n** in the chemoresistant HT-29 cell line. This effect may be due to the inactivation of CA-4 by glucuronidation in HT-29 cells, as previously reported [74]. SW480 colon cells expressed low levels of the UDP-glucuronosyltransferase (UGT) protein compared to expression levels in HT-29 cells. The 3-bromoazetidinones **16a**-**h** and **16j** demonstrated significantly less potent activity in the SW480 colon cancer cells, with **16a**, **16b** and **16j** being the most effective with a 52, 50 and 48% inhibition of cell viability when evaluated at a 10 μM concentration, Table 3. The antiproliferative results for the most potent compound **10n** in cell lines MCF-7, HL-60, SW480, HT-29, HL-60 and U266 are summarised in Table 5, together with the lead compound **10e** and CA-4.

#### 2.2.4. NCI Cell Line Screening for β-Lactam Compounds **10e**, **11n** and **16d**

The novel 3-haloazetidinone compounds **10e**, **11n** and **16d** were selected by NCI for further biological evaluation in the NCI 60 cell line screen following an initial Tier 1 profiling screen [84]. The results obtained for the 3-haloazetidinone compounds **10e**, **11n** and **16d** in the NCI 60 cancer cell line screening (GI_50_ values, five doses) are shown in Table 6. (GI_50_ is the concentration of the compound required to produce 50% of the maximal inhibition of cell proliferation.) The compounds showed broad-spectrum antiproliferative activity against leukaemia, non-small cell lung, colon, CNS, melanoma, ovarian, renal, breast and prostate cancer cell lines and confirmed the results obtained with compounds **10e**, **11n** and **16d** in MCF-7 cells in our laboratory with GI_50_ values of 34, 31 and 44 nM, respectively. The mean GI_50_ values over the full 60 cell line panel for compounds **10e** and **11n** of 74.13 and 53.70 nM, respectively, compare very favourably with CA-4 (GI_50_ value 99 nM), while the mean GI_50_ value for the 3-bromo **16d** is 407 nM. The 3,3-dichloroazetidinone CA-4 analogue **11n** displayed potent activity in the NCI screen, with a mean GI_50_ value of 53.7 nM for all the NCI cell lines tested; these values were in the sub-micromolar range for all the cell lines tested except for the breast cancer cell line T-47D, where the progesterone receptors are not regulated by estradiol [85]. For compound **11n**, the GI_50_ values obtained were below 100 nM for 43 of the cell lines investigated. Compound **11n** was particularly effective against the non-small cell lung (GI_50_ value 16.4–46.4 nM), CNS (GI_50_ value 19.9–51.4 nM), prostate (GI_50_ value 35.3–35.9 nM) cancer cell lines and also in the breast cancer cell lines tested (22.6–42.8 nM) apart from T-47D. The mean LC_50_ values (concentration required to kill 50% of the cells) over 60 cell lines for the potent compounds **10e** and **11n** were 91.20 and 83.18 μM, respectively (Table 7), and were greater than 100 μM in all but one cell line for **10e** and greater than 100 μM in all but four cell lines examined for compound **11n**, indicating low toxicity and suggesting that these compounds may be suitable for therapeutic development.

From the results obtained above, it is apparent that the inclusion of the chloro substituent on the β-lactam scaffold (as in compound **10n**) results in greater antiproliferative effects in the MCF-7 cell line (IC_50_ = 0.0175 μM) than the corresponding 3,3-dichloro compound (**11n**) (IC_50_ = 0.031 μM). By comparison, the introduction of a 3-bromo substituent on the β-lactam scaffold resulted in decreased antiproliferative activity, e.g., compound **16a** (IC_50_ = 0.579 μM) compared with the corresponding 3-chloro compound **10e** (IC_50_ = 0.034 μM). The antiproliferative activity of the most potent β-lactam compounds **10e**, **10n** and **11n** may be correlated to the logP values for some compounds (see Table 3 and Supplementary Information, Tables S1 and S2). The most potent 3-chloro compound **10n** (IC_50_ = 0.0175 μM) has a lower logP (2.666) when compared to the 3,3-dichloro compound **11n** (IC_50_ = 0.031 μM) (logP 3.85); and the 3-bromo compound **16a** (IC_50_ = 0.579 μM) (logP 3.543) has a higher logP value when compared with the corresponding 3-chloro compound **10e** (IC_50_ = 0.034 μM) logP = 3.40, suggesting that compounds with higher logP values displayed poorer activity. However, 3-chloro-4-(2-naphthyl) compound **10k** (IC_50_ = 0.20 μM) and 3,3-dichloro-4-(2-naphthyl) compound **11k** (IC_50_ = 0.322 μM) retained potency, although with higher logP values (4.66 and 5.84, respectively). Interestingly, the 2-naphthyl **10k** was much more potent (IC_50_ = 0.20 μM) than the 1-naphthyl compound **10j** (IC_50_ = 14.66 μM), both with the same logP value (4.66); a similar trend was observed in 3,3-dichloro-1-naphthyl compounds **11j** (IC_50_ = 7.990 μM) and 2-naphthyl compound **11k** (IC_50_ = 0.322 μM), which is possibly related to the steric difficulty in accommodating the 1-naphthyl ring at the colchicine binding site, whereas the 2-naphthyl ring is a better substitute for the 3,4,5-trimethoxyaryl ring A. This effect is also reported for the 1- and 2-naphthyl analogues of CA-4 [86]. 

The GI_50,_ TGI and LC_50_ results of β-lactams **10e**, **11n** and **16d** are summarised in Table 7. The COMPARE algorithm was used to compare the GI_50_ and TGI results for compounds **10e**, **11n** and **16d** with compounds of a known mechanism of action in the NCI Standard Agents Database (Table S3, Supplementary information) and allows correlations in drug sensitivities and molecular targets for biologically active compounds. The highest correlations for potent compounds **10e** and **11n** were obtained for tubulin-targeting agents, including the clinically used vinca alkaloids vincristine sulfate and vinblastine sulfate, together with maytansine. ADC T-DM1 contains an analogue mertansine conjugated with trastuzumab and is used in the treatment of metastatic HER-2 positive breast cancer [3].

#### 2.2.5. Antiproliferative Activity of β-Lactam Compound **10n** in Non-Carcinogenic Human Cells 

**Figure 4:**
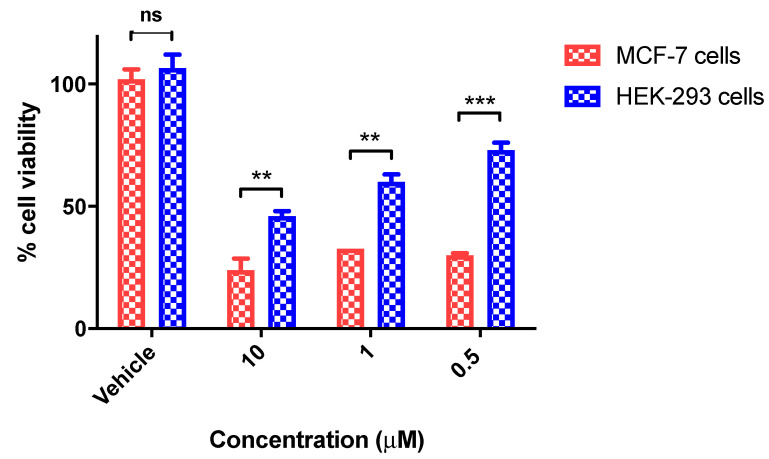
Effect of compound **10n** on the viability of MCF-7 cells and non-tumorigenic HEK-293T cells. Cells were grown in 96-well plates and treated with compound **10n** at 0.5, 1 and 10 μM for 72 h. Cell viability was expressed as a percentage of vehicle control (ethanol 1% (*v*/*v*)) treated cells and was measured using an AlamarBlue assay (average of three independent experiments). Two-Way ANOVA (Bonferroni post-test) was used to test for statistical significance (**, *p* < 0.05, ***, *p* < 0.001).

The toxicity and selectivity of **10n** towards normal cells was investigated in the non-tumourigenic cell line HEK-293T (normal human epithelial embryonic kidney cells). The cell viability of HEK-293T cells was significantly higher than MCF-7 cells following treatment with concentrations of **10n** of 10, 1 and 0.5 μM for 72 h (Figure 4). The IC_50_ value of **10n** in HEK-293 cells (5.5 μM) compared favourably with that observed against the MCF-7 cell line (IC_50_ = 17.5 nM), demonstrating that β-lactam **10n** was less toxic to human normal cells than cancer cells. These data suggested the compound **10n** could be developed as a broad-spectrum anti-cancer agent with lower cytotoxicity to normal cells compared with MCF-7 cancer cells. The cytotoxic effect of selected 3-chloro-β-lactams **10e**, **10f**, **10i**, **10n** and 3,3-dichloro-β-lactams **11e** and **11n** in MCF-7 cells was initially determined using the lactate dehydrogenase (LDH) assay [87]. The 3-chloro-β-lactams produced low cytotoxicity (at 10 μM) over 24 h with 5, 4 and 3% cell death, for compounds **10e**, **10f** and **10i**, respectively, while the 3,3-dichloro compound **11e** demonstrated increased cytotoxicity (10%). Compound **10n** (3-hydroxy-4-methoxyphenyl Ring B), which was the most potent compound in the cell proliferation assays, resulted in increased cytotoxicity (17% cell death), while the similarly substituted 3,3-dichloro compound **11n** displayed lower cytotoxicity of an 8% cell death in this assay. CA-4 (positive control) resulted in a 12% cell death at 10 μM.

#### 2.2.6. β-Lactam Compound 10n Induces Cell Cycle Arrest and Apoptosis in MCF-7 Cells

**Figure 5:**
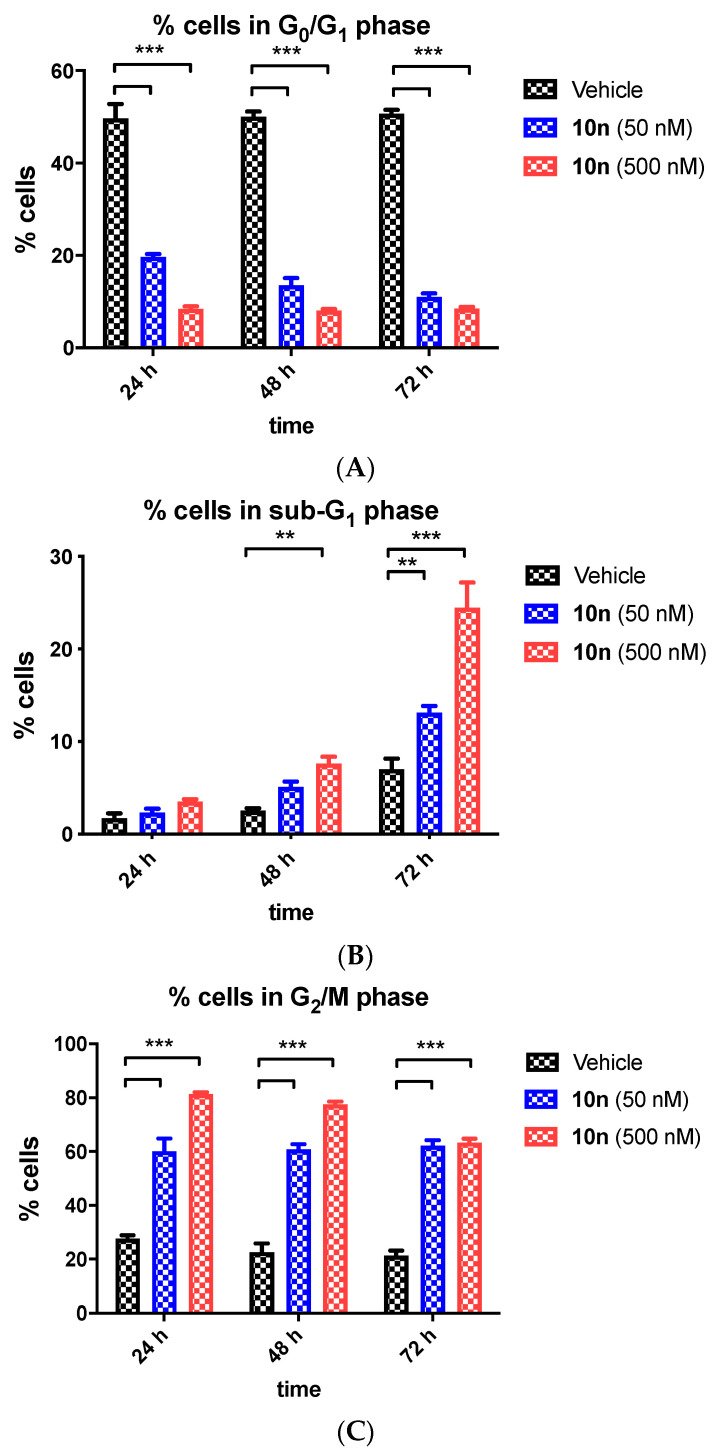
Compound **10n** cells induced G_2_/M arrest followed by apoptosis in a time dependent manner in MCF-7 cells. (**A**) % of cells in G_0_/G_1_ phase, (**B**) % of cells in sub-G_1_ phase and (**C**) % of cells in G_2_/M phase. Cells were treated with either vehicle control (v) (0.1% ethanol (*v*/*v*)) or compound **10n** (50 and 500 nM) for 24, 48 or 72 h. Cells were then fixed, stained with PI and analysed using flow cytometry. Cell cycle analysis was performed on histograms of gated counts per DNA area (FL2-A). The number of cells with <2 N (sub-G_1_), 2 N (G_0_/G_1_) and 4 N (G_2_/M) DNA content was determined with CellQuest software. Values represent the mean ± SEM for three separate experiments. Two-Way ANOVA (Bonferroni post-test) was used to test for statistical significance (**, *p* < 0.05; ***, *p* < 0.001).

To further investigate the mechanism of action of the novel β-lactam compounds synthesised, the effect of β-lactam compound **10n** was investigated on the cell cycle profile of MCF-7 cells. Flow cytometry and propidium iodide (PI) staining facilitate the quantification of the percentage of cells in each phase of the cell cycle (Figure 5). The values obtained for the percentage of cells in G_0_/G_1_, sub-G_1_ (indicative of apoptosis) and the G_2_/M phases of the cell cycle were quantified (at 50 and 500 nM concentrations) and at three time points (24, 48 and 72 h), as shown in Figure 5. The percentage of cells in the G_2_/M phase (81.3% (24 h), 77.5 (48 h) and 63.4% (72 h)) following treatment with sample **10n** (500 nM) was substantially greater than for the control sample treated with the vehicle (21–27%). The observed induction of G_2_/M cell cycle arrest suggests that compound **10n** is an inhibitor of tubulin polymerisation. The percentage of cells undergoing apoptosis (sub-G_1_) increases at all three time points to 25% at 72 h (500 nM) compared to the basal apoptosis level of 7% observed with the vehicle ethanol at 72 h. The percentage of cells in the G_0_G_1_ phase was observed at 8.5% (500 nM), while the untreated cells were 50.7% at 72 h, indicating that the cells are coming out of the G_0_G_1_ phase and are undergoing G_2_/M followed by apoptosis. Similar effects on the cell cycle of MCF-7 cells were observed for the control drug CA-4 with a significant increase in the percentage of cells in G_2_M arrest (52%, 100 nM) with an increase in apoptosis (sub-G_1_) (9.4%) [88]. In summary, compound **10n** was found to induce G_2_/M arrest in MCF-7 cells in a time dependent manner, followed by apoptosis.

**Figure 6:**
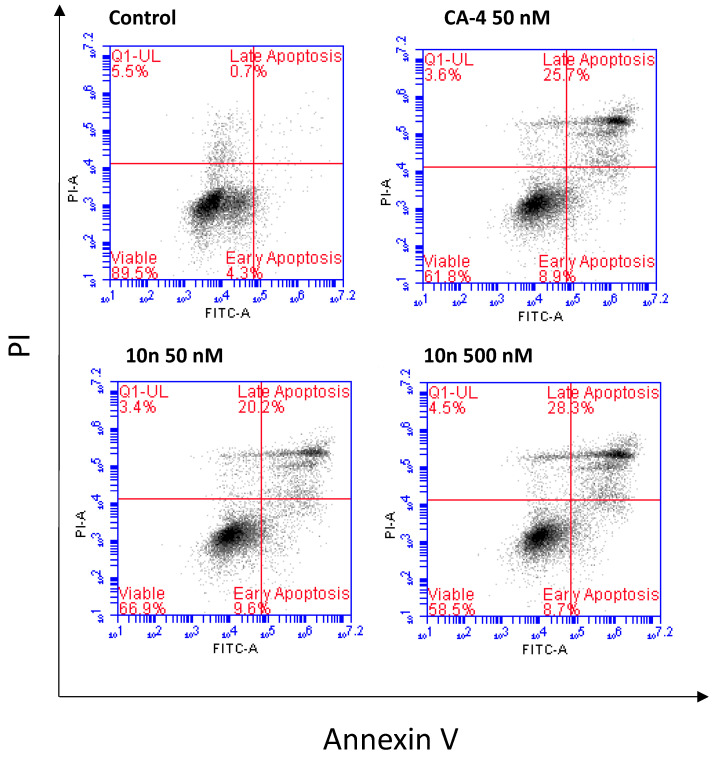
Compound **10n** induced apoptosis in MCF-7 breast cancer cells at 48 h. MCF-7 breast cancer cells were treated with **10n** (50 and 500 nM) or CA-4 (50 nM) or control vehicle (0.1% ethanol (*v*/*v*)). The percentage of apoptotic cells was determined by staining with Annexin V-FITC and propidium iodide (PI). In each panel, the upper left quadrant shows only PI cells that are necrotic, while the lower left quadrant shows cells that are negative for both PI and Annexin V-FITC. The upper right quadrant shows both Annexin/PI positive, which are in late apoptosis/necrosis, while the lower right quadrant shows Annexin positive cells that are in the early apoptotic stage. Cells treated with CA-4 (50 nM) and **10n** (50 and 500 nM) and control cells at 48 h are shown, respectively. Values represent the mean of three independent experiments.

To further investigate the effects of compound **10n** on the induction of cellular apoptosis, MCF-7 cells were treated with compound **10n** for 48 h and then stained with Annexin V-fluorescein isothiocyanate (FITC)/propidium iodide (PI). Following analysis using flow cytometry, differentiation between live cells (annexin-V^−^/PI^−^), early apoptotic cells (annexin- V^+^/PI^−^), late apoptotic cells (annexin-V^+^/PI^+^) and necrotic cells (annexin-V^−^/PI^+^) is possible with dual staining with Annexin-V and PI, see Figure 6. Compound **10n** induced both early and late apoptosis in MCF-7 cells in a concentration-dependent manner when compared to the untreated control cells (Figure 6). When MCF-7 cells were treated with **10n**, the total apoptotic cells (Annexin V-stained positive cells) increased in a dose-dependent manner from 29.8% at 50 nM to 37% at 500 nM. In contrast, only 5.0% of the total apoptotic cells were detected in the control cells (0.1% ethanol (*v*/*v*) treated sample). In comparison, the Annexin V-stained positive cells (total apoptotic) cells for CA-4 were determined as 34.6% in MCF-7 cells at 50 nM, as shown in Figure 6. These results demonstrated that compound **10n** induced cell apoptosis in MCF-7 breast cancer cells.

#### 2.2.7. Effects of Compound **10n** on Tubulin Polymerisation in MCF-7 Cells

**Figure 7:**
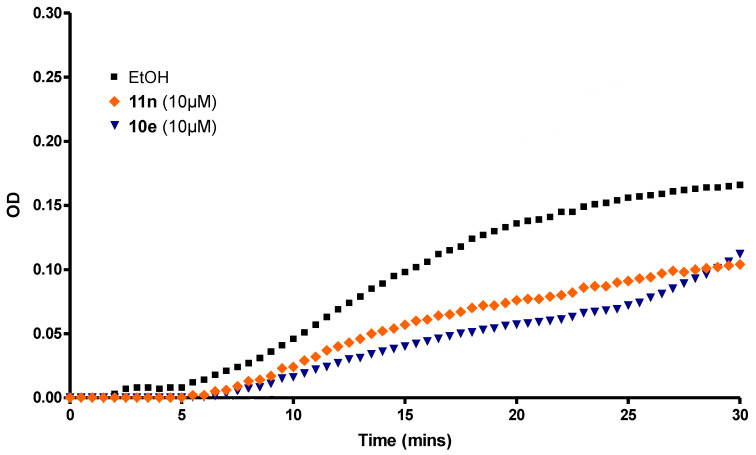
Tubulin polymerisation assay for compounds **10e** and **11n**. Compounds **10e** and **11n** induce depolymerisation of tubulin in vitro. Effect of compounds **10e** and **11n** on in vitro tubulin polymerisation: compounds **10e** and **11n** (10 µM concentration) were first mixed with purified bovine tubulin in a 96-well plate on ice. Purified bovine tubulin and GTP were mixed in a 96-well plate at 37 °C. Ethanol (1% *v*/*v*) was used as a vehicle control. The final concentration of bovine tubulin was 3 mg/mL and glycerol was 10%. After placing the plate into a 37 °C incubator, the effect on tubulin assembly (OD—optical density) was monitored in a Spectramax 340 PC spectrophotometer for 30 min at 37 °C and 340 nm in 30 s intervals. Percentage inhibition of polymerisation was recorded for each reaction. The results represent the mean for three separate experiments performed in duplicate.

The tubulin binding activities of potent compounds **10e** and **11n** evaluated in MCF-7 cells were carried out using a tubulin polymerisation assay kit from Cytoskeleton (BK006P) [89]. In this assay, light is scattered by microtubules to an extent that is proportional to the concentration of the microtubule polymer. The compounds were tested at 10 µM concentration with purified and unpolymerised tubulin. The results for the selected β-lactam compounds are shown in Table 8. The initial assay performed established the effects of compounds **10e** and **11n** on tubulin polymerisation for 30 min (Figure 7, Table 8). Ethanol and CA-4 (**2a**) were used as a vehicle and a positive control, respectively. CA-4 is one of the most effective anti-tubulin natural products. Both compounds **10e** and **11n** showed moderate tubulin polymerisation inhibition effects, although they were less effective than CA-4. When evaluated at a 10 µM concentration, the 3-chloro and 3,3-dichloro compounds reduced the V_max_ value for the rate of tubulin polymerisation 1.7-fold for compound **11n** and 1.8-fold for compound **10e**, whereas CA-4 induced a 6.3-fold reduction (Figure 7, Table 8 and Supplementary Information Figure S1). In general, the tubulin polymerisation inhibition activities of selected compounds depend on the substitutions at the C-3 and C-4 position of β-lactam core. The compounds with chloro and dichloro substituents at the C-3 position, such as **10e** and **11n**, exhibited moderate tubulin polymerisation inhibition and demonstrated some correlation with the antiproliferative effects of these compounds.

**Figure 8:**
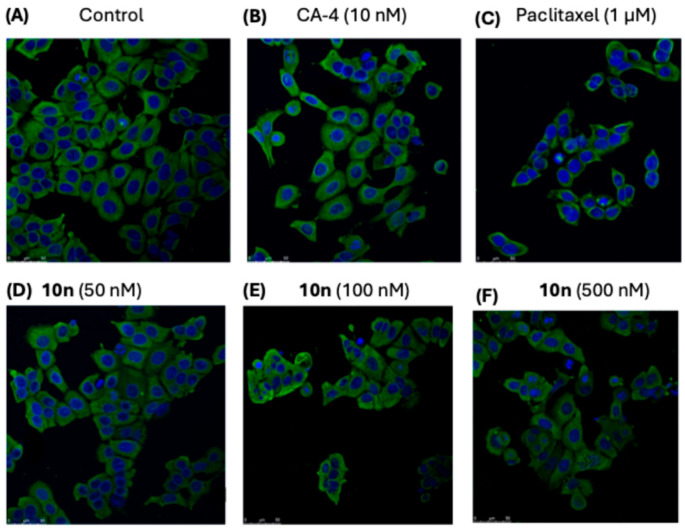
Compound **10n** depolymerises the microtubule network of MCF-7 breast cancer cells. Cells were treated with (**A**) vehicle control (0.1% ethanol (*v*/*v*)), (**B**) CA-4 (10 nM), (**C**) paclitaxel (1 μM) and compound **10n**, (**D**) (50 nM), (**E**) (100 nM) and (**F**) (500 nM) for 16 h. Cells were fixed in ice-cold methanol and stained with mouse monoclonal anti-α-tubulin–FITC antibody (clone DM1A) (green) and Alexa Fluor 488 dye, and counterstained with DAPI (blue). Images were obtained with Leica SP8 confocal microscopy with Leica application suite X software. Representative confocal micrographs of three separate experiments are shown. Scale bar indicates 25 μm.

Subsequently, the effect of the representative compound **10n** on the organisation of microtubule cytoskeleton of MCF-7 cells was also determined by confocal microscopy using an anti-tubulin antibody (Figure 8). As expected, the MCF-7 cells exhibited a well-organised microtubular network (stained green) when treated with the vehicle control (0.1% ethanol). The MCF-7 cell nuclei (stained blue) were also clearly observed (Figure 8A). In contrast, the fibrous microtubule structures were disorganised, and their densities were also significantly reduced by treating the MCF-7 cells with compound **10n**. The paclitaxel-treated sample (Figure 8C) showed the hyper-polymerisation of tubulin, while the extensive depolymerisation of tubulin was demonstrated in the CA-4-treated sample Figure 8B. Cells treated with the β-lactam compound **10n** (0.05, 0.1 and 0.5 μM) displayed a disorganised microtubule network with similar effects to CA-4, together with multinucleation (Figure 8D–F). The treatment of MCF-7 breast cancer cells with CA-4 tubulin-targeting agents has been reported to result in the formation of multiple micronuclei and mitotic catastrophes [90,91,92]. These immunofluorescence studies for the visualisation of the microtubule network in MCF-7 cells confirmed that compound **10n** could directly inhibit the tubulin polymerisation. 

**Figure 9:**
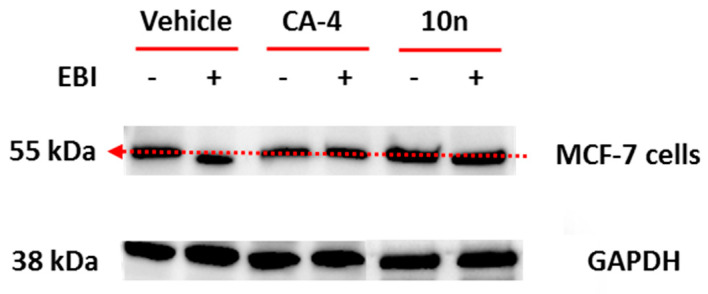
Effects of compound **10n** on the inhibition of the bisthioalkylation of Cys239 and Cys354 of β-tubulin by *N*,*N*′-ethylene-bis(iodoacetamide) (EBI) in MCF-7 cells. MCF-7 was treated with vehicle control (ethanol 0.1% (*v*/*v*)), **CA-4** and **10n** (10 μM) for 2 h; selected samples were then treated with EBI (100 μM) for an additional 1.5 h. Cells were harvested, lysed and analysed using sedimentation and Western blotting for β-tubulin and β-tubulin-EBI adducts. Results are indicative of two separate experiments, performed independently. To confirm equal protein loading, each membrane was stripped and re-probed with a GAPDH antibody.

A colchicine-site binding assay was performed to evaluate the interaction of compound **10n** at the colchicine binding site of tubulin using *N*,*N*-ethylenebis(iodoacetamide) (EBI) [93,94]. EBI crosslinks the Cys-239 and Cys-354 residues of the colchicine binding site of β-tubulin, alkylating the sulfhydryl group of cysteine. This covalent EBI adduct occupies the colchicine binding site of β-tubulin and can be detected using Western blotting as it appears at a lower position than tubulin, indicating that Cys239 and Cys354 amino acids of β-tubulin are crosslinked with EBI. Microtubule targeting compounds binding at the colchicine site, e.g., colchicine and CA-4, prevent the formation of the β-tubulin-EBI adduct. MCF-7 cells were treated with **10n** (10 μM) or CA-4 (10 μM), followed by EBI (100 μM). Control samples (ethanol 0.1% (*v*/*v*)) indicated the formation of the β-tubulin-EBI adduct at a slightly lower position (Figure 9). Tubulin EBI adduct formation was inhibited in the MCF-7 cells treated with CA-4 and **10n**, indicating that both CA-4 and **10n** bind to the colchicine binding site of tubulin. These tubulin polymerisation inhibitors act on the colchicine site of tubulin and compete with EBI to bind the colchicine binding site and hinder the cross-linking of EBI with β-tubulin. 

#### 2.2.8. Effects of Compound **10n** on Expression Levels of Apoptosis-Associated Proteins Bax, Bcl-2 and Mcl-1 in MCF-7 Cells

**Figure 10:**
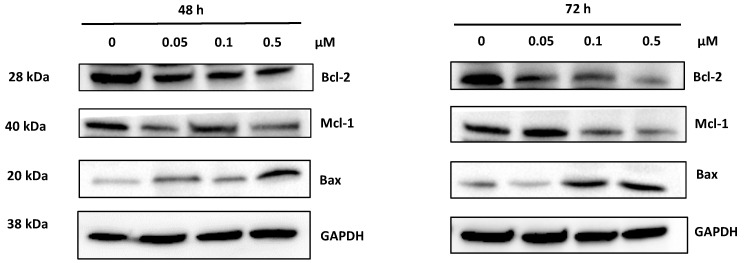
Effects of compound **10n** on expression of pro-apoptotic protein Bax and anti-apoptotic proteins Bcl-2 and Mcl-1 in MCF-7 cells. MCF-7 cells were treated with either vehicle control (ethanol, 0.1% *v*/*v*) or with compound **10n** (0.05, 0.1 and 0.51 μM) for 48 or 72 h. After the required time, the cells were then harvested and separated using SDS PAGE to detect the level of the pro-apoptotic protein Bax and anti-apoptotic proteins Bcl-2 and Mcl-1. Results are representative of two separate experiments performed independently. To confirm equal protein loading, each membrane was stripped and re-probed with a GAPDH antibody.

The effects of compound **10n** on the expression of the pro-apoptotic protein Bax and anti-apoptotic proteins Bcl-2 and Mcl-1 were next investigated using Western blot analysis. The Bcl-2 family of proteins controls and regulates the intrinsic or mitochondrial apoptotic pathway. Pro- and anti-apoptotic members of the Bcl-2 family can oligomerise at the mitochondrial outer membrane to regulate permeabilization, which is a central event in the intrinsic apoptotic pathway. The pro-apoptotic protein Bax, together with Bak, is a key member of the Bcl-2 family and is a core regulator of the intrinsic pathway of apoptosis [95]. Western blot analysis (Figure 10) demonstrated that the expression level of pro-apoptotic protein Bax was upregulated in a dose-dependent manner in MCF-7 cells after treatment with compound **10n** (0.05, 0.1 and 0.5 μM) for 48 or 72 h. 

The apoptosis regulating proteins Bcl-2 and Mcl-1 were next investigated. The anti-apoptotic or pro-survival Bcl-2 protein is also a member of the Bcl-2 family. It prevents the release of a pro-apoptotic AIF (apoptosis inducing factor) and cytochrome c from the mitochondria into the cytoplasm [96] and prevents apoptosis by sequestering caspases (apoptosis promoters). The level of the anti-apoptotic protein Bcl-2 was downregulated in a dose-dependent manner after treating MCF-7 cells with compound **10n** (0.05, 0.1 and 0.5 μM) for 48 or 72 h. (Figure 9). Mcl-1 protein (an induced myeloid leukaemia cell differentiation protein) is another key anti-apoptotic member of the Bcl-2 family and is localised in the mitochondrial outer membrane [97]. It binds and sequesters the pro-apoptotic Bax/Bak proteins and, thus, prevents the release of cytochrome c [98]. Overexpression of the anti-apoptotic factors Mcl-1, Bcl-2 and Bcl-xL in acute myeloid leukaemia [99] and acute lymphocytic leukaemia [100] may be associated dysregulation of apoptosis. The level of the anti-apoptotic protein Mcl-1 was downregulated in a dose-dependent manner after treating MCF-7 cells with compound **10n** (0.05, 0.1 and 0.5 μM) for 48 or 72 h. (Figure 10). Apoptosis can be triggered by a reduction in the expression levels of Mcl-1 and Bcl-2 (e.g., by drug treatment). The increase in the percentage of cells observed in the sub-G_1_ peak, together with the flow cytometry analysis of Annexin V/PI-stained cells support the pro-apoptotic mechanism of action proposed for these compounds (Figure 5 and Figure 6).

### 2.3. Computational Modelling of β-Lactam Compounds ***10n***, ***11n*** and ***14b***

**Figure 11:**
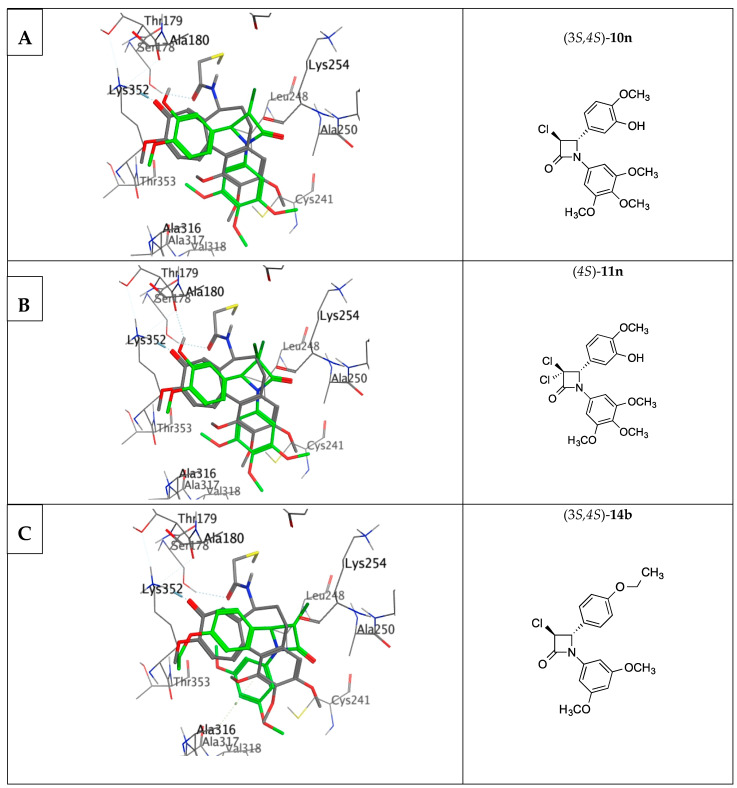
Overlay of the X-ray structure of tubulin co-crystallised with DAMA-colchicine (PDB entry 1SA0) on the best ranked docked poses of the *S* enantiomers of the three studied beta-lactams: (**A**) **10n**, (**B**) **11n** and (**C**) **14b**. Ligands are rendered as tube and amino acids as line. Tubulin amino acids and DAMA-colchicine are coloured by atom type: carbon = grey, hydrogen = white, oxygen = red, nitrogen = blue, sulphur = yellow, bromine = dark red, chlorine = dark green. The beta-lactams are depicted with a green backbone. The atoms are coloured by element type, Key amino acid residues are labelled, and multiple residues are hidden to enable a clearer view.

Computational docking calculations using MOE 2019.01 [101] were undertaken on both enantiomers of the potent compounds *trans*-3-chloro-1-(3,4,5-trimethoxyphenyl)-β-lactam **10n**, 3,3-dichloro-1-(3,4,5-trimethoxyphenyl)-β-lactam **11n** together with the 3-chloro-1-(3,5-dimethoxyphenyl)-β-lactam **14b**, using the X-ray structure of bovine tubulin co-crystallised with N-deacetyl-N-(2-mercaptoacetyl)-colchicine (DAMA-colchicine) 1SA0 [6], Figure 11. ^1^H NMR analysis determined that only the *trans* isomers of the compounds **10n** and **14b** were isolated; therefore, we modelled only the 3*S*,4*S* and 3*R*,4*R* enantiomeric pairs. In all cases, the *S*,*S* enantiomers were more highly ranked than the corresponding *R*,*R* enantiomeric pair; therefore, only they will be discussed. All trimethoxy compounds overlaid their B-rings on the C-ring of DAMA-colchicine (forming HBA interactions with Lys352), co-located the 3,4,5-trimethoxyphenyl substituted A-rings and were able to position the halogens in an open region of the tubulin binding site at the monomer interface. The predicted affinity ranking from best ranked to worst was **10n***SS*, **14b***SS*, **11n***S*, **10n***RR*, **11n***R* and **14b***RR*. Generating conformers with OMEGA [1,2] and running docking with FRED [3] also gave the same preference for *SS* over *RR* enantiomers. Docking studies are not ideal for studying changes in cellular efficacy associated with different halogen substituents. While the *SS* enantiomer of the dimethoxy analogue **14b** presented a comparable binding mode to other analogues in the tubulin site, it did not overlap fully with DAMA-colchicine (Figure 11, Panel C). The lack of a hydroxy group in Ring B of **14b** to potentially hydrogen bond with Lys352 and the added steric bulk, resulting from the substitution of 4-methoxy with 4-ethoxy on the B-ring, forced the molecule deeper into the binding site, resulting in the less favourable docking scores. The increase in lipophilicity could also decrease the cell permeability of **14b**, causing a slight loss in efficacy against MCF-7 cells. Figure 11 illustrates the best ranked binding pose of each compound, showing the shared binding mode across the analogues.

## 3. Materials and Methods

### 3.1. Chemistry

All chemicals were commercially purchased and were used without further purification unless otherwise indicated. Solvents were either purchased dry or purified by distillation in accordance with literature methods. Dichloromethane was dried by distillation from calcium hydride prior to use. Tetrahydrofuran (THF) was distilled immediately prior to use from Na/Benzophenone under nitrogen. Toluene was dried by distillation from calcium hydride and stored on activated molecular sieves (4 Å). Melting points were determined on a Gallenkamp SMP 11 melting point apparatus and are uncorrected. Infra-red (IR) spectra were recorded as KBr discs, thin films on NaCl disk or ATR on a Perkin Elmer FT-IR Paragon 1000 spectrometer. ^1^H and ^13^C nuclear magnetic resonance (NMR) spectra were recorded at 20 °C on a Bruker DPX 400 spectrometer (400.13 MHz, ^1^H; 100.61 MHz, ^13^C) in CDCl_3_, DMSO-*d*_6_ or CD_3_OD by Dr. John O’Brien and Dr. Manuel Ruether in the School of Chemistry, Trinity College Dublin, with internal standard TMS. The chemical shifts are given in ppm relative to Me_4_Si as an internal reference, *J* values are given in Hz. High resolution mass spectrometry (HRMS) was obtained in the School of Chemistry by Dr Martin Feeney or the School of Pharmacy and Pharmaceutical Sciences by Mr Brian Talbot, Trinity College Dublin. HRMS was carried on in the positive ion mode on a liquid chromatography time-of flight mass spectrometer (Micromass LCT, Waters Ltd., Manchester, UK). The samples were introduced into the ion source by an LC system (Waters Alliance 2795, Waters Corporation, Milford, MA, USA) in acetonitrile:water (60:40% *v*/*v*) at 200 μL/min. A lock (reference) mass (*m*/*z* 556.2771) was used. High resolution mass spectrometry scans were also performed using Electrospray Ionisation operated in negative and positive ion modes on an LTQ/Orbitrap Discovery Mass Spectrometer, and samples were dissolved in CH_3_OH, with a mass accuracy of <±5 ppm. Low resolution mass spectra (LRMS) were obtained on a Hewlett-Packard 5973 MSD GC-MS system in electron impact (EI) mode. Thin layer chromatography was performed with Merck silica gel 60 TLC aluminium sheets using a fluorescent indicator visualising at 254 nm in UV. Merck Kiesegel 60 (particle size 0.040–0.063 mm) was used for flash column chromatography. Preparative chromatography was also carried out on a Biotage SP4 instrument. All products isolated were homogenous on TLC. Microwave experiments were performed with the Biotage Initiator and Discover CEM microwave synthesisers. Purity of the finial compounds was achieved using analytical high-performance liquid chromatography (HPLC) using a Waters 2487 Dual Wavelength Absorbance detector, Waters 1525 binary HPLC pump, Waters In-Line Degasser AF and Waters 717plus Autosampler, with a Varian Pursuit XRs C18 reverse phase 250 × 4.6 mm column and detection at 254 nm. Samples were analysed using acetonitrile (60%):water (40%) with 0.1% (*v*/*v*) TFA over 10 min and a flow rate of 1 mL/min. Imines **9a**-**9j**, **9m**–**9s**, **11f**, **11i**, **12e**, **12f** and **12i** were prepared as previously reported [34,35,74,102]. The details for the preparation of 3-azetidinones **10f**, **10i**, **10o**, **11e**, **11f**, **11i** and **11o** were as previously reported [34,74] are provided in the Supplementary Information.

#### 3.1.1. General Method 1A: PREPARATION of Imines with Ethanol as Solvent (**9k**, **9l**, **9t**–**v**):

The appropriately substituted benzaldehyde (10 mmol) and corresponding substituted aniline (10 mmol) were heated at reflux in ethanol (40 mL) for 4 h. The reaction solvent was then reduced to approximately 10 mL *in vacuo* and the reaction mixture was allowed to stand for 12 h. The precipitated product was filtered and then recrystallised from ethanol. 

##### 2-Methoxy-5-[(3,4,5-trimethoxyphenylimino)methyl]phenol (**9k**) 

Compound **9k** was prepared using the general method IA above and was obtained from 3-hydroxy-4-methoxybenzaldehyde and 3,4,5-trimethoxyaniline as a pale-yellow solid; yield: 89%, 2.82 g, Mp. 134 °C [33]. IR V_max_ (KBr) 1613 (C=N), 3347 (OH) cm^−1^. ^1^H NMR (400 MHz, CDCl_3_): δ 3.83 (s, 3H, -OCH_3_), 3.92 (s, 6H, -OCH_3_), 3.98 (s, 3H, -OCH_3_), 5.72 (s, 1H, -OH), 6.49 (s, 2H, H_2′_, H_6′_), 6.95 (d, 1H, *J* = 8.52 Hz, H_5′_’), 7.39 (dd, *J* = 1.48 Hz, 8.28, 1H, H_6′_’), 7.54 (d, *J* = 1.48 Hz, 1H, H_2′_’), 8.38 (s, 1H, CH=N). ^13^C NMR (100 MHz, CDCl_3_): δ 55.61, 55.67, 60.58 (-OCH_3_), 97.96 (C_2_, C_6_), 109.87 (C_5′_’), 113.31 (C_2′_’), 121.85 (C_6′_’), 129.49 (C_1′_’), 136 (C_4′_), 145.51 (C_3′_’), 147.71 (C_1′_), 153.08 (C_4′_’), (C_3′_, C_5′_), 158.72 (CH=N). HRMS: found 318.1353 [M + H]^+^; C_17_H_20_NO_5_ requires 318.1341.

##### [3-(Tert-butyldimethylsilanyloxy)-4-methoxybenzylidene](3,4,5-trimethoxyphenyl)amine (**9l**)

To a solution of the imine **9k** (5 mmol) and *tert*-butyldimethylsilyl chloride (6 mmol) in anhydrous DCM (40 mL) under a nitrogen atmosphere, DBU (8 mmol) was added dropwise via a syringe. Stirring under nitrogen was continued until starting material had disappeared as monitored by TLC over 2–4 h (eluent, 50:50 hexane/ethyl acetate). Upon completion, the reaction was diluted with dichloromethane (50 mL). The reaction mixture was washed with water (2 × 100 mL), 0.1 M HCl aq (2 × 50 mL) and saturated NaHCO_3_ solution (2 × 50 mL) and dried over Na_2_SO_4_. The solvent was removed under reduced pressure to yield the protected Schiff base, which was used for β-lactam synthesis without further purification; yield: 78%, 1.68 g, amber oil [36]. IR V_max_ (film) 1585 cm^−1^ (C=N). ^1^H NMR (400 MHz, CDCl_3_): δ 0.20 (s, 6H, -*t*BDMSi), 1.03 (s, 9H, -*t*BDMSi), 3.77 (s, 3H, -OCH_3_), 3.81 (s, 6H, -OCH_3_), 3.89 (s, 3H, -OCH_3_), 5.94 (s, 2H, H_2′_, H_6′_), 6.48 (s, 1H, H_5′_’), 6.92 (1H, d, *J* = 8.52 Hz, H_2′_’), 7.46 (1H, m, H_6′_’) and 8.35 (1H, s, imine). ^13^C NMR (100 MHz, CDCl_3_): δ -5.67, 18.07, 25.26 (–tBDMSi), 54.99, 55.64, 60.62 (–OCH_3_), 92.12 (C_2′_, C_6′_), 110.96 (C_5′_’), 119.74 (C_2′_’), 123.42 (C_6′_’), 128.95 (C_1′_’), 144.50 (C_4′_), 144.87 (C_3′_’), 147.94 (C_1′_), 153.06 (C_3′_, C_5′_), 153.40 (C_4′_’), 158.82 (C=N). HRMS: found 431.2127 [M]^+^; C_23_H_33_NO_5_Si requires 431.2128.

##### (E)-N-(4-Methoxyphenyl)-1-(3,4,5-trimethoxyphenyl)methanimine (**9t**)

Compound **9t** was prepared using the general method IA above from 4-methoxyaniline and 3,4,5-trimethoxy benzaldehyde as pale-yellow crystals; yield: 70%, Mp. 115 °C [103] (HPLC: 95%). IR V_max_ (ATR): 1622.8 (C=N) cm^−1^. ^1^H NMR (400 MHz, CDCl_3_): δ 3.85 (s, 6H, OCH_3_), 3.90 (s, 6H, OCH_3_), 6.96 (d, *J* = 8.03 Hz, 2H, ArH), 7.18 (s, 2H, ArH), 7.26 (d, *J* = 8.03 Hz, 2H, ArH), 8.40 (s, 1H, CH=N). ^13^C NMR (100 MHz, CDCl_3_): δ 55.0, 55.8, 60.6, 105.1, 106.2, 113.9, 116.2, 131.4, 140.3, 153.0, 157.6 (imine, HC=NC), 161.9. HRMS: found 324.1198 [M + Na]^+^; C_17_H_19_NNaO_4_ requires 324.1212. 

##### (E)-N-(4-Ethoxyphenyl)-1-(3,4,5-trimethoxyphenyl)methanimine (**9u**)

Compound **9u** was synthesised using the general method IA above and was obtained from 4-ethoxyaniline and 3,4,5-trimethoxybenzaldehyde as yellow crystals; yield: 82%, Mp: 98 °C [103] (HPLC: 99%). IR V_max_ (ATR): 1636.4 cm^−1^ (C=N). ^1^H NMR (400 MHz, CDCl_3_): δ 1.41 (t, *J* = 7.02 Hz, 3H, OCH_2_CH_3_), 3.89 (s, 3H, OCH_3_), 3.93 (s, 6H, OCH_3_), 4.04 (q, *J* = 6.71 Hz, 2H, OCH_2_CH_3_), 6.86–6.94 (m, 2H, ArH), 7.12 (s, 2H, ArH), 7.16–7.22 (m, 2H, ArH), 8.35 (s, 1H, CH=N). ^13^C NMR (100 MHz, CDCl_3_): δ 14.85, 56.21, 60.94, 63.66, 97.31, 105.49, 114.93, 122.10, 132.00, 140.64, 153.47, 157.57 (imine, HC=NC), 157.79. HRMS: found 338.1364 [M + Na]^+^; C_18_H_21_NNaO_4_ requires 338.1368. 

##### (E)-N-(4-(Methylthio)phenyl)-1-(3,4,5-trimethoxyphenyl)methanimine (**9v**)

Compound **9v** was synthesised using the general method IA above and was obtained from 4-methylthioaniline and 3,4,5-trimethoxybenzaldehyde as yellow crystals; yield: 92%, Mp. 112 °C [103]. IR V_max_ (ATR): 1677.8 cm^−1^ (C=N). ^1^H NMR (400 MHz, CDCl_3_): δ 2.48 (s, 3H, SCH_3_), 3.86 (s, 3H, OCH_3_), 3.96 (s, 6H, OCH_3_), 7.14 (d, *J* = 8.54 Hz, 2H, ArH), 7.23–7.28 (m, 2H, ArH), 7.12 (s, 2H, ArH), 8.33 (s,1H, CH=N). ^13^C NMR (100 MHz, CDCl_3_): δ 16.34, 56.23, 60.96, 105.71, 121.50, 127.66, 131.69, 135.80, 140.95, 149.17, 153.49, 159.18 (imine, HC=NC). HRMS: found 340.0994 [M + Na]^+^; C_17_H_19_NNaO_3_S requires 340.0983. 

#### 3.1.2. General Method IB: Schiff Base Preparation with Water as a Solvent (**9w**, **9x**)

The appropriately substituted benzaldehyde (10 mmol) and corresponding substituted aniline (10 mmol) were stirred in water (7 mL) for 30 min. The organic compound was extracted with DCM and the reaction mixture was dried over anhydrous sodium sulfate before the solvent was removed under reduced pressure. 

##### (E)-N-(3,5-Dimethoxyphenyl)-1-(4-methoxyphenyl)methanimine (**9w**)

Compound **9w** was prepared using the general method IB above and was obtained from 4-methoxybenzaldehyde and 3,5-dimethoxyaniline as an oil [50]; yield: 97%, IR V_max_ (ATR): 1592.2 cm^−1^ (C=N). ^1^H NMR (400 MHz, CDCl_3_): δ 3.79 (s, 6H, OCH_3_), 3.84 (s, 3H, OCH_3_), 6.30–6.36 (m, 3H, ArH), 6.95 (d, *J* = 7.93 Hz, 2H, ArH), 7.81 (d, *J* = 7.93 Hz, 2H, ArH), 8.35 (s, 1H, CH=N). ^13^C NMR (100 MHz, CDCl_3_): δ 55.37, 55.52, 97.92, 98.97, 114.15, 114.26, 129.00, 130.54, 131.92, 154.52, 159.83 (HC=NC). HRMS: calculated for C_16_H_18_NO_3_ [M + H]^+^ 272.1287; found 272.1289. 

##### (E)-N-(3,5-Dimethoxyphenyl)-1-(4-ethoxyphenyl)methanimine (**9x**)

Compound **9w** was prepared using the general method IB above and was obtained from 4-ethoxybenzaldehyde and 3,5-dimethoxyaniline as an oil; yield: 85%. IR V_max_ (ATR): 1597.6 cm^−1^ (C=N). ^1^H NMR (400 MHz, CDCl_3_): δ 1.43 (t, *J* = 7.02 Hz, 3H, OCH_2_CH_3_), 3.80 (s, 6H, OCH_3_), 4.09 (q, *J* = 7.12 Hz, 2H, OCH_2_CH_3_), 6.22–6.44 (m, 3 H, ArH), 6.90–7.00 (m, 2H, ArH), 7.80 (m, *J* = 8.55 Hz, 2H, ArH), 8.35 (s, 1H, CH=N). ^13^C NMR (100 MHz, CDCl_3_): δ 14.71, 55.40, 63.64, 97.90, 98.97, 114.64, 130.55, 153.22, 159.93 (HC=NC). HRMS: calculated for C_17_H_20_NO_3_ [M + H]^+^ 286.1443; found 286.1456. 

#### 3.1.3. General Method II: Preparation of 3-Chloroazetidin-2-ones, 3,3-Dichloroazetidin-2-ones, 3-Bromoazetidin-2-ones (**10a**–**o**, **11a**–**o**, **12a**–**c**, **13a**–**c**, **14a**, **14b**, **15a**, **15b**, **16a**–**h**)

To a stirring, refluxing solution of the imine (5 mmol) and triethylamine (6 mmol) in anhydrous dichloromethane (40 mL), a solution of chloro- or dichloroacetyl chloride (6 mmol) in anhydrous dichloromethane (10 mL) was injected dropwise through a rubber septum over 45 min under nitrogen. The reaction was refluxed for 5 h, and at retained at 20 °C overnight under nitrogen. The reaction mixture was washed with water (2 × 100 mL); the organic layer was dried over anhydrous sodium sulfate and the solvent was then removed under reduced pressure. The crude product was purified using flash chromatography over silica gel (eluent 4:1 *n*-hexane: ethyl acetate).

##### 3-Chloro-1-(3,4,5-trimethoxyphenyl)-4-phenylazetidin-2-one (**10a**)

Compound **10a** was prepared as described in the general method II above from imine **9a** and chloroacetyl chloride; yield: 8%, 135 mg, brown oil (HPLC: 100%). IR (NaCl, film) V_max_: 2983, 2684, 1764 (C=O, β-lactam), 1601, 1507, 1235, 1127 cm^−1^. ^1^H NMR (400 MHz, CDCl_3_): δ 3.71 (s, 6H, OCH_3_), 3.79 (s, 3H, OCH_3_), 4.67 (br s, 1H, H_4_), 4.99 (br s, 1H, H_3_), 6.54 (s, 2H, H_2′_ H_6′_), 7.40–7.46 (m, 5H, H_2′_’ H_3′_’ H_4′_’ H_5′_’ H_6′_’). ^13^C NMR (100 MHz, CDCl_3_): δ 55.58, 60.52 (OCH_3_), 62.63 (C_3_), 65.97 (C_4_), 94.74 (C_2′_, C_6′_), 125.74 (C_2′_’, C_6′_’), 129.06 (C_3′_’, C_5′_’), 129.19 (C_4′_’), 132.46 (C_4′_), 134.52 (C_1′_), 134.65 (C_1′_’), 153.10 (C_3′_, C_5′_), 160.15 (C_2_). HRMS: found 370.0822 [M + Na]^+^; C_18_H_18_^35^ClNO_4_Na requires 370.0822.

##### 3-Chloro-4-(4-chlorophenyl)-1-(3,4,5-trimethoxyphenyl)azetidin-2-one (**10b**)

Compound **10b** was prepared as described in the general method II above from imine **9b** and chloroacetyl chloride; yield: 9%, 169 mg, brown oil. IR (NaCl, film) V_max_: 2605, 2498, 1766 (C=O, β-lactam), 1595, 1506, 1235, 1127 cm^−1^. ^1^H NMR (400 MHz, CDCl_3_): δ 3.73 (s, 6H, OCH_3_), 3.78 (s, 3H, OCH_3_), 4.61 (d, 1H, *J* = 1.48 Hz, H_4_), 4.98 (d, 1H, *J* = 2.00 Hz, H_3_), 6.51 (s, 2H, H_2′_ H_6′_), 7.35 (d, *J* = 8.52 Hz, 2H, H_3′_’ H_5′_’), 7.43 (d, *J* = 8.56 Hz, 2H, H_2′_’ H_6′_’). ^13^C NMR (100 MHz, CDCl_3_): δ 55.64, 60.51 (OCH_3_), 62.56 (C_3_), 65.16 (C_4_), 94.72 (C_2′_, C_6′_), 127.03 (C_2′_’, C_6′_’), 129.32 (C_3′_’, C_5′_’), 132.19 (C_4′_’), 133.05 (C_4′_), 134.84 (C_1′_), 135.12 (C_1′_’), 153.19 (C_3′_, C_5′_), 159.84 (C_2_). HRMS: found 404.0449 [M + Na]^+^; C_18_H_17_^35^Cl_2_NO_4_Na requires 404.0432.

##### 4-(4-Bromophenyl)-3-chloro-1-(3,4,5-trimethoxyphenyl)azetidin-2-one (**10c**)

Compound **10c** was prepared as described in the general method II above from imine **9c** and chloroacetyl chloride; yield: 9%, 192 mg, brown oil (HPLC: 97.5%). IR (NaCl, film) V_max_: 2606, 2499, 1766 (C=O, β-lactam), 1591, 1506, 1235, 1128 cm^−1^. ^1^H NMR (400 MHz, CDCl_3_): δ 3.74 (s, 6H, OCH_3_), 3.79 (s, 3H, OCH_3_), 4.61 (br s, 1H, H_4_), 4.97 (br s, 1H, H_3_), 6.51 (s, 2H, H_2′_ H_6′_), 7.29 (d, *J* = 8.52 Hz, 2H, H_2′_’ H_6′_’), 7.59 (d, *J* = 8.56 Hz, 2H, H_3′_’ H_5′_’). ^13^C NMR (100 MHz, CDCl_3_): δ 55.67, 60.52 (OCH_3_), 62.49 (C_3_), 65.23 (C_4_), 94.72 (C_2′_, C_6′_), 123.25 (C_4′_’), 127.28 (C_2′_’, C_6′_’), 132.17 (C_4′_), 132.29 (C_3′_’, C_5′_’), 133.57 (C_1′_), 134.87 (C_1′_’), 153.20 (C_3′_, C_5′_), 159.83 (C_2_). HRMS: found 447.9915 [M + Na]^+^; C_18_H_17_^80^Br^35^ClNO_4_Na requires 447.9927.

##### 3-Chloro-1-(3,4,5-trimethoxyphenyl)-4-(4-nitrophenyl)azetidin-2-one (**10d**)

Compound **10d** was prepared as described in the general method II above from imine **9d** and chloroacetyl chloride; yield: 7%, 142 mg, yellow oil (HPLC: 97.5%). IR (NaCl, film) V_max_: 2606, 2499, 1768 (C=O, β-lactam), 1603, 1507, 1235, 1128 cm^−1^. ^1^H NMR (400 MHz, CDCl_3_): δ 3.74 (s, 6H, OCH_3_), 3.79 (s, 3H, OCH_3_), 4.65 (d, *J* = 2.00 Hz, 1H, H_4_), 5.13 (br s, 1H, H_3_), 6.49 (s, 2H, H_2′_ H_6′_), 7.61 (d, *J* = 8.52 Hz, 2H, H_2′_’ H_6′_’), 8.33 (d, *J* = 9.04 Hz, 2H, H_3′_’ H_5′_’). ^13^C NMR (100 MHz, CDCl_3_): δ 55.73, 60.54 (OCH_3_), 62.42 (C_3_), 64.71 (C_4_), 94.72 (C_2′_, C_6′_), 124.33 (C_2′_’, C_6′_’), 126.64 (C_3′_’, C_5′_’), 131.85 (C_4′_), 135.19 (C_1′_), 141.60 (C_4′_’), 148.17 (C_1′_’), 153.36 (C_3′_, C_5′_), 159.29 (C_2_). HRMS: found 415.0672 [M + Na]^+^; C_18_H_17_^35^ClN_2_O_6_Na requires 415.0673.

##### *Cis*-3-chloro-1-(3,4,5-trimethoxyphenyl)-4-(4-methoxyphenyl) azetidin-2-one (**10e *cis***)

Compound **10e *cis*** was prepared as described in the general method II above from imine **9e** and chloroacetyl chloride; yield: 4%, 97 mg, brown oil. IR (NaCl, film) V_max_: 2944, 2605, 1762 (C=O, β-lactam), 1601, 1508, 1240, 1127 cm^−1^. ^1^H NMR (400 MHz, CDCl_3_): δ 3.74 (s, 6H, OCH_3_), 3.79 (s, 3H, OCH_3_), 3.84 (s, 3H, OCH_3_), 5.03 (d, *J* = 5.02 Hz, 1H, H_4_), 5.36 (d, *J* = 5.00 Hz, 1H, H_3_), 6.57 (s, 2H, H_2′_ H_6′_), 6.86 (d, *J* = 8.52 Hz, 2H, H_3′_’ H_5′_’), 7.03 (d, *J* = 9.04 Hz, 2H, H_2′_’ H_6′_’). ^13^C NMR (100 MHz, CDCl_3_): δ 54.75, 55.16, 60.21 (OCH_3_), 60.73 (C_3_), 63.66 (C_4_), 94.75 (C_2′_, C_6′_), 113.78 (C_3′_’, C_5′_’), 126.09 (C_2′_’, C_6′_’), 131.51 (C_4′_), 131.90 (C_1′_’), 134.31 (C_1′_), 153.06 (C_3′_, C_5′_), 159.95 (C_4′_’), 161.01 (C_2_). HRMS: found 578.1107 [M + H]^+^; C_19_H_21_^35^ClNO_5_ requires 378.1108.

##### *Trans*-3-chloro-1-(3,4,5-trimethoxyphenyl)-4-(4-methoxyphenyl) azetidin-2-one (**10e *trans***)

Compound **10e *trans*** was also isolated as described in the general method II above from imine **9e** and chloroacetyl chloride; yield: 8%, 181 mg, brown oil [74]. IR (NaCl, film) V_max_: 2954, 2603, 1772 (C=O, β-lactam), 1681, 1507, 1235, 1126 cm^−1^. ^1^H NMR (400 MHz, CDCl_3_): δ 3.72 (s, 6H, OCH_3_), 3.78 (s, 3H, OCH_3_), 3.84 (s, 3H, OCH_3_), 4.63 (d, *J* = 2.00 Hz, 1H, H_4_), 4.95 (d, *J* = 2.00 Hz, 1H, H_3_), 6.54 (s, 2H, H_2′_ H_6′_), 6.96 (d, *J* = 8.52 Hz, 2H, H_3′_’ H_5′_’), 7.33 (d, *J* = 9.04 Hz, 2H, H_2′_’ H_6′_’). ^13^C NMR (100 MHz, CDCl_3_): δ 54.95, 55.60, 60.51 (OCH_3_), 62.73 (C_3_), 65.66 (C_4_), 94.78 (C_2′_, C_6′_), 114.38 (C_3′_’, C_5′_’), 127.09 (C_2′_’, C_6′_’), 131.56 (C_4′_), 132.50 (C_1′_’), 134.61 (C_1′_), 153.08 (C_3′_, C_5′_), 160.05 (C_4′_’), 160.29 (C_2_). HRMS: found 378.1104 [M + H]^+^; C_19_H_21_^35^ClNO_5_ requires 378.1108.

##### 3-Chloro-1-(3,4,5-trimethoxyphenyl)-4-(4-phenoxyphenyl)azetidin-2-one (**10g**) 

Compound **10g** was prepared as described in the general method II above from imine **9g** and chloroacetyl chloride; yield: 8%, 178 mg, brown oil. IR (NaCl, film) V_max_: 2982, 2682, 1764 (C=O, β-lactam), 1692, 1585, 1506, 1127 cm^−1^. ^1^H NMR (400 MHz, CDCl_3_): δ 3.75 (s, 6H, OCH_3_), 3.80 (s, 3H, OCH_3_), 4.66 (d, *J* = 1.52 Hz, 1H, H_4_), 4.98 (br s, 1H, H_3_), 6.55 (s, 2H, H_2′_ H_6′_), 7.02–7.07 (m, 4H, H_2′_’ H_6′_’ H_2′_’’ H_6′_’’), 7.16–7.20 (m, H_4′_’’), 7.36–7.40 (m, 4H, H_2′_’ H_6′_’ H_3′_’’ H_5′_’’). ^13^C NMR (100 MHz, CDCl_3_): δ 55.63, 60.53 (OCH_3_), 62.69 (C_3_), 65.51 (C_4_), 94.82 (C_2′_, C_6′_), 118.72 (C_2′_’’, C_6′_’’), 119.00 (C_3′_’, C_5′_’), 123.66 (C_4′_’’), 127.28 (C_2′_’, C_6′_’), 128.77 (C_4′_), 129.52 (C_3′_’’, C_5′_’’), 132.40 (C_1′_), 134.73 (C_1′_’), 153.13 (C_3′_, C_5′_), 155.76 (C_4′_’), 158.23 (C_1′_’’), 160.14 (C_2_). HRMS: found 462.1099 [M + Na]^+^; C_24_H_22_^35^ClNO_5_Na requires 462.1084.

##### 4-(4-Benzyloxyphenyl)-3-chloro-1-(3,4,5-trimethoxyphenyl)azetidin-2-one (**10h**) 

Compound **10h** was prepared as described in the general method II above from imine **9h** and chloroacetyl chloride; yield: 3%, 70 mg, colourless solid (HPLC: 100.0%), Mp 96–98 °C. IR (KBr) V_max_**:** 2944, 1761 (C=O, β-lactam), 1597, 1508, 1237, 1127 cm^−1^. ^1^H NMR (400 MHz, CDCl_3_): δ 3.72 (s, 6H, OCH_3_), 3.79 (s, 3H, OCH_3_), 4.64 (d, *J* = 2.00 Hz, 1H, H_4_), 4.94 (br s, 1H, H_3_), 5.10 (s, 2H, OCH_2_Ar), 6.54 (s, 2H, H_2′_ H_6′_), 7.02–7.05 (m, H_3′_’ H_5′_’), 7.28–7.45 (m, 7H, H_2′_’ H_6′_’ H_2′_’’ H_3′_’’ H_4′_’’ H_5′_’’ H_6′_’’). ^13^C NMR (100 MHz, CDCl_3_): δ 55.60, 60.52 (OCH_3_), 62.71 (C_3_), 65.66 (C_4_), 69.64 (-OCH_2_Ar), 94.79 (C_2′_, C_6′_), 115.32 (C_3′_’, C_5′_’), 126.62 (C_4′_), 126.99 (C_2′_’, C_6′_’), 127.12 (C_2′_’’, C_6′_’’), 127.74 (C_4′_’’), 128.22 (C_3′_’’, C_5′_’’), 132.50 (C_1′_’), 134.62 (C_1′_), 135.93 (C_1′_’’), 153.08 (C_3′_, C_5′_), 159.17 (C_4′_’), 160.27 (C_2_). HRMS: found 476.1233 [M + Na]^+^; C_25_H_24_^35^ClNO_5_Na requires 476.1241.

##### 3-Chloro-1-(3,4,5-trimethoxyphenyl)-4-(naphthalen-1-yl)azetidin-2-one (**10j**) 

Compound **10j** was prepared as described in the general method II above from imine **9j** and chloroacetyl chloride; yield: 12%, 244 mg, brown oil (HPLC: 95.5%). IR (NaCl, film) V_max_: 2604, 2498, 1763 (C=O, β-lactam), 1597, 1506, 1235, 1126 cm^−1^. ^1^H NMR (400 MHz, CDCl_3_): δ 3.71 (s, 6H, OCH_3_), 3.82 (s, 3H, OCH_3_), 4.64 (d, *J* = 1.52 Hz, 1H, H_4_), 5.81 (br s, 1H, H_3_), 6.65 (s, 2H, H_2′_ H_6′_), 7.40–8.21 (m, 7H, H_2′_’ H_3′_’ H_4′_’ H_5′_’ H_6′_’ H_7′_’ H_8′_’). ^13^C NMR (100 MHz, CDCl_3_): δ 55.74, 60.56 (OCH_3_), 62.35 (C_3_), 63.01 (C_4_), 94.94 (C_2′_, C_6′_), 122.22, 122.85, 124.89, 126.09, 126.91, 128.83, 129.09, 129.87, 132.89, 133.48 (C_4′_), 133.62 (C_1′_), 134.81 (C_1′_’), 153.25 (C_3′_, C_5′_), 160.24 (C_2_). HRMS: found 420.0992 [M + Na]^+^; C_22_H_20_^35^ClNO_4_Na requires 420.0979.

##### 3-Chloro-1-(3,4,5-trimethoxyphenyl)-4-(naphthalen-2-yl)azetidin-2-one (**10k**) 

Compound **10k** was prepared as described in the general method II above from imine **9k** and chloroacetyl chloride; yield: 9%, 185 mg, brown oil. IR (NaCl, film) V_max_: 2981, 2676, 1764 (C=O, β-lactam), 1621, 1507, 1235, 1127 cm^−1^. ^1^H NMR (400 MHz, CDCl_3_): δ 3.68 (s, 6H, OCH_3_), 3.77 (s, 3H, OCH_3_), 4.73 (d, *J* = 1.52 Hz, 1H, H_4_), 5.17 (d, *J* = 1.48 Hz, 1H, H_3_), 6.60 (s, 2H, H_2′_ H_6′_), 7.45–7.95 (m, 7H, H_2′_’ H_3′_’ H_4′_’ H_5′_’ H_6′_’ H_7′_’ H_8′_’). ^13^C NMR (100 MHz, CDCl_3_): δ 55.61, 60.51 (OCH_3_), 62.66 (C_3_), 66.13 (C_4_), 94.78 (C_2′_, C_6′_), 122.21, 125.60, 126.61, 127.48, 127.53, 129.32, 131.91, 132.56, 132.76 (C_4′_), 133.25 (C_1′_’), 134.72 (C_1′_), 153.14 (C_3′_, C_5′_), 160.21 (C_2_). HRMS: found 420.0990 [M + Na]^+^; C_22_H_20_^35^ClNO_4_Na requires 420.0979.

##### 3-Chloro-4-(4-methoxy-3-nitrophenyl)-1-(3,4,5-trimethoxyphenyl) azetidin-2-one (**10m**) 

Compound **10m** was prepared as described in the general method II above from imine **11n** and chloroacetyl chloride; yield: 10%, 140 mg, yellow solid, Mp 93–95 °C. IR (KBr) V_max_: 2944, 2667, 1764 (C=O, β-lactam), 1614, 1533, 1506, 1236, 1127 cm^−1^. ^1^H NMR (400 MHz, CDCl_3_): δ 3.76 (s, 6H, OCH_3_), 3.79 (s, 3H, OCH_3_), 4.00 (s, 3H, OCH_3_), 4.64 (d, *J* = 2.00 Hz, 1H, H_4_), 5.01 (d, *J* = 1.48 Hz, 1H, H_3_), 6.51 (s, 2H, H_2′_ H_6′_), 7.16–7.19 (m, 1H, H_2′_’), 7.55–7.58 (m, 1H, H_6′_’), 7.93 (d, *J* = 2.00 Hz, 1H, H_5′_’). ^13^C NMR (100 MHz, CDCl_3_): δ 55.71, 55.75, 60.53 (OCH_3_), 62.47 (C_3_), 64.37 (C_4_), 94.37 (C_2′_, C_6′_), 114.27 (C_5′_’), 123.41 (C_2′_’), 126.78 (C_4′_), 130.93 (C_6′_’), 131.88 (C_1′_), 132.25 (C_1′_’), 134.87 (C_3′_’), 152.95 (C_3′_, C_5′_), 153.32 (C_4′_’), 163.30 (C_2_). HRMS: found 445.0777 [M + Na]^+^; C_19_H_19_^35^ClN_2_O_7_Na requires 445.0778.

##### 3-Chloro-4-(3-hydroxy-4-methoxy-phenyl)-1-(3,4,5-trimethoxyphenyl)azetidin-2-one (**10n**)

A solution of the chloroacetic acid (1 mmol) and triphosgene (0.5 mmol) in dry DCM (10 mL) was refluxed under N_2_. After 30 min, triethylamine (1.5 mmol, 0.21 mL) was added, followed by the dropwise addition of the imine **9m** (0.5 mmol) dissolved in dry DCM (15 mL) over 30 min. The reaction mixture was heated at reflux for a further 6 h and then cooled, washed with water (20 mL) and saturated NaHCO_3_ (2 × 20 mL) and brine (10 mL). The solution was then dried over anhydrous sodium sulfate and the solvent was removed under reduced pressure. The product **10l** was isolated using flash column chromatography over silica gel. To a stirring solution of the protected β-lactam product **10l** (5 mmol), under N_2_ and 0 °C in dry conditions, THF was added dropwise in 1.5 equivalents of a 1.0 M *tert*-butylammonium fluoride (*t*-BAF) solution in hexanes (5 mmol). The resulting solution was stirred at 0 °C until the reaction was completed by TLC, then diluted with ethyl acetate (75 mL), washed with 0.1 M HCl (100 mL) and extracted with ethyl acetate (2 × 25 mL). All organic layers were washed with H_2_O (100 mL), and saturated brine (100 mL), then dried over anhydrous sodium sulphate. The solvent was removed under reduced pressure to yield the product that was purified using flash chromatography over silica gel (eluent, 4:1 *n*-hexane: ethyl acetate). Yield: 34%, 668 mg, brown oil. IR (NaCl film V_max_): 1770 cm^−1^(C=O), 3417 cm^−1^(OH). ^1^H NMR (400 MHz, CDCl_3_): δ 3.74 (s, 6H, OCH_3_), 3.78 (s, 3H, OCH_3_), 3.92 (s, 3H, OCH_3_), 4.61 (d, 1H, H_4_), 4.89 (d, *J*_3,4_ = 1.52 Hz, 1H, H_3_), 5.81 (s, 1H, OH), 6.56 (s, 2H, H_2′_, H_6′_), 6.87–6.95 (m, 3H, H_2′_’, H_5′_’, H_6′_’). ^13^C NMR (100 MHz, CDCl_3_): δ 55.59, 55.63, 60.51 (OCH_3_), 62.67 (C_3_), 65.63 (C_4_), 94.81 (C_2′_, C_6′_), 110.61 (C_2′_’), 111.58 (C_5′_’), 117.72 (C_6′_’), 127.52 (C_4′_), 132.50 (C_1′_), 134.63 (C_1′_’), 146.04 (C_3′_’), 147.00 (C_4′_’), 153.08 (C_3′_, C_5′_), 160.22 (C_2_). HRMS: found 416.0897 [M + Na]^+^; C_19_H_21_ClNO_6_Na requires 416.0877. 

##### 3,3-Dichloro-1-(3,4,5-trimethoxyphenyl)-4-phenylazetidin-2-one (**11a**) 

Compound **11a** was prepared as described in the general method II above from imine **9a** and dichloroacetyl chloride; yield: 31%, 597 mg, yellow solid, Mp 80–81 °C (HPLC: 100.0%). IR (KBr) V_max_: 2943, 2605, 2498, 1780 (C=O, β-lactam), 1594, 1506, 1239, 1127 cm^−1^. ^1^H NMR (400 MHz, CDCl_3_): δ 3.72 (s, 6H, OCH_3_), 3.80 (s, 3H, OCH_3_), 5.50 (s, 1H, H_4_), 6.55 (s, 2H, H_2′_ H_6′_), 7.35–7.47 (m, 5H, H_2′_’ H_3′_’ H_4′_’ H_5′_’ H_6′_’). ^13^C NMR (100 MHz, CDCl_3_): δ 55.66, 60.53 (OCH_3_), 73.83 (C_4_), 83.45 (C_3_), 95.35 (C_2′_, C_6′_), 127.37 (C_2′_’, C_6′_’), 128.53 (C_3′_’, C_5′_’), 129.61 (C_4′_’), 131.07 (C_4′_), 131.40 (C_1′_), 135.22 (C_1′_’), 153.19 (C_3′_, C_5′_), 157.82 (C_2_). HRMS: found 404.0433 [M + Na]^+^; C_18_H_17_^35^Cl_2_NO_4_Na requires 404.0432.

##### 3,3-Dichloro-4-(4-chlorophenyl)-1-(3,4,5-trimethoxyphenyl)azetidin-2-one (**11b**) 

Compound **11b** was prepared as described in the general method II above from imine **9b** and dichloroacetyl chloride; yield: 31%, 635 mg, brown solid, Mp 150–153 °C (HPLC: 99.7%). IR (KBr) V_max_: 2942, 2605, 2498, 1781 (C=O, β-lactam), 1594, 1506, 1238, 1123 cm^−1^. ^1^H NMR (400 MHz, CDCl_3_): δ 3.74 (s, 6H, OCH_3_), 3.81 (s, 3H, OCH_3_), 5.48 (s, 1H, H_4_), 6.53 (s, 2H, H_2′_ H_6′_), 7.30 (d, *J* = 8.56 Hz, 2H, H_3′_’ H_5′_’), 7.45 (d, *J* = 8.52 Hz, 2H, H_2′_’ H_6′_’). ^13^C NMR (100 MHz, CDCl_3_): δ 55.74, 60.55 (OCH_3_), 73.08 (C_4_), 83.31 (C_3_), 95.30 (C_2′_, C_6′_), 128.64 (C_2′_’, C_6′_’), 128.90 (C_3′_’, C_5′_’), 129.69 (C_4′_’), 131.14 (C_4′_), 135.42 (C_1′_), 135.67 (C_1′_’), 153.29 (C_3′_, C_5′_), 157.58 (C_2_). HRMS: found 438.0049 [M + Na]^+^; C_18_H_16_^35^Cl_3_NO_4_Na requires 438.0043.

##### 4-(4-Bromophenyl)-3,3-dichloro-1-(3,4,5-trimethoxyphenyl)azetidin-2-one (**11c**) 

Compound **11c** was prepared as described in the general method II above from imine **9c** and dichloroacetyl chloride; yield: 39%, 872 mg, yellow solid, Mp 150–152 °C (HPLC: 98.7%). IR (KBr) V_max_: 2978, 2605, 2498, 1782 (C=O, β-lactam), 1592, 1506, 1240, 1128 cm^−1^. ^1^H NMR (400 MHz, CDCl_3_): δ 3.75 (s, 6H, OCH_3_), 3.81 (s, 3H, OCH_3_), 5.47 (s, 1H, H_4_), 6.53 (s, 2H, H_2′_ H_6′_), 7.24 (d, *J* = 8.00 Hz, 2H, H_2′_’ H_6′_’), 7.61 (d, *J* = 7.52 Hz, 2H, H_3′_’ H_5′_’). ^13^C NMR (100 MHz, CDCl_3_): δ 55.75, 60.56 (OCH_3_), 73.13 (C_4_), 83.20 (C_3_), 95.29 (C_2′_, C_6′_), 123.88 (C_4′_’), 128.88 (C_2′_’, C_6′_’), 130.21 (C_4′_), 131.13 (C_1′_), 131.85 (C_3′_’, C_5′_’), 135.42 (C_1′_’), 153.29 (C_3′_, C_5′_), 157.57 (C_2_). HRMS: found 481.9561 [M + Na]^+^; C_18_H_16_^80^Br^35^Cl_2_NO_4_Na requires 481.9537.

##### 3,3-Dichloro-1-(3,4,5-trimethoxyphenyl)-4-(4-nitrophenyl)azetidin-2-one (**11d**) 

Compound **11d** was prepared as described in the general method II above from imine **9d** and dichloroacetyl chloride; yield: 44%, 932 mg, yellow solid, Mp 116–118 °C (HPLC: 95.5%). IR (KBr) V_max_: 2944, 2605, 2498, 1784 (C=O, β-lactam), 1596, 1506, 1346, 1127 cm^−1^. ^1^H NMR (400 MHz, CDCl_3_): δ 3.75 (s, 6H, OCH_3_), 3.81 (s, 3H, OCH_3_), 5.62 (s, 1H, H_4_), 6.51 (s, 2H, H_2′_ H_6′_), 7.56 (d, *J* = 7.52 Hz, 2H, H_2′_’ H_6′_’), 8.34 (d, *J* = 8.52 Hz, 2H, H_3′_’ H_5′_’). ^13^C NMR (100 MHz, CDCl_3_): δ 55.80, 60.55 (OCH_3_), 72.52 (C_4_), 83.02 (C_3_), 95.26 (C_2′_, C_6′_), 123.81 (C_2′_’, C_6′_’), 128.29 (C_3′_’, C_5′_’), 130.80 (C_4′_), 135.74 (C_1′_), 138.21 (C_4′_’), 148.42 (C_1′_’), 153.45 (C_3′_, C_5′_), 157.10 (C_2_). HRMS: found 449.0272 [M + Na]^+^; C_18_H_16_^35^Cl_2_N_2_O_6_Na requires 449.0283.

##### 3,3-Dichloro-1-(3,4,5-trimethoxyphenyl)-4-(4-phenoxyphenyl)azetidin-2-one (**11g**) 

Compound **11g** was prepared as described in the general method II above from imine **9g** and dichloroacetyl chloride; yield: 24%, 576 mg, grey solid (HPLC: 99.8%). Mp. 82–84 °C. IR (KBr) V_max_: 2942, 1779 (C=O, β-lactam), 1697, 1588, 1506, 1230, 1128 cm^−1^. ^1^H NMR (400 MHz, CDCl_3_): δ 3.75 (s, 6H, OCH_3_), 3.81 (s, 3H, OCH_3_), 5.48 (s, 1H, H_4_), 6.56 (s, 2H, H_2′_ H_6′_), 7.05–7.07 (m, 4H, H_2′_’ H_6′_’ H_2′_’’ H_6′_’’), 7.16–7.20 (m, 1H, H_4′_’’), 7.30–7.41 (m, 4H, H_3′_’ H_5′_’ H_3′_’’ H_5′_’’). ^13^C NMR (100 MHz, CDCl_3_): δ 55.71, 60.55 (OCH_3_), 73.44 (C_4_), 83.64 (C_3_), 95.44 (C_2′_, C_6′_), 117.99 (C_2′_’’, C_6′_’’), 119.20 (C_3′_’, C_5′_’), 123.77 (C_4′_’’), 125.23 (C_4′_), 128.97 (C_2′_’, C_6′_’), 129.53 (C_3′_’’, C_5′_’’), 131.33 (C_1′_), 135.30 (C_1′_’), 153.22 (C_3′_, C_5′_), 155.55 (C_4′_’), 157.84 (C_1′_’’), 158.63 (C_2_). HRMS: found 496.0679 [M + Na]^+^; C_24_H_21_^35^Cl_2_NO_5_Na requires 496.0694.

##### 4-(4-Benzyloxyphenyl)-3,3-dichloro-1-(3,4,5-trimethoxyphenyl) azetidin-2-one (**11h**) 

Compound **11h** was prepared as described in the general method II above from imine **9h** and dichloroacetyl chloride; yield: 26%, 626 mg, yellow oil. IR (NaCl, film) V_max_: 2605, 2498, 1778 (C=O, β-lactam), 1599, 1503, 1241, 1128 cm^−1^. ^1^H NMR (400 MHz, CDCl_3_): δ 3.73 (s, 6H, OCH_3_), 3.81 (s, 3H, OCH_3_), 5.11 (s, 2H, OCH_2_Ar), 5.46 (s, 1H, H_4_), 6.56 (s, 2H, H_2′_ H_6′_), 7.06 (d, *J* = 7.52 Hz, 2H, H_3′_’ H_5′_’), 7.30 (d, *J* = 7.52 Hz, 2H, H_2′_’ H_6′_’), 7.36–7.44 (m, 5H, H_2′_’’ H_3′_’’ H_4′_’’ H_5′_’’ H_6′_’’). ^13^C NMR (100 MHz, CDCl_3_): δ 55.69, 60.53 (OCH_3_), 69.65 (OCH_2_Ar), 73.60 (C_4_), 83.78 (C_3_), 95.41 (C_2′_, C_6′_), 114.78 (C_3′_’, C_5′_’), 127.04, 127.11 (C_2′_’, C_6′_’), 127.77 (C_4′_’’), 127.73, 127.77 (C_2′_’’, C_6′_’’), 128.21, 128.85 (C_3′_’’, C_5′_’’), 131.44 (C_4′_), 135.19 (C_1′_’), 135.90 (C_1′_), 142.69 (C_1′_’’), 153.17 (C_3′_, C_5′_), 157.94 (C_4′_’), 159.55 (C_2_). HRMS: found 488.1026 [M + H]^+^; C_25_H_24_^35^Cl_2_NO_5_, requires 488.1032.

##### 3,3-Dichloro-1-(3,4,5-trimethoxyphenyl)-4-(naphthalen-1-yl)azetidin-2-one (**11j**) 

Compound **11j** was prepared as described in the general method II above from imine **9j** and dichloroacetyl chloride; yield: 32%, 691 mg, yellow solid, Mp. 146–148 °C (HPLC: 98.4%). IR (KBr) V_max_: 2949, 1780 (C=O, β-lactam), 2593, 1505, 1235, 1127 cm^−1^. ^1^H NMR (400 MHz, CDCl_3_): δ 3.73 (s, 6H, OCH_3_), 3.84 (s, 3H, OCH_3_), 6.25 (s, 1H, H_4_), 6.66 (s, 2H, H_2′_ H_6′_), 7.32–8.16 (m, 7H, H_2′_’ H_3′_’ H_4′_’ H_5′_’ H_6′_’ H_7′_’ H_8′_’). ^13^C NMR (100 MHz, CDCl_3_): δ 55.84, 60.58 (OCH_3_), 71.19 (C_4_), 83.37 (C_3_), 95.53 (C_2′_, C_6′_), 122.40, 124.60, 124.64, 126.07, 126.98, 127.04, 128.83, 129.63, 130.72, 131.85 (C_4′_), 133.30 (C_1′_), 135.39 (C_1′_’), 153.32 (C_3′_, C_5′_), 157.99 (C_2_). HRMS: found 454.0605 [M + Na]^+^; C_22_H_19_^35^Cl_2_NO_4_Na requires 454.0589.

##### 3,3-Dichloro-1-(3,4,5-trimethoxyphenyl)-4-(naphthalen-2-yl)azetidin-2-one (**11k**) 

Compound **11k** was prepared as described in the general method II above from imine **9k** and dichloroacetyl chloride; yield: 35%, 764 mg, yellow solid, Mp. 164–166 °C (HPLC: 96.6%). IR (KBr) V_max_: 2983, 2693, 1779 (C=O, β-lactam), 1694, 1594, 1506, 1239, 1126 cm^−1^. ^1^H NMR (400 MHz, CDCl_3_): δ 3.70 (s, 6H, OCH_3_), 3.80 (s, 3H, OCH_3_), 5.68 (s, 1H, H_4_), 6.61 (s, 2H, H_2′_ H_6′_), 7.41–7.59 (m, 3H, H_2′_’ H_5′_’ H_6′_’), 7.89–7.95 (m, 4H, H_3′_’ H_4′_’ H_7′_’ H_8′_’). ^13^C NMR (100 MHz, CDCl_3_): δ 55.70, 60.53 (OCH_3_), 74.00 (C_4_), 83.56 (C_3_), 95.38 (C_2′_, C_6′_), 123.82, 126.47, 126.82, 127.35, 127.49, 127.79, 128.51, 128.68, 131.52, 132.49 (C_4′_), 133.48 (C_1′_’), 135.30 (C_1′_), 153.25 (C_3′_, C_5′_), 157.91 (C_2_). HRMS: found 454.0588 [M + Na]^+^; C_22_H_19_^35^Cl_2_NO_4_Na requires 454.0589.

##### 3,3-Dichloro-4-(4-methoxy-3-nitrophenyl)-1-(3,4,5-trimethoxyphenyl) azetidin-2-one (**11m**) 

Compound **11m** was prepared as described in the general method II above from imine **9n** and dichloroacetyl chloride; yield: 17%, 391 mg, colourless solid, Mp. 117–119 °C. IR (KBr) V_max_: 2943, 1781 (C=O, β-lactam), 1614, 1597, 1505, 1238, 1126 cm^−1^. ^1^H NMR (400 MHz, CDCl_3_): δ 3.77 (s, 6H, OCH_3_), 3.81 (s, 3H, OCH_3_), 4.03 (s, 3H, OCH_3_), 5.50 (s, 1H, H_4_), 6.53 (s, 2H, H_2′_ H_6′_), 7.19 (d, *J* = 9.04 Hz, 1H, H_2′_’), 7.49–7.52 (m, 1H, H_6′_’), 7.92 (d, *J* = 1.48 Hz, 1H, H_5′_’). ^13^C NMR (100 MHz, CDCl_3_): δ 55.85, 56.32, 60.55 (OCH_3_), 72.28 (C_4_), 83.44 (C_3_), 95.38 (C_2′_, C_6′_), 113.71 (C_5′_’), 123.53 (C_4′_), 124.97 (C_2′_’), 130.82 (C_1′_), 132.70 (C_6′_’), 135.68 (C_1′_’), 139.12 (C_3′_’), 153.41 (C_3′_, C_5′_), 153.62 (C_4′_’), 157.39 (C_2_). HRMS: found 479.0367 [M + Na]^+^; C_19_H_18_^35^Cl_2_N_2_O_7_Na requires 479.0389. 

##### 3,3-Dichloro-4-(3-hydroxy-4-methoxyphenyl)-1-(3,4,5-trimethoxyphenyl)azetidin-2-one (**11n**) 

(i) To a stirring, refluxing solution of the protected TBDMS imine **9m** (5 mmol) and triethylamine (6 mmol) in anhydrous dichloromethane (40 mL), a solution of the dichloroacetyl chloride (6 mmol) in anhydrous dichloromethane (10 mL) was added over 45 min under nitrogen. The reaction heated at reflux during the 5 h and retained at room temperature for 16 h under nitrogen, until the starting material had disappeared as monitored by TLC (eluent, 50:50 *n*-hexane: ethyl acetate). The reaction mixture was washed with water (2 × 100 mL). The organic layer was dried over anhydrous Na_2_SO_4_, and the solvent was then removed under reduced pressure. The crude product was purified using flash chromatography over silica gel (eluent: *n*-hexane: ethyl acetate, 4:1) to afford the product **11l** as an oil that was used immediately in the following reaction. (ii) To a stirring solution of the protected β-lactam **11l** (5 mmol), under N_2_ and 0 °C in dry conditions, THF was added dropwise in 1.5 equivalents of a 1.0 M *tert*-butylammonium fluoride (*t*-BAF) solution in hexanes (5 mmol). The resulting solution was stirred at 0 °C until reaction was complete, as verified by TLC. The reaction mixture was diluted with ethyl acetate (75 mL) and washed with 0.1 M HCl (100 mL). The aqueous layer was further extracted with ethyl acetate (2 × 25 mL). All organic layers were collected and washed with water (100 mL), and saturated brine (100 mL) and dried over an anhydrous sodium sulphate. The solvent was removed under reduced pressure to yield the product that was purified using flash chromatography over silica gel (eluent, 4:1 *n*-hexane: ethyl acetate). Yield: 28%, 600 mg, colourless oil. IR (NaCl, film) V_max_: 3396 (OH), 1782 (C=O, β-lactam), 1595, 1508, 1234, 1123 cm^−1^. ^1^H NMR (400 MHz, CDCl_3_): δ 3.74 (s, 6H, OCH_3_), 3.79 (s, 3H, OCH_3_), 3.93 (s, 3H, OCH_3_), 5.39 (s, 1H, H_4_), 6.56 (s, 2H, H_2′_ H_6′_), 6.85–6.91 (m, 3H, H_2′_’ H_5′_’ H_6′_’). ^13^C NMR (100 MHz, CDCl_3_): δ 55.51, 55.70, 60.52 (OCH_3_), 73.54 (C_4_), 83.68 (C_3_), 95.42 (C_2′_, C_6′_), 110.19 (C_5′_’), 113.35 (C_2′_’), 119.43 (C_6′_’), 120.54 (C_4′_), 123.99 (C_1′_), 131.45 (C_1′_’), 145.50 (C_3′_’), 147.33 (C_4′_’), 153.16 (C_3′_, C_5′_), 157.91 (C_2_). HRMS: found 428.0653 [M + H]^+^; calculated for C_19_H_20_Cl_2_NO_6_, 428.0668. 

##### 3-Chloro-1-(4-methoxyphenyl)-4-(3,4,5-trimethoxyphenyl)azetidin-2-one (**12a**)

Compound **12a** was synthesised using the general method II above from imine **9t** and chloroacetyl chloride to afford the product as an orange powder; yield: 59%, Mp. 101–102 °C (HPLC: 96%). IR (NaCl, film) V_max_: 2604, 2498, 1758 (C=O, β-lactam), 1598, 1503, 1246, 1126 cm^−1^. ^1^H NMR (400 MHz, CDCl_3_): δ 3.78 (s, 3H, OCH_3_), 3.84 (s, 6H, OCH_3_), 3.86 (s, 3H, OCH_3_), 4.63 (d, *J* = 1.48 Hz, 1H, H_4_), 4.90 (d, *J* = 2.00 Hz, 1H, H_3_), 6.56 (s, 2H, H_2′_’ H_6′_’), 6.83 (d, *J* = 9.04 Hz, 2H, H_3′_ H_5′_), 7.27 (d, *J* = 9.04 Hz, 2H, H_2′_ H_6′_). ^13^C NMR (100 MHz, CDCl_3_): δ 54.99, 55.81, 60.44 (OCH_3_), 62.80 (C_3_), 65.97 (C_4_), 102.31 (C_2′_’, C_6′_’), 113.98 (C_3′_, C_5′_), 118.46 (C_2′_, C_6′_), 129.79 (C_1′_), 130.12 (C_4′_’), 138.17 (C_1′_’), 153.64 (C_3′_’, C_5′_’), 156.29 (C_4′_), 159.81 (C_2_). HRMS: found 400.0928 [M + Na]^+^; C_19_H_20_^35^ClNO_5_Na requires 400.0928.

##### 3-Chloro-1-(4-ethoxyphenyl)-4-(3,4,5-trimethoxyphenyl)azetidin-2-one (**12b**)

Compound **12b** was synthesised using the general method II above from imine **9u** and chloroacetyl chloride to afford the product as a creamy powder; yield: 45%, Mp: 113–115 °C (HPLC: 84%), IR V_max_: (ATR): 1759.5 (C=O) cm^−1^. ^1^H NMR (400 MHz, CDCl_3_): δ 1.33–1.38 (m, 3H, OCH_2_CH_3_), 3.80 (s, 6H, OCH_3_), 3.83 (s, 3H, OCH_3_), 3.92 (d, *J* = 3.66 Hz, 2H, OCH_2_CH_3_), 4.59 (d, *J* = 1.83 Hz, 1H, H_4_), 4.85 (d, *J* = 1.83 Hz, 1H, H_3_), 6.52 (s, 2H, ArH), 6.78 (d, *J* = 9.16 Hz, 2H, ArH), 7.22 (d, *J* = 8.54 Hz, 2H, ArH). ^13^C NMR (100 MHz, CDCl_3_): δ 14.73, 56.24, 60.85, 63.24, 66.39, 102.81, 106.70, 114.97, 118.88, 121.97, 130.57, 154.07, 156.13, 160.17 (C_2_, C=O). HRMS: found 414.1080 [M + Na]^+^; C_20_H_22_^35^ClNNaO_5_ requires 414.1084. 

##### 3-Chloro-1-(4-(methylthio)phenyl)-4-(3,4,5-trimethoxyphenyl) azetidin-2-one (**12c**)

Compound **12c** was synthesised using the general method II above from imine **19v** and chloroacetyl chloride to afford the product as orange powder; yield: 50%, Mp: 101–103 °C. IR V_max_: (ATR): 1760.6 (C=O) cm^−1^. ^1^H NMR (400 MHz, CDCl_3_): δ 2.42 (s, 3H, SCH_3_), 3.80 (s, 6H, OCH_3_), 3.83 (s, 3H, OCH_3_), 4.60 (d, *J* = 1.83 Hz, 1 H, H4), 4.87 (d, *J* = 2.44 Hz, 1H, H3), 6.52 (s, 2H, ArH), 7.13–7.17 (m, 2H, ArH), 7.20–7.24 (m, 2H, ArH). ^13^C NMR (100 MHz, CDCl_3_): δ 16.17, 56.27, 60.85, 63.22, 66.39, 102.77, 106.71, 118.03, 120.68, 127.59, 130.31, 134.89, 154.13, 160.53 (C_2_, C=O). HRMS: found 416.0701 [M + Na]^+^; C_19_H_20_^35^ClNNaO_4_S requires 416.0699. 

##### 3,3-Dichloro-4-(3,4,5-trimethoxyphenyl)-1-(4-methoxyphenyl)azetidin-2-one (**13a**)

Compound **13a** was prepared as described in the general method II above from imine **9t** and dichloroacetyl chloride; yield: 18%, 379 mg, yellow solid, Mp. 64–66 °C (HPLC: 100%). IR (KBr) V_max_: 2605, 2498, 1778 (C=O, β-lactam), 1691, 1592, 1508, 1250, 1127 cm^−1^. ^1^H NMR (400 MHz, CDCl_3_): δ 3.79 (s, 3H, OCH_3_), 3.83 (s, 6H, OCH_3_), 3.89 (s, 3H, OCH_3_), 5.39 (s, 1H, H_4_), 6.51 (s, 2H, H_2′_’ H_6′_’), 6.86 (d, *J* = 8.00 Hz, 2H, H_3′_ H_5′_), 7.29 (d, *J* = 8.04 Hz, 2H, H_2′_ H_6′_). ^13^C NMR (100 MHz, CDCl_3_): δ 55.02, 55.81, 60.48 (OCH_3_), 73.86 (C_4_), 83.70 (C_3_), 104.26 (C_2′_’, C_6′_’), 114.10 (C_3′_, C_5′_), 118.98 (C_2′_, C_6′_), 126.59 (C_1′_), 128.69 (C_4′_’), 138.56 (C_1′_’), 153.18 (C_3′_’, C_5′_’), 156.77 (C_4′_), 157.51 (C_2_). HRMS: found 434.0535 [M + Na]^+^; C_19_H_19_^35^Cl_2_NO_5_Na requires 434.0538. 

##### 3,3-Dichloro-1-(4-ethoxyphenyl)-4-(3,4,5-trimethoxyphenyl)azetidin-2-one (**13b**)

Compound **13b** was synthesised using the general method II above from imine **9u** and dichloroacetyl chloride to afford the product as a brown powder; yield: 25%, Mp: 133–134 °C. IR V_max_: (ATR): 1774.5 (C=O) cm^−1^. ^1^H NMR (400 MHz, CDCl_3_): δ 1.37 (t, *J* = 7.02 Hz, 3 H, OCH_2_CH_3_), 3.79 (s, 6 H, OCH_3_), 3.86 (s, 3 H, OCH_3_), 3.97 (q, *J* = 6.71 Hz, 2 H, OCH_2_CH_3_), 5.34 (s, 1 H, H4), 6.47 (s, 2 H, ArH), 6.77–6.84 (m, 2 H, ArH), 7.23–7.25 (m, 2 H, ArH). ^13^C NMR (100 MHz, CDCl_3_): δ 14.29, 55.44, 56.25, 60.88, 74.31, 81.62, 104.77, 114.54, 119.39, 127.00, 129.15, 153.61, 157.22, 159.65 (C_2_, C=O). HRMS: found 448.0676 [M + Na]^+^; C_20_H_21_^35^Cl_2_NNaO_5_ requires 448.0694. 

##### 3,3-Dichloro-1-(4-(methylthio)phenyl)-4-(3,4,5-trimethoxyphenyl) azetidin-2-one (**13c**)

Compound **13c** was synthesised using the general method II above from imine **9v** and dichloroacetyl chloride to afford the product as brown solid; yield: 47%, Mp: 117–119 °C (HPLC: 98%), IR V_max_: (ATR): 1771.9 (C=O) cm^−1^. ^1^H NMR (400 MHz, CDCl_3_): δ 2.43 (s, 3H, SCH_3_), 3.79 (s, 6H, OCH_3_), 3.86 (s, 3H, OCH_3_), 5.36 (s, 1H, H4), 6.47 (s, 2H, ArH), 7.18 (s, 2H, ArH), 7.23 (s, 2H, ArH). ^13^C NMR (100 MHz, CDCl_3_): δ 15.98, 56.28, 60.89, 74.28, 83.63, 104.73, 118.51, 126.77, 127.41, 133.11, 135.91, 139.17, 153.67, 158.19 (C_2_, C=O). HRMS: found 450.0320 [M + Na]^+^; C_19_H_19_^35^Cl_2_NNaO_4_S requires 450.0310. 

##### 3-Chloro-1-(3,5-dimethoxyphenyl)-4-(4-methoxyphenyl)azetidin-2-one (**14a**)

Compound **14a** was synthesised using the general method II above from imine **9w** and chloroacetyl chloride to afford the product as yellow powder; yield: 8%, Mp: 93–94 °C, IR V_max_ (ATR): 1751.3 (C=O) cm^−1^. ^1^H NMR (400 MHz, CDCl_3_): δ 3.65 (s, 6H, OCH_3_), 3.71 (s, 3H, OCH_3_), 4.45 (s, 1H, H4), 4.68 (s, 1H, H3), 6.11–6.20 (m, 1H, ArH), 6.43 (d, *J* = 1.83 Hz, 2H, ArH), 6.80 (d, *J* = 8.54 Hz, 2H, ArH), 7.28 (s, 2H, ArH). ^13^C NMR (100 MHz, CDCl_3_): δ 56.45, 60.67, 63.45, 66.36, 95.22, 115.56, 126.95, 127.88, 132.13, 135.10, 153.52, 158.77, 160.55 (C_2_, C=O). HRMS: calculated for C_18_H_19_^35^ClNO_4_ [M + H]^+^ 348.1003; found 348.1000. 

##### 3-Chloro-1-(3,5-dimethoxyphenyl)-4-(4-ethoxyphenyl)azetidin-2-one (**14b**)

Compound **14b** was synthesised using the general method II above from imine **9x** and chloroacetyl chloride to afford the product as an oil; yield: 20%, Mp: 129–130 °C, IR V_max_ (ATR): 1753.6 (C=O) cm^−1^. ^1^H NMR (400 MHz, CDCl_3_): δ 1.39 (t, *J* = 6.71 Hz, 3H, OCH_2_CH_3_), 3.68 (s, 6H, OCH_3_), 4.01 (q, *J* = 7.12 Hz, 2H, OCH_2_CH_3_), 4.55 (s, 1H, H4), 4.88 (s, 1H, H3), 6.13–6.23 (m, 1H, ArH), 6.45 (d, *J* = 1.83 Hz, 2H, ArH), 6.89 (d, *J* = 8.54 Hz, 2H, ArH), 7.27 (s, 2H, ArH). ^13^C NMR (100 MHz, CDCl_3_): δ 14.33, 56.45, 60.67, 63.45, 66.36, 95.22, 115.56, 126.95, 127.88, 132.13, 135.10, 153.52, 158.77, 160.55 (C_2_, C=O). HRMS: calculated for C_19_H_20_^35^ClNNaO_4_ [M + Na]^+^ 384.0979; found 384.0992. 

##### 3,3-Dichloro-1-(3,5-dimethoxyphenyl)-4-(4-methoxyphenyl)azetidin-2-one (**15a**)

Compound **15a** was synthesised using method II above from imine **9w** and dichloroacetyl chloride to afford the product as yellow oil; yield: 13% (HPLC: 95%), IR V_max_ (ATR): 1774.6 (C=O) cm^−1^. ^1^H NMR (400 MHz, CDCl_3_): δ 3.71 (s, 6H, OCH_3_), 3.82 (s, 3H, OCH_3_), 5.40 (s, 1H, H4), 6.24 (t, *J* = 2.14 Hz, 1H, ArH), 6.48 (d, *J* = 2.44 Hz, 2H, ArH), 6.93 (d, *J* = 9.16 Hz, 2H, ArH), 7.23 (d, *J* = 8.54 Hz, 2H, ArH). ^13^C NMR (100 MHz, CDCl_3_): δ 55.30, 55.43, 74.03, 83.43, 96.75, 97.64, 114.32, 123.41, 129.11, 137.46, 158.67, 160.75 (C_2_, C=O). HRMS: calculated for C_18_H_17_^35^Cl_2_NNaO_4_ [M + Na]^+^ 404.0432; found 404.0428. 

##### 3,3-Dichloro-1-(3,5-dimethoxyphenyl)-4-(4-ethoxyphenyl)azetidin-2-one (**15b**)

Compound **15b** was synthesised using method II above from imine **9x** and dichloroacetyl chloride to afford the product as a brown oil; yield: 7% (HPLC: 96%), IR V_max_ (ATR): 1772.4 (C=O) cm^−1^. ^1^H NMR (400 MHz, CDCl_3_): δ 1.40 (t, *J* = 6.71 Hz, 3H, OCH_2_CH_3_), 3.69 (s, 6H, OCH_3_), 4.02 (q, *J* = 7.32 Hz, 2H, OCH_2_CH_3_), 5.38 (s, 1H, H4), 6.20–6.25 (m, 1H, ArH), 6.47 (s, 2H, ArH), 6.90 (m, *J* = 8.55 Hz, 2H, ArH), 7.21 (m, *J* = 8.54 Hz, 2H, ArH). ^13^C NMR (100 MHz, CDCl_3_): δ 14.74, 55.41, 63.52, 74.06, 84.24, 96.74, 97.63, 114.75, 123.18, 129.08, 137.47, 158.67, 160.15 (C_2_, C=O). HRMS: calculated for C_19_H_19_^35^Cl_2_NNaO_4_ [M + Na]^+^ 418.0589; found 418.0574. 

##### 3-Bromo-4-(4-methoxyphenyl)-1-(3,4,5-trimethoxyphenyl)azetidin-2-one (**16a**)

Compound **16a** was synthesised from imine **9e** and bromoacetyl chloride using the general method II above and afford a product as a red oil; yield: 11% (HPLC: 100%). IR V_max_: (ATR): 1757.1 (C=O) cm^−1^. ^1^H NMR (400 MHz, CDCl_3_): δ 3.66 (s, 6 H, OCH_3_), 3.71 (s, 3 H, OCH_3_), 3.77 (s, 3 H, OCH_3_), 4.65 (d, *J* = 1.96 Hz, 1 H, H4), 5.05 (d, *J* = 1.96 Hz, 1 H, H3), 6.48 (s, 2 H, ArH), 6.89 (m, *J* = 8.71 Hz, 2H, ArH), 7.27 (m, *J* = 8.71 Hz, 2H, ArH). ^13^C NMR (100 MHz, CDCl_3_): δ 49.91, 55.33, 56.00, 60.51, 66.14, 95.34, 114.79, 127.54, 131.44, 132.85, 135.07, 153.46, 160.47, 170.54 (C_2_, C=O). HRMS: found 444.0452 [M + Na]^+^; C_19_H_20_^79^BrNaNO_5_ requires 444.0423.

##### 3-Bromo-4-(4-ethoxyphenyl)-1-(3,4,5-trimethoxyphenyl)azetidin-2-one (**16b**)

Compound **16b** was prepared from imine **9f** and bromoacetyl chloride using the general method II above and afforded the product as an oil; yield: 31% (HPLC: 95%). IR V_max_ (ATR): 1735.6 (C=O) cm^−1^. ^1^H NMR (400 MHz, CDCl_3_): δ 1.31 (t, *J* = 6.84 Hz, 3H, OCH_2_CH_3_), 3.70 (s, 3H, OCH_3_), 3.79 (s, 6H, OCH_3_), 4.02 (q, 2H, *J* = 6.61 Hz, OCH_2_CH_3_), 4.86 (d, *J* = 1.66 Hz, 1H, H_4_), 4.95 (d, *J* = 1.66 Hz, 1 H, H_3_), 6.44 (s, 2H, ArH), 6.84 (d, *J* = 8.71 Hz, 2H, ArH), 7.23 (d, *J* = 8.71 Hz, 2H, ArH). ^13^C NMR (100 MHz, CDCl_3_): δ 14.30, 49.78, 55.91, 60.59, 63.54, 66.17, 95.36, 115.24, 126.35, 127.55, 132.79, 135.39, 159.82, 161.10, 170.46 (C_2,_ C=O). HRMS: found 458.0550 M+H]^+^; C_20_H_22_^79^BrNaNO_5_ requires 458.0579.

##### 3-Bromo-4-(4-(methylthio)phenyl)-1-(3,4,5-trimethoxyphenyl)azetidin-2-one (**16c**)

Compound **16c** was prepared from imine **9i** and bromoacetyl chloride following the general method II above to afford the product as an oil; yield: 30% (HPLC: 100%). IR V_max_ (ATR): 1757.9 (C=O) cm^−1^. ^1^H NMR (400 MHz, CDCl_3_): δ 2.42 (s, 3H, SCH_3_), 3.65 (s, 3H, OCH_3_), 3.85 (s, 6H, OCH_3_), 4.56 (d, *J* = 1.66 Hz, 1H, H_4_), 4.90 (d, *J* = 1.66 Hz, 1H, H_3_), 6.46 (s, 2 H, ArH), 7.19–7.23 (m, 2 H, ArH), 7.24 (s, 2H, ArH). ^13^C NMR (100 MHz, CDCl_3_): δ 15.28, 49.70, 56.05, 60.88, 66.90, 95.36, 126.61, 127.34, 131.14, 132.70, 135.13, 140.76, 153.49, 170.39 (C_2,_ C=O). HRMS: found 460.0165 [M + Na]^+^; C_19_H_20_^79^BrNaNO_4_S requires 460.0194.

##### 3-Bromo-4-(4-(ethylthio)phenyl)-1-(3,4,5-trimethoxyphenyl)azetidin-2-one (**16d**)

Compound **16d** was prepared following the general method II above from imine **9o** and bromoacetyl chloride to afford the title product as a brown oil; yield: 22% (HPLC: 97%). IR V_max_ (ATR): 1760.1 (C=O) cm^−1^. ^1^H NMR (400 MHz, CDCl_3_): δ 1.10 (t, *J* = 7.46 Hz, 3H, SCH_2_CH_3_), 2.78 (q, *J* = 7.46 Hz, 2H, SCH_2_CH_3_), 3.61 (s, 3H, OCH_3_), 3.68 (s, 6H, OCH_3_), 4.03 (d, *J* = 1.66 Hz, 1H, H_4_), 4.89 (d, *J* = 1.66 Hz, 1H, H_3_), 6.43 (s, 2H, ArH), 7.17 (s, 2H, ArH), 7.76 (s, 2H, ArH). ^13^C NMR (100 MHz, CDCl_3_): δ 15.26, 26.86, 49.53, 55.98, 60.83, 62.74, 66.06, 95.39, 126.67, 128.74, 131.67, 132.64, 135.06, 139.16, 153.43, 171.04 (C_2,_ C=O). HRMS: found 474.0378 [M + Na]^+^; C_20_H_22_^79^BrNaNO_4_S requires 474.0351.

##### 3-Bromo-4-(4-methoxy-3-methylphenyl)-1-(3,4,5-trimethoxyphenyl) azetidin-2-one (**16e**)

Compound **16e** was prepared using the general procedure II above from imine **9p**, and bromoacetyl chloride to afford the product as an oil; yield: 29% (HPLC: 95%). IR V_max_ (ATR): 1758.6 (C=O) cm^−1^. ^1^H NMR (400 MHz, CDCl_3_): δ 2.12 (s, 3H, CH_3_), 3.62 (s, 3H, OCH_3_) 3.67 (s, 6H, OCH_3_), 3.73 (s, 3H, OCH_3_), 4.53 (d, *J* = 1.66 Hz, 1H, H4), 4.83 (d, *J* = 1.66 Hz, 1H, H3), 6.47 (s, 2H, ArH), 6.76 (s, 1H, ArH), 7.08 (s, 1H, ArH), 7.12 (s, 1H, ArH). ^13^C NMR (100 MHz, CDCl_3_): δ 15.42, 49.23, 56.31, 61.73, 68.49, 99.02, 115.72, 123.12, 125.50, 130.65, 134.41, 135.27, 136.86, 153.03, 156.78, 170.49 (C_2,_ C=O). HRMS: found 436.0764 [M + H]^+^; C_20_H_23_^79^BrNO_5_ requires 436.0760. 

##### 3-Bromo-4-(3-fluoro-4-methoxyphenyl)-1-(3,4,5-trimethoxyphenyl) azetidin-2-one (**16f**)

Compound **16f** was prepared using the general procedure II above from imine **9q**, and bromoacetyl chloride to afford the product as an oil; yield: 25%; (HPLC: 96%). IR V_max_ (ATR): 1760.2 (C=O) cm^−1^. ^1^H NMR (400 MHz, CDCl_3_): δ 3.77 (s, 3H, OCH_3_), 3.81 (s, 9H, OCH_3_), 4.56 (d, *J* = 1.66 Hz, 1H, H4), 4.87 (d, *J* = 1.66 Hz, 1H, H3), 6.47 (s, 2H, ArH), 7.14 (m, 3H, ArH). ^13^C NMR (100 MHz, CDCl_3_): δ 49.73, 55.56, 56.71, 60.88, 65.49, 95.29, 113.31, 114.15, 122.33, 127.52, 132.64, 135.25, 148.56, 151.46, 153.57, 171.32 (C_2,_ C=O). HRMS: found 462.0340 [M + H]^+^; C_19_H_19_^79^BrFNaNO_5_ requires 462.0328.

##### 3-Bromo-4-(3-chloro-4-methoxyphenyl)-1-(3,4,5-trimethoxyphenyl) azetidin-2-one (**16g**)

Compound **16g** was prepared using the general procedure II above from **9r** and bromoacetyl chloride to afford the product as yellow oil; yield: 29% (HPLC: 100%). IR V_max_ (ATR): 1759.6 (C=O) cm^−1^. ^1^H NMR (400 MHz, CDCl_3_): δ 3.71 (s, 3H, OCH_3_), 3.66 (s, 6H, OCH_3_), 3.84 (s, 3H, OCH_3_), 4.57 (d, *J* = 2.07 Hz, 1H, H_4_), 4.86 (d, *J* = 2.07 Hz, 1H, H_3_), 6.46 (s, 2H, ArH), 6.92 (s, 1H, ArH), 7.22 (s, 1H, ArH), 7.36 (s, 1H, ArH). ^13^C NMR (100 MHz, CDCl_3_): δ 49.73, 56.06, 56.23, 60.87, 65.34, 95.30, 112.61, 123.55, 125.68, 128.02, 132.63, 135.23, 136.55, 152.86, 156.27, 160.47 (C_2,_ C=O). HRMS: found 478.0016 [M + Na]^+^; C_19_H_19_^79^Br^35^ClNaNO_5_ requires 478.0033.

##### 3-Bromo-4-(3-bromo-4-methoxyphenyl)-1-(3,4,5-trimethoxyphenyl) azetidin-2-one (**16h**)

Compound **16h** was prepared using the general procedure II above from imine **9s**, and bromoacetyl chloride to afford the product as brown oil; yield: 20% (HPLC: 100%). IR V_max_ (ATR): 1758.9 (C=O) cm^−1^. ^1^H NMR (400 MHz, CDCl_3_): δ 3.78 (s, 3H, OCH_3_), 3.84 (s, 6H, OCH_3_), 4.05 (s, 3H, OCH_3_), 4.57 (d, *J* = 1.66 Hz, 1 H, H_4_), 4.86 (d, *J* = 1.66 Hz, 1H, H_3_), 6.46 (s, 2H, ArH), 6.88 (s, 1H, ArH), 6.84 (s, 1H, ArH), 7.54 (s, 1H, ArH). ^13^C NMR (100 MHz, CDCl_3_): δ 55.13, 56.23, 60.54, 66.43, 100.18, 112.36, 114.51, 126.04, 132.21, 134.78, 136.55, 137.60, 154.63, 156.32, 170.70 (C_2,_ C=O). HRMS: found 521.9500 [M + Na]^+^; C_19_H_19_^79^Br_2_NaNO_5_ requires 521.9528.

##### 3-Bromo-4-(3-(*tert*-butyldimethylsilyl)-4-methoxyphenyl)-1-(3,4,5-trimethoxyphenyl)azetidin-2-one (**16i**)

Compound **16i** was prepared using the general procedure II above from protected TBDMS imine **9m** (2 mmol) and bromoacetyl chloride (6 mmol). The product was isolated as a red oil; 50 mg, yield: 5%, HPLC: 98%. IR V_max_ (ATR): 1760.02cm^−1^ (C=O, β-lactam). ^1^H NMR (400 MHz, CDCl_3_) δ ppm 0.04 (s, 6 H, Si-CH_3_), 0.88 (s, 9 H, CH_3_), 3.65 (s, 3 H, OCH_3_), 3.71 (s, 6 H, OCH_3_), 4.57 (d, *J* = 2.07 Hz, 1 H H_4_), 4.84 (d, *J* = 2.07 Hz, 1 H, H_3_), 6.48 (s, 2 H, H_2′_, H_6′_), 6.77 (s, 1 H, H_5′_’), 6.84 (s, 1 H, H_6′_’), 6.92 (s, 1 H, H_2′_’). ^13^C NMR (101 MHz, CDCl_3_) δ ppm -1.5 (Si-CH_3_), 25.54 (CH_3_), 30.64 (Si-C-CH_3_), 49.81 (C_3_) 55.96, 60.88, 66.04 (OCH_3_), 95.33 (C_2′_, C_6′_), 112.41 (C_5′_’), 119.89 (C_3′_’), 126.98 (C_6′_’), 127.25 (C_2′_’), 132.79 (C_4′_), 132.98 (C_1′_’), 134.98 (C_1′_’), 153.43 (C_3′_, C_5′_), 160.91 (C_4′_’), 170.39 (C_2,_ C=O). HRMS: [M + H]^+^ calculated for C_25_H_35_BrNO_5_Si, 536.1468; found 536.1456.

##### 3-Bromo-4-(3-hydroxy-4-methoxyphenyl)-1-(3,4,5-trimethoxyphenyl) azetidin-2-one (**16j**)

Compound **16i** was deprotected with *tert*-butylammonium fluoride (*t*-BAF) using the method as described above for compound **10n** to afford the product as a red oil; 32%, 13 mg, HPLC: 100%. IR V_max_ (ATR): 3502.80, 1719.42 cm^−1^ (OH, C=O β-lactam). ^1^H NMR (400 MHz, CDCl_3_) δ ppm 3.70 (s, 3 H, OCH_3_), 3.89 (s, 9 H, OCH_3_), 4.58 (d, *J* = 2.07 Hz, 1 H, H_4_), 4.86 (d, *J* = 2.07 Hz, 1 H, H_3_), 6.27 (s, 1 H, OH ), 6.52 (s, 2 H, H_5′_’, H_6′_’), 6.87 (d, *J* = 4.98 Hz, 1 H, H_5′_’) 6.92 (d, *J* = 2.07 Hz, 1 H, H_6′_’) 6.96 (s, 1 H, H_2′_’). ^13^C NMR (100 MHz, CDCl_3_) δ ppm 49.67 (C_3_), 56.07, 60.46 (OCH_3_), 63.65 (C_4_), 95.36 (C_2′_, C_6′_), 111.09 (C_5′_’), 113.19 (C_2′_’), 118.14 (C_6′_’), 125.48 (C_4′_), 132.90 (C_1′_), 137.23 (C_1′_’), 146.48 (C_3′_’), 146.68 (C_4′_’), 153.51 (C_3′_, C_5′_), 176.45 (C_2,_ C=O). HRMS: [M + H]^+^ calculated for C_19_H_21_BrNO_6_, 438.0554; found 438.0546.

#### 3.1.4. 5-(3,3-Dichloro-1-(3,4,5-trimethoxyphenyl)-4-oxoazetidin-2-yl)-2-methoxyphenyl dibenzyl phosphate (**17**)

Carbon tetrachloride (85 mmol) was added to a solution of phenol **11n** (17 mmol) in acetonitrile (100 mL cooled to 0 °C). The resulting solution was stirred for 10 min, then diisopropylethylamine (35 mmol) and dimethylaminopyridine (1.7 mmol) were added, followed by a dropwise addition of dibenzyl phosphate (24.5 mmol). When the reaction was complete, 0.5 M KH_2_PO_4_ (aq) was added, and the mixture was allowed to warm to room temperature. An ethyl acetate extract (3×50 mL) was washed with saturated sodium chloride solution (100 mL) followed by water (100 mL) and the mixture was dried using anhydrous sodium sulfate. The solvent was reduced in vacuo and the product was isolated using flash column chromatography over silica gel (eluent, *n*-hexane: ethyl acetate gradient). Yield: 66%, 326 mg, brown oil. IR (NaCl, film) V_max_: 2945, 1748 (C=O, β-lactam), 1605, 1507, 1321 (P=O), 1237 cm^−1^. ^1^H NMR (400 MHz, CDCl_3_): δ 3.72 (s, 6H, OCH_3_), 3.78 (s, 3H, OCH_3_), 3.83 (s, 3H, OCH_3_), 5.12–5.18 (m, 4H (OCH_2_Ph)_2_), 5.36 (s, 1H, H_4_), 6.53 (s, 2H, H_2′_, H_6′_), 6.96–7.19 (m, 3H, H_2′_’, H_5′_’, H_6′_’), 7.31–7.36 (m, 10H, Ar-H). ^13^C NMR (100 MHz, CDCl_3_): δ 55.52, 55.72, 60.52 (OCH_3_), 69.57, 69.60, 69.65 (OPO(OCH_2_Ph)_2_), 73.04 (C_4_), 83.57 (C_3_), 95.36 (C_2′_, C_6′_), 112.27 (C_5′_’), 120.87 (C_2′_’), 124.82 (C_6′_’), 127.42, 127.53, 128.17, 128.20, 128.27, 131.27 (C_4′_), 134.93, 135.00, 135.27, 139.40, 151.48, 153.22 (C_3′_, C_5′_), 157.77 (C_2_). HRMS: found 710.1107 [M + Na]^+^; C_33_H_32_^35^Cl_2_NO_9_PNa requires 710.1089.

#### 3.1.5. 5-(3,3-Dichloro-1-(3,4,5-trimethoxyphenyl)-4-oxoazetidin-2-yl)-2-methoxyphenyl dihydrogen phosphate (**18**)

The dibenzylphosphate ester **17** (2 mmol) was dissolved in ethanol:ethyl acetate (50 mL; 1:1 mixture) and hydrogenated over 1.2 g of 10% palladium on carbon until reaction was complete, as monitored by TLC (approximately 3 h). The catalyst was filtered, the solvent was reduced in vacuo and the product isolated using flash column chromatography over silica gel (eluent, *n*-hexane: ethyl acetate gradient). Yield: 75%, 213 mg, brown oil. IR (NaCl, film) V_max_: 3429 (OH), 1778 (C=O, β-lactam), 1298 (P=O), 1240, 1128 cm^−1^. ^1^H NMR (400 MHz, CDCl_3_): δ 3.66 (s, 9H, OCH_3_), 3.73 (s, 3H, OCH_3_), 5.45 (s, 1H, H_4_), 6.52 (s, 2H, H_2′_, H_6′_), 6.88–7.38 (m, 3H, H_2′_’, H_5′_’ H_6′_’). ^13^C NMR (100 MHz, CDCl_3_): δ 55.48, 55.72, 60.46 (OCH_3_), 72.86 (C_4_), 83.48 (C_3_), 95.52 (C_2′_, C_6′_), 112.12 (C_5′_’), 120.79 (C_2′_’), 124.22 (C_6′_’), 127.86 (C_4′_), 131.16 (C_1′_), 135.05 (C_1′_’), 140.03 (C_3′_’), 151.08 (C_4′_’), 153.10 (C_3′_, C_5′_), 157.79 (C_2_). HRMS: found 506.0200 [M-H]^−^; C_19_H_19_^35^Cl_2_NO_9_P requires 506.0174.

### 3.2. Stability Study for Compound ***16a***

A stability study for compound **16a** was performed by analytical HPLC using a Symmetry^®^ column (C18, 5 mm, 4.6 × 150 mm), a Waters 2487 Dual Wavelength Absorbance detector, a Waters 1525 binary HPLC pump and a Waters 717 plus Autosampler (Waters Corporation, Milford, MA, USA). Samples were detected at λ 254 nm using acetonitrile (70%)/water (30%) as the mobile phase over 15 min and a flow rate of 1 mL/min. A stock solution of the compound was prepared using 10 mg of compound **16a** in 10 mL of mobile phase (1 mg/mL). A calibration curve was prepared using a solution of 0.5, 0.25, 0.125, 0.0625, 0.03125, 0.015625 and 0.0078 mg/mL. (i) *Stability of **16a** in phosphate buffers:* Phosphate buffers at pH values 4, 7.4 and 9 were prepared according to the British Pharmacopoeia 2020. A total of 300 µL of stock solution (1 mg/mL ACN) for **16a** was added to a vial containing 9.7 mL of buffer, mixed and pre-heated to 37°C. A total of 1 mL of the solution was added to the HPLC glass vial and 10 µL was injected, followed by hourly injections for a 24-h period. Samples were withdrawn and analysed at time intervals of t = 0 min, 5 min, 30 min, 60 min and hourly for 24 h. The analysis was performed in triplicate. (ii) *Thermal stability:* **16a** (1 mg) was placed in a vial (for the solution, 1 mL of stock solution was used) at 60 °C for 4 h on a heating block. The sample was then cooled, diluted with ACN and analysed using HPLC. (iii) *Photostability study*: A solution of compound **16a** (1 mL of the stock solution) was placed in a vial and exposed to UV light for 4 h. The sample was directly analysed using HPLC. (iv) *Stability in acidic condition:* The stock solutions (0.8 mL) of **16a** were placed in a vial and HCl (0.1 M, 0.2 mL) was added. The vial was vortexed to ensure a homogeneous mixture and left to stand at room temperature. A sample from the vial was taken and neutralised with NaOH (0.1 M, 0.2 mL) every hour for 4 h. Once neutralised, the samples were analysed using HPLC. (v) *Stability of **16a** in basic (alkaline) conditions*: The stock solution (0.8 mL) was placed in a vial and NaOH (0.1 M, 0.2 mL) was added. The vial was vortexed to ensure a homogeneous mixture and left to stand at room temperature. A sample from the vial was neutralised with HCl (0.1 M, 0.2 mL) every hour for the 4 h. Once neutralised, the samples were analysed using HPLC. (vi) *Stability of **16a** in oxidising conditions:* The stock solution (0.8 mL) was placed in a vial and H_2_O_2_ (3%, 0.2 mL) was added. The vial was vortexed to ensure a homogeneous mixture and left to stand for 4 h at room temperature. A sample from the vial was taken every hour over 4 h and analysed using HPLC. 

### 3.3. Biochemical Evaluation of Activity

All biochemical assays were performed in triplicate on at least three independent occasions for the determination of mean values reported. All the reagents including foetal bovine serum (FBS) and cell culture growth medium (MEM, DMEM and RPMI-1640) were purchased from BD Biosciences. CA-4 was purchased from Sigma Aldrich. 

#### 3.3.1. Cell Culture

The human breast carcinoma cell line MCF-7 was purchased from the European Collection of Animal Cell Cultures (ECACC). Triple negative breast cancer Hs578T cells and its invasive variant Hs578Ts(i)8 were a kind gift from Dr. Susan McDonnell, School of Chemical and Bioprocess Engineering, University College Dublin. U266 cells were a gift from Dr. Tony McElligott, School of Medicine, St James’s Hospital, Trinity College Dublin. HT-29 cells were purchased from the European Collection of Cell Cultures (originating from a human adenocarcinoma of the colon). The human breast carcinoma cell line MDA-MB-231 and the HL-60 cells derived from a patient with acute myeloid leukaemia were both obtained from ECACC (Salisbury, UK). The SW-480 cells were a kind gift from Dr. Brian Flood, School of Biochemistry and Immunology, Trinity College Dublin. HEK-293T (normal epithelial embryonic kidney cells) were cultured in Dulbecco’s Modified Eagle’s Medium (DMEM) with GlutaMAXTM-I in the absence of non-essential amino acids. Human breast cancer MCF-7 cells and multiple myeloma U266 cells were cultured in Minimum Essential Media (MEM) with GlutaMAX™-I, supplemented with 1% (*v*/*v*) non-essential amino acids, 10% 2(*v*/*v*) foetal bovine serum (FBS) and 1% (*v*/*v*) penicillin/streptomycin 5000 U/mL. MDA-MB-231 cells were maintained in DMEM supplemented with 10% (*v*/*v*) foetal bovine serum, 2 mM L-glutamine and 100 μg/mL penicillin/streptomycin (complete medium). Colon cancer HT-29 and SW-480 and triple negative breast cancer Hs578T and its invasive variant Hs578T8i were cultured in DMEM with GlutaMAX™-I, with the same supplement in the absence of non-essential amino acids. Leukaemia HL-60 cancer cells were cultured in Roswell Park Memorial Institute Media (RPMI-1640) with GlutaMAX™-I, supplemented with 10% FCS media, and 100 μg/mL penicillin/streptomycin as above. Cell numbers were monitored using a haemocytometer. Cell culture flasks were incubated in a humidified incubator (5% CO_2_/95% air) at 37 °C. All cell lines were sub-cultured three times per week with trypsinisation using TrypLE Express (1X) required for adherent cell lines. 

#### 3.3.2. Cell Viability Assay

A stock solution of each β-lactam compound was prepared (10 mM) and serial 100× dilutions were made with ethanol for compounds to have working dilutions of 1 nM, 10 nM, 100 nM, 500 nM, 1µM, 5 µM, 10 µM and 50 µM. CA-4 was dissolved in ethanol to obtain a 10 mM stock solution. All stock solutions and serial dilution in ethanol/DMSO were stored at -20 °C. All cells were seeded at a density of 2.5 × 10^4^ cells/mL in a 96-well plate (200 μL per well). The cells were incubated in a 95% O_2_/5% CO_2_ atmosphere at 37 °C for 24 h, then treated with test compound (2 μL of stock solutions per 200-microlitre well) in ethanol to obtain a concentration range of 1 nM–200 μM for the study. The plates were then re-incubated for a further 72 h. Control wells contained an equivalent volume of the vehicle ethanol or DMSO (1% *v*/*v*). *MTT cell viability assay:* The culture medium was removed, and the cells were washed with phosphate buffered saline (PBS, 100 mL). MTT (dissolved in PBS, 50 mL) was added to obtain a final concentration of MTT (1 mg/mL). Cells were incubated at 37 °C for 3 h in the dark. Solubilisation was commenced by the addition of DMSO (200 mL) and the cells were maintained at 20 °C in the dark for 20 min before reading the absorbance to ensure complete colour diffusion. The absorbance value of control cells (no added compound) was set to 100% cell viability and absorbance versus cell density per well was determined to assess cell viability using Graph-Pad Prism software. *AlamarBlue cell viability assay:* Cells were seeded in 96-well plates (e.g., MCF-7, 5 × 10^3^ cells/well) and (HT-29 cells, 1 × 10^3^ cells/well) and (HL-60 cells, 1 × 10^4^ cells/well) with a total volume per well of 200 µL. After 24 h, cells were treated in triplicate with serial dilutions of CA-4 or β-lactam analogues (0.001–100 μM), medium alone or vehicle (1% ethanol (*v*/*v*)). Ethanol or DMSO were used as vehicle control and cells were treated with no more than 1% ethanol (*v*/*v*) or 0.1% DMSO in all experiments. Cell proliferation for cells was analysed using the AlamarBlue assay (Invitrogen Corp.) following the manufacturer’s instructions. After 72 h, AlamarBlue (10% (*v*/*v*) (20 μL)) was added to each well and plates were incubated in the dark at 37 °C for 3–5 h. The blank consisted of the appropriate medium (according to cell type) with the addition of AlamarBlue. Plates were analysed on the 96-well fluorimeter Spectramax Gemini plate reader with excitation at 530 nm and emission at 590 nm using a SOFTmax Pro version 4.9 (Molecular Devices, Sunnyville, C.A) software package and the percentage viability relative to vehicle control was recorded. Results were plotted using GraphPad Prism 5 software and analysed using a non-liner, sigmoidal dose response curve to determine the relative IC_50_ values. All assays were performed in triplicate for the determination of mean values reported.

#### 3.3.3. Lactate Dehydrogenase Assay for Cytotoxicity 

The cytotoxicity of selected compounds was determined using the CytoTox 96 non-radioactive cytotoxicity assay (Promega Corporation, Madison, WI, USA) [104]. Briefly, MCF-7 cells were seeded in a 96-well plate (200 μL per well), at a density of 2.5 × 10^4^ cells/mL, and incubated for 24 h. The cells were then treated with selected β-lactam compounds as described above for the cell viability assay. After 72 h, ‘lysis solution (10X)’ (20 μL) was added to the plate and incubated for a further 1 hr to ensure 100% death. Supernatant (50 μL) was removed from each well to a 96-well plate. Reconstituted ‘CytoTox 96^®^ Reagent (50 μL) was added to each well and the plate was placed in the dark at 20 °C for 30 min. ‘Stop solution’ (50 μL) was added to each well and the samples were analysed at 490 nm using a Dynatech MR5000 plate reader. The percentage cell death at 10 μM was calculated. 

#### 3.3.4. Cell Cycle Analysis

Flow cytometric analysis was used to determine DNA level in any given cell that had been stained with propidium iodide (PI) [105]. In this experiment, adherent and detached cells were collected by trypsinisation and centrifuged at 800× *g* for 15 min. Cells were then washed three times with ice-cold PBS and fixed with slow addition of ice-cold 70% ethanol overnight at −20 °C. The cells were then centrifuged (800× *g*) for 15 min; the pellet was re-suspended in PBS (400 µL) and transferred to LP5 FACS tubes. Cells were then stained with PI (50 μg/mL), containing DNase-free RNase A (50 μg/mL) at 37 °C for 30 min, which degrades any double-strand RNA. The DNA content of the cells (10,000 cells/experimental group) was analysed by flow cytometry at 488 nm using a FACSCalibur flow cytometer (BD Biosciences, San Jose, CA, USA). Results were presented as mean ± SEM. The statistical analysis of experimental data was performed using the program Prism GraphPad 5. A two-way ANOVA (Bonferroni post-test) was used to test for statistical significance (**, *p* < 0.05, ***, *p* < 0.001). A value of *p* < 0.05 was considered to be significant.

#### 3.3.5. Annexin V/PI Apoptotic Assay 

The Annexin V/Propidium Iodide (PI) assay was used to detect both early- and late-stage apoptosis using flow cytometry, as previously reported [34]. Early apoptosis is detected by the presence of phosphatidylserine (PS) on the outer surface of the cell membrane. PS is a phospholipid normally found on the cytoplasmic surface of the cell membrane. In apoptosis, PS is translocated to the outer surface of the cell membrane, and is exposed to the extracellular environment [106]. MCF-7 cells were seeded in 6-well plates (1 × 10^5^ cells/mL) and treated with either vehicle (0.1% (*v*/*v*) EtOH), CA-4 (50 nM) or β-lactam compound **10n** (50 and 500 nM) for 48 h. Cells were then analysed using flow cytometry. Cells were first washed in 1X binding buffer (20X binding buffer: 0.1 M HEPES, pH 7.4; 1.4 M NaCl; 25 mM CaCl_2_ diluted in dH_2_O) and treated for 30 min on ice in Annexin V-containing binding buffer (1:100) in the dark. Cells were washed in binding buffer and then re-suspended in PI-containing binding buffer (1:1000). Samples were analysed without delay using the BD Accuri flow cytometer and the data analysed with GraphPad Prism software.

#### 3.3.6. In Vitro Tubulin Polymerisation Assay

The assembly of purified bovine tubulin was monitored using a kit, BK006, purchased from Cytoskeleton Inc. (Denver, CO, USA) [89] as we have previously reported [34]. Briefly, purified bovine brain tubulin (>99%, 3 mg/mL) in a buffer (80 mM PIPES (pH 6.9), 0.5 mM EGTA, 2 mM MgCl_2_, 1 mM GTP and 10% glycerol) was incubated at 37 °C in the presence of either vehicle (2% (*v*/*v*) ddH_2_O) or β-lactam compounds **10e** and **11n** (10 µM). A reference control experiment with CA-4 was also used (See Supplementary information). Light was scattered proportionally, dependent on the concentration of polymerised microtubules in the sample. Tubulin assembly was monitored turbidimetrically at 37 °C in a Spectramax 340 PC spectrophotometer (Molecular Devices, Sunnyvale, CA, USA) at 340 nm. The absorbance was measured at 30-s intervals for 60 min.

#### 3.3.7. Colchicine Binding-Site Assay

The assay was performed as we have previously reported [74]. *N*,*N*′-Ethylene-bis(iodoacetamide) (EBI) (Santa Cruz Biotechnology) was dissolved in ethanol (100 μM). MCF-7 cells were seeded at a density of 5 × 10^4^ cells/well in 6-well plates and incubated overnight. Cells were treated with vehicle control (ethanol (0.1% *v*/*v*)), colchicine or CA-4 and selected β-lactam compound (all 10 μM) for 2 h. After this time, selected wells were treated with EBI (100 μM) for 1.5 h. Following treatment, cells were twice washed with ice-cold PBS and lysed by addition of Laemmli buffer. Samples were separated by SDS-PAGE, transferred to polyvinylidene difluoride (PVDF) membranes, and probed with β-tubulin antibodies (Sigma Aldrich, Milwaukee, WI, USA) as previously described [74,93].

#### 3.3.8. Immunofluorescence Assay

Confocal microscopy was used to study the effects on MCF-7 cytoskeleton following treatment with compound **10n** following the protocols previously reported [34]. For each experiment, all images were collected on the same day using identical parameters. MCF-7 cells were seeded (1 × 10^5^ cells/mL) on eight chamber glass slides. Cells were either untreated or treated with vehicle (1% ethanol (*v*/*v*)), CA-4 (10 nM), Paclitaxel (1 μM) and β-lactam compound **10n** at 50 nM, 100 nM and 500 nM concentrations for 16 h. The cells were then gently washed in PBS and then fixed for 30 min with 100% ice-cold MeOH. Cells were washed three times in PBS for 10 min and then permeabilised in 0.5% Triton X-100. The cells were subsequently washed in PBS containing 0.1% Tween (PBST), and then blocked in bovine serum albumin (5%) diluted in PBST. Cells were then incubated with a mouse monoclonal anti-α-tubulin−FITC antibody (clone DM1A) (1:200) for 2 h at 20 °C. Following washes in PBST, cells were incubated with Alexa Fluor 488 dye (1:500) for 1 h at 20 °C. Following further washes in PBST, the cells were mounted in Ultra Cruz Mounting Media, which contained 4,6-diamino-2-phenolindol dihydrochloride (DAPI). The images were photographed using Leica SP8 confocal microscopy with the Leica application suite X software program. Experiments were performed on three independent occasions.

#### 3.3.9. Western Blot Analysis

MCF-7 cells were seeded at a density of 1 × 10^5^ cells/flask in T25 flasks. After 48 h, whole cell lysates were prepared from untreated cells or cells treated with vehicle control (Ethanol, 0.1% *v*/*v*) or compound **10n** (0.05, 0.1 and 0.5 μM). MCF-7 cells were harvested in RIPA buffer supplemented with protease inhibitors (Roche Diagnostics), phosphatase inhibitor cocktail 2 (Sigma-Aldrich, St. Louis, MI, USA), and phosphatase inhibitor cocktail 3 (Sigma-Aldrich). Equal quantities of protein (as determined using a BCA assay) were resolved by SDS-PAGE (12%) followed by transfer to PVDF membranes. Membranes were blocked for 1 h using 5% bovine serum albumin/TBST then incubated in the relevant primary antibodies at 4 °C overnight. Membranes were washed with TBST, incubated in horseradish peroxidase conjugated secondary antibody for 1 h at room temperature and washed again. Western blot analysis, using antibodies directed against Bcl-2 (1:500) (Millipore), BAX (1:1000) (Millipore) or Mcl-1 (1:1000) (Millipore), was followed by incubation with a horseradish peroxidase-conjugated anti-mouse antibody [1:2000] (Promega, Madison, WI, USA). Blots were probed with anti-GAPDH antibody (1:5000) (Millipore) to confirm equal loading. Proteins were detected using enhanced chemiluminescent Western blot detection (Clarity Western ECL substrate) (Bio Rad) on the ChemiDoc MP System (Bio Rad). Experiments were performed on two independent occasions.

### 3.4. X-ray Crystallography

Data for samples **9o**, **10o**/**16g** and **11o** were collected on a Bruker APEX DUO using Mo Kα radiation (λ = 0.71073 Å). Each sample was mounted on a MiTeGen cryoloop and data was collected at 100(2) K using an Oxford Cobra cryosystem. Bruker APEX software [107] was used to collect and reduce data, determine the space group and solve and refine the structures. Absorption corrections were applied using SADABS [108]. Data for **10e** were collected on a Rigaku Saturn 724 (Mo Kα radiation, λ = 0.71073 Å) equipped with a Rigaku X-Stream low temperature device. The sample was mounted on a Hampton cryoloop and data were collected at 93(2) K. Data were measured using 0.3° scans per frame for 20 s. A total of 852 frames were collected with a final resolution of 0.77 Å. Data reduction and correction for Lorenz, polarisation and absorption were performed using the CrystalClear software. Absorption corrections were applied using REQAB (Rigaku Inc., 2007). Structures **10o**/**16g** and **11o** were solved with the SHELXT structure solution program [109] using Intrinsic Phasing and **9o** and **10e** with SHELXS with direct methods. All were refined using Least Squares method on F^2^ with SHELXL. All non-hydrogen atoms were refined anisotropically. Hydrogen atoms were assigned to calculated positions using a riding model with appropriately fixed isotropic thermal parameters. Molecular graphics were generated using OLEX2 [110]. In the structure of **10o**, the halogen substituted 3 position on the lactam ring was modelled between Cl and Br with 75:25% of each, respectively. The occupancy was freely refined then fixed. C-Cl and C-Br distances were restrained (DFIX) and the atomic displacement of both halides was also constrained (EADP). In **11o**, the 4 substituent, the Me(OMe)Ph ring, was modelled in two locations with restraints (SADI) and constraints (EADP). The refined occupancies of each moiety were 81:19%. Crystallographic data for the structures in this paper have been deposited with the Cambridge Crystallographic Data Centre as supplementary publication nos. 2077515-2077518. Copies of the data can be obtained, free of charge, on application to CCDC, 12 Union Road, Cambridge CB2 1EZ, UK (fax: +44-(0)1223-336033 or e-mail: deposit@ccdc.cam.ac.uk).

### 3.5. Computational Procedure for Molecular Docking

The 1SA0 X-ray structure of bovine tubulin co-crystallised with N-deacetyl-N-(2-mercaptoacetyl)-colchicine (DAMA-colchicine) was downloaded from the PDB website [6]. A UniProt Align analysis confirmed a 100% sequence identity between human and bovine β tubulin. The crystal structure was prepared using QuickPrep (minimised to a gradient of 0.001 kcal/mol/Å), Protonate 3D, Residue pKa and Partial Charges protocols in MOE 2019 with the MMFF94x force field. Compounds **10n, 11n** and **14b** were drawn in MOE, saved as an mdb and processed in MOE [101]. Both *trans* enantiomers of the compounds **10n**, **11n** and **14b** were examined. For each compound, MMFF94x partial charges were calculated, and each was minimised to a gradient of 0.001 kcal/mol/Å. Default parameters were used for docking except that 300 poses were sampled for each compound and the top 50 docked poses were retained for subsequent analysis. Default settings of OMEGA [111,112] were used to generate 50 conformers of each compound prior to running rigid docking with FRED [113], included in the OEDocking suite [111]. 

## 4. Conclusions

Microtubule-targeting drugs such as taxanes and vinca alkaloids are very effective therapeutic agents in the treatment of various types of cancers. Interestingly, the antiviral activity of the *bis*-indole microtubule targeting drug sabizabulin (VERU-111) against SARS CoV-2 was recently reported [114]. Sabizabulin binds to tubulin and disrupts the intracellular transport of the SARS CoV-2 virus; it also demonstrates an effective anti-inflammatory effect. In this work, a novel series of heterocyclic combretastatin CA-4 compounds based on the β-lactam scaffold were designed and synthesised as tubulin-targeting agents. All the novel compounds were initially evaluated in the MCF-7 breast cancer cell line and of particular interest were compounds **10e**, **10n** and **11n**, which displayed antiproliferative activity in the nanomolar range, e.g., **10e** (IC_50_ = 34 nM), **10n** (IC_50_ = 17.5 nM) and **11n** (IC_50_ = 31 nM) in MCF-7 cells. These compounds were identified for further studies to provide a better understanding of their mechanism of action in breast cancer cells. Minimal cytotoxicity was observed on the treatment of the most potent compound **10n** in the non-tumourigenic cell line HEK-293T, demonstrating the selectivity of the compounds toward cancer cells. The compounds were evaluated in the NCI 60 cancer cell line panel and demonstrated significant antiproliferative activity at nanomolar concentrations in a range of human cancer cell lines. Cell cycle analysis of compound **10n** in MCF-7 cells demonstrated that this compound induces G_2_/M arrest and apoptotic cell death. The induction of apoptosis in MCF-7 cells by compound **10n** was confirmed using flow cytometric analysis of Annexin V/PI-stained cells. An alteration of the expression levels of apoptosis-related proteins Bax, Bcl-2 and Mcl-1 in MCF-7 cells was shown using Western blot analysis. To examine whether the antiproliferative activities might be related to the depolymerisation of tubulin, the inhibitory effects of compounds **10e** and **11n** on tubulin polymerisation were confirmed with the suppression of in vitro tubulin polymerisation. The tubulin depolymerisation effects of compound **10n** were confirmed when MCF-7 cells treated with the β-lactam **10n** displayed a disorganised microtubule network with similar multinucleation effects to CA-4. Tubulin EBI-adduct formation was inhibited in MCF-7 cells treated with **10n**, indicating an interaction with the colchicine binding site of tubulin. Our data strongly indicate that this class of β-lactams represent interesting lead molecules with the potential for a design of potent microtubule-targeting agents and further clinical anti-cancer drug development. 

## Figures and Tables

**Table 1:** Crystal Data and Structure Refinement Details for compounds **9o**, **10e**, **10o**/**16g** and **11o**.

Identification Code	9o	10e	10o/16g	11o
CCDC number	2077515	2077516	2077517	2077518
Empirical formula	C_18_H_21_NO_3_S	C_19_H_20_ClNO_5_	C_19_H_19_Br_0.25_Cl_1.75_NO_5_	C_20_H_21_Cl_2_NO_5_
*M* (g/mol)	331.42	377.81	423.37	426.28
*T (K)*	100(2)	93(2)	100(2)	100(2)
Crystal System	monoclinic	monoclinic	monoclinic	orthorhombic
SG	P2_1_/c	P2_1_/n	P2_1_/n	Pbca
*a* (Å)	12.3686(4)	10.138(2)	10.3414(3)	11.2765(7)
*b* (Å)	10.1330(3)	9.840(2)	10.0786(3)	12.5130(6)
*c* (Å)	13.8346(5)	18.357(4)	17.9994(6)	27.9482(15)
*α* (°)	90	90	90	90
*β* (°)	100.7347(12)	95.46(3)	96.2023(15)	90
*γ* (°)	90	90	90	90
*V* (Å^3^)	1703.56(10)	1822.9(6)	1865.04(10)	3943.6(4)
*Z*	4	4	4	8
*Dcalc* (g/cm^3^)	1.292	1.377	1.508	1.436
*μ* (mm^−1^)	0.204	0.239	0.879	0.361
F(000)	704.0	792.0	874.0	1776.0
Crystal size (mm^3^)	0.162 × 0.08 × 0.05	0.320 × 0.280 × 0.260	0.38 × 0.36 × 0.12	0.38 × 0.06 × 0.05
Radiation	Mo Kα (λ = 0.71073)	Mo Kα (λ = 0.71073)	Mo Kα (λ = 0.71073)	Mokα (λ = 0.71073)
Reflections collected	54356	16596	20607	11396
Independent reflections	3907 R_int_ = 0.0396 R_sigma_ = 0.0188	4026 R_int_ = 0.0226 R_sigma_ = 0.0175	4649 R_int_ = 0.0439 R_sigma_ = 0.0369	3614 R_int_ = 0.0534 R_sigma_ = 0.0648
Data/restraints/parameters	3907/0/212	4026/0/235	4649/8/251	3614/1/246
Goodness-of-fit on *F*^2^ (*S*)	1.049	1.079	1.036	1.078
Final R indexes (*I* ≥ 2σ (*I*)) *	R_1_ = 0.0430, wR_2_ = 0.1126	R_1_ = 0.0364, wR_2_ = 0.0877	R_1_ = 0.0313, wR_2_ = 0.0741	R_1_ = 0.0525, wR_2_ = 0.1026
Final R indexes (all data)	R_1_ = 0.0565, wR_2_ = 0.1207	R_1_ = 0.0373, wR_2_ = 0.0882	R_1_ = 0.0441, wR_2_ = 0.0795	R_1_ = 0.0779, wR_2_ = 0.1126
Largest diff. peak/hole/e Å^−3^	0.66/−0.55	0.35/−0.25	0.35/−0.38	0.30/−0.45

* R_1_ = ∑ ||F_o_| − |F_c_||/ ∑ |F_o_|, wR_2_ = [ ∑ w(F_o_^2^ − F_c_^2^)^2^/ ∑ w(F_o_^2^)^2^]^1/2^.

**Table 2:** X-ray Crystallographic Data for β-lactams **10e**, **10o**, **11o** and **16g**.

Compound	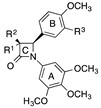	Ring Plane Normal AB Angle (°)	Ring Plane Normal BC Angle (°)	Ring Plane Normal AC Angle (°)	Ring A to Central Torsion (°) *^a^*	Ring B to Central Torsion (°) *^b^*	Ring AB Torsion (°) *^c^*	Ring BC Torsion (°) *^d^*
**10e**	R^1^=Cl, R^3^=R^3^=H	81.20(5)	71.33(6)	20.68(6)	−24.7(2)	168.6(1)	−60.7(2)	116.0(1)
**10o**	R^1^=R^3^=Cl, R^2^=H	91.49(5)	80.74(7)	159.88(6)	27.8(2)	−156.7(1)	62.7(2)	−112.0(3)
**11o**	R^1^=R^2^=Cl, R^3^=CH_3_	91.7(1)	103.7(1)	17.3(1)	−15.3(4)	160.6(4)	−68.9(4)	139.5(3)
**16g**	R^1^=Br, R^2^=H, R^3^=Cl	91.49(5)	80.74(7)	159.88(6)	27.8(2)	−156.7(1)	62.7(2)	−110.3(4)

*^a^* C18-C13-N1-C2, C18-C13-N1-C2, C22-C17-N1-C2, C18-C13-N1-C2; *^b^* C10-C5-C4-N1, C10-C5-C4-N1, C10-C5-C4-N1, C10-C5-C4-N1; *^c^* C13-N1-C4-C5, C13-N1-C4-C5, C12-N1-C4-C5, C13-N1-C4-C5; *^d^* C5-C4-C3-Cl1, C5-C4-C3-Cl1, C5-C4-C3-Cl1, C5-C4-C3-Br1 for **10e**, **10o**, **11o** and **16g**, respectively.

**Table 3:** Antiproliferative activity of β-lactam compounds **10a**–**o**, **11a**–**o**, **12a**–**c**, **13a**–**c**, **14a**, **14b**, **15a**, **15b**, **16a**–**j**, **18** and CA-4 in MCF-7 cells.

Compound ^a^	Compound Number		IC_50_, µM (MCF-7, 72 h) ^b,f^	LogP ^e^
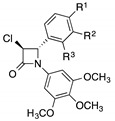	**10a**	R^1^=R^2^=R^3^=H	23.450 ± 2.80	3.48
	**10b**	R^1^=Cl, R^2^=R^3^=H	1.298 ± 0.17	4.20
	**10c**	R^1^=Br, R^2^=R^3^=H	0.506 ± 0.11	4.35
	**10d**	R^1^=NO_2._ R^2^=R^3^=H	4.366 ± 0.61	3.23
	**10e *cis***	R^1^=OCH_3_, R^2^=R^3^=H(*cis*)	0.317 ± 0.04	3.40
	**10e *trans ^g^***	R^1^=OCH_3_, R^2^=R^3^=H(*trans*)	0.034 ± 0.004	3.40
	**10f ^g^**	R^1^=OCH_2_CH_3,_ R^2^=R^3^=H	0.130 ± 0.017	3.93
	**10g**	R^1^=OC_6_H_5_, R^2^=R^3^=H	64.070 ± 7.30	5.58
	**10h**	R^1^=OCH_2_C_6_H_5_, R^2^=R^3^=H	59.910 ± 6.99	5.17
	**10i^g^**	R^1^=SCH_3,_ R^2^=R^3^=H	0.073 ± 0.004	4.04
	**10j**	R^2^R^3^=CH=CH-CH=CH, R^1^=H	14.660 ± 2.26	4.66
	**10k**	R^1^R^2^=CH=CH-CH=CH, R^3^=H	0.202 ± 0.015	4.66
	**10m**	R^1^=OCH_3_, R^2^=NO_2,_ R^3^=H	3.088 ± 0.41	3.17
	**10n**	R^1^=OCH_3_, R^2^=OH, R^3^=H	0.0175 ± 0.009	2.67
	**10o ^h^**	R^1^=OCH_3_, R^2^=Cl, R^3^=H	0.433 ± 0.050	4.03
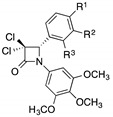	**11a**	R^1^=R^2^=R^3^=H	37.220 ± 3.21	4.67
	**11b**	R^1^=Cl, R^2^=R^3^=H	12.830 ± 1.08	5.38
	**11c**	R^1^=Br, R^2^=R^3^=H	18.160 ± 1.83	5.53
	**11d**	R^1^=NO_2_, R^2^=R^3^=H	61.640 ± 7.61	4.41
	**11e ^g^**	R^1^=OCH_3,_ R^2^=R^3^=H	0.119 ± 0.012	4.59
	**11f ^g^**	R^1^=OCH_2_CH_3,_ R^2^=R^3^=H	0.164 ± 0.015	5.11
	**11g**	R^1^=OC_6_H_5_, R^2^=R^3^=H	62.310 ± 10.2	6.77
	**11h**	R^1^=OCH_2_C_6_H_5_, R^2^=R^3^=H	36.340 ± 3.68	6.35
	**11i ^g^**	R^1^=SCH_3_, R^2^=R^3^=H	0.291 ± 0.030	5.23
	**11j**	R^2^R^3^=CH=CH-CH=CH, R^1^=H	7.990 ± 0.70	5.84
	**11k**	R^1^R^2^=CH=CH-CH=CH, R^3^=H	0.322 ± 0.04	5.84
	**11m**	R^1^=OCH_3_, R^2^=NO_2,_ R^3^=H	0.353 ± 0.04	4.35
	**11n**	R^1^=OCH_3_, R^2^=OH, R^3^=H	0.031 ± 0.004	3.85
	**11o ^h^**	R^1^=OCH_3_, R^2^=CH_3,_ R^3^=H	0.747 ± 0.060	5.09
	**18**	R^1^=OCH_3_, R^2^=OP(O)(OH)_2_, R^3^=H	0.077 ± 0.01	2.80
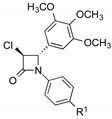	**12a**	R^1^=OCH_3_,	14.810 ± 1.58	3.40
	**12b**	R^1^=OCH_2_CH_3_	25% (10 μM) ^c^	3.93
	**12c**	R^1^=SCH_3_	10% (10 μM) ^c^	4.04
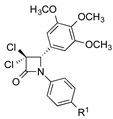	**13a**	R^1^=OCH_3_	45.9 ± 6.73	4.59
	**13b**	R^1^=OCH_2_CH_3_,	40% (10 μM)^c^	5.11
	**13c**	R^1^=SCH_3_,	20% (10 μM)^c^	5.23
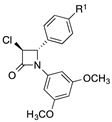	**14a**	R^1^=OCH_3_	0.680 ± 0.160	4.11
	**14b**	R^1^=OCH_2_CH_3_	0.045 ± 0.01	4.64
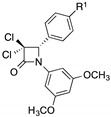	**15a**	R^1^=OCH_3_	6.612 ± 2.00	5.29
	**15b**	R^1^=OCH_2_CH_3_	0.273 ± 0.010 ^i^	5.82
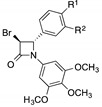	**16a**	R^1^=OCH_3_, R^2^=H	0.579 ± 0.030 ^i^	3.54
	**16b**	R^1^=OCH_2_CH_3_, R^2^=H	1.144 ± 0.300 ^i^	4.07
	**16c**	R^1^= SCH_3_, R^2^=H	0.613 ± 0.040 ^i^	4.18
	**16d**	R^1^=SCH_2_CH_3_, R^2^=H	1.100 ± 0.1 ^i^	4.71
	**16e**	R^1^=OCH_3_, R^2^= CH_3_	0.525 ± 0.03	4.04
	**16f**	R^1^=OCH_3_, R^2^=F	0.390 ± 0.03 ^i^	3.63
	**16g**	R^1^=OCH_3_, R^2^=Cl	0.472 ± 0.20 ^i^	4.16
	**16h**	R^1^=OCH_3_, R^2^=Br	0.620 ± 0.040 ^i^	4.32
	**16j**	R^1^=OCH_3_, R^2^=OH	40% (10 μM) ^c,i^	2.80
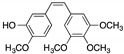	**2a**	CA-4	0.0039 ± 0.00032 ^d^	3.32

^a^ 3-Chloro and 3-bromo-β-lactam compounds are *trans* isomers except where specifically indicated. One enantiomer only is shown. ^b^ IC_50_ values are half maximal inhibitory concentrations required to block the growth stimulation of MCF-7 cells. Values represent the mean ± SEM (error values × 10^−6^) for at least three experiments performed in triplicate. ^c^ IC_50_ values were not calculated; the percentage shown is the inhibition of cell viability at a concentration of 10 μM unless otherwise indicated. ^d^ The IC_50_ value obtained for CA-4 (0.0039 μM for MCF-7) is in good agreement with the reported values, Ref 74. ^e^ clogP values calculated from ChemBioDraw 13.0. ^f^ Lactate Dehydrogenase assay: % cell lysis at compound concentration of 10 μM following treatment of the cells, **3a** (12%), **10e** (5%), **10j** (4%), **10i** (3%), **10n** (17%), **11e** (10%) and **11n** (8%) using LDH assay kit from Promega, Ref 104. ^g^ Ref 75. ^h^ Ref 34. ^i^ SW480 Cell viability: **16a** (52%), **16b** (50%), **16c** (32%), **16d** (22%), **16e** (40%), **16f** (42%), **16g** (38%), **16h** (26%) and **16j** (48%); percentage shown is the inhibition of cell viability at a concentration of 10 μM.

**Table 4:** Antiproliferative Effects of Selected β-Lactam Compounds in MDA-MB-231 Human Breast Cancer Cells.

Compound Number	Antiproliferative Activity ^a,b^ IC_50_ (µM) MDA-MB-231, 72 h
**10e**	0.0686
**10f**	0.078 ± 0.008
**10k**	0.205 ± 0.034
**11e**	0.297 ± 0.04
**11f**	0.389 ± 0.05
**11n**	0.0316
**CA-4 ^c^**	0.043

^a^ IC_50_ values are half maximal inhibitory concentrations required to block the growth stimulation of MDA-MB-231 cells. Values represent the mean (SEM) for three experiments performed in triplicate. ^b^ The results for compounds **10e** and **11n** of antiproliferative activity against MDA-MB-231 were from NCI. ^c^ The IC_50_ values obtained for CA-4 **2a** in this assay are 0.043 μM for MDA-MB-231 and is in good agreement with the reported values for **2a** using the MTT assay on MDA-MB-231 breast cancer cell lines (see Ref 79).

**Table 5:** Antiproliferative effects of selected β-lactam compounds **10e**, **10n** and **11n** in MCF-7 human breast, HL-60 leukaemia, U266 multiple myeloma, SW480 and HT-29 colon cancer cell lines.

Compound	MCF-7 IC_50_ (μM) ^a^	HL-60 IC_50_ (μM) ^a^	U266 IC_50_ (μM) ^a^	SW480 IC_50_ (μM) ^a^	HT-29 IC_50_ (μM) ^a^
**10e**	0.056 ± 0.007	0.161 ± 0.020	0.077 ± 0.02	0.055 ± 0.004	0.135 ± 0.060
**10n**	0.017 ± 0.009	0.010 ± 0.008	0.031 ± 0.01	0.037 ± 0.005	0.631 ± 0.030
**11n**	0.031 ± 0.004	0.016 ± 0.005	nd ^c^	0.044 ± 0.008	0.941 ± 0.060
**CA-4**	0.0039 ± 0.00032 ^b^	0.0019 ± 0.0005	0.035 ± 0.01	0.003 ± 0.0008	4.165 ± 0.1

^a^ IC_50_ values are half maximal inhibitory concentrations required to block the growth stimulation of MCF-7, HL-60, U266, SW480 and HT-29 cells. Values represent the mean (SEM error values 10^−6^) for three experiments performed in triplicate at 72 h. ^b^ The IC_50_ value obtained for CA-4 in this assay is in good agreement with the reported value (see refs 75, 82, 83). ^c^ nd: not determined.

**Table 6:** Antitumour Evaluation of compounds **10e**, **11n** and **16d** in NCI-60 cell line screen ^a^.

Cell Line		Compound 10e	Compound 11n	Compound 16d
		GI_50_ (μM) ^b,c^	GI_50_ (μM) ^b,d^	GI_50_ (μM) ^b,e,j^
***NSC Lung Cancer***	A549/ATCC	0.0785	0.0382	0.598
	EKVX	0.0694	0.0464	0.639
	HOP-62	0.0538	0.0350	*0.336*
	HOP-92	Nd ^f^	Nd ^f^	0.443
	NCI-H226	0.0557	0.0368	3.11
	NCI-H23	0.0312	0.0242	0.478
	NCI-H332M	0.962	Nd ^f^	7.37
	NCI-H460	0.306	0.0354	0.539
	NCI-H552	0.0207	0.0164	0.158
***Colon Cancer***	COLO205	0.0492	2.91	0.230
	HCT-2998	0.0804	0.0534	0.673
	HCT-116	0.0397	0.0328	0.222
	HCT-15	0.0504	0.0383	0.210
	HT29	0.0292	2.78	0.351
	KM12	0.0393	0.0318	0.202
	SW-620	0.0432	0.0376	0.236
***CNS Cancer***	SF-268	0.158	0.0514	1.18
	SF295	0.0254	0.0387	0.0870
	SF539	Nd ^f^	Nd ^f^	0.161
	SNB-19	0.0746	0.0457	0.440
	SNB-75	0.0382	0.0199	0.509
	U251	0.0370	0.0325	0.345
***Prostate cancer***	PC-3	0.0441	0.0353	0.297
	DU-145	0.0446	0.0359	0.349
***Melanoma***	LOX IMV1	0.0676	0.0415	0.834
	MALME-3M	0.0884	0.0685	11.3
	M14	0.0396	0.0313	0.128
	MDA-MB-435	0.0226	0.0202	0.0377
	SK-MEL-2	0.0450	0.0316	0.484
	SK-MEL-28	0.0696	0.0629	1.94
	SK-MEL-5	0.0312	0.0225	0.0861
	UACC-257	23.3	0.158	14.3
	UACC-62	0.0518	0.0422	0.0972
	IGROV1	0.0580	0.0445	0.0630
	OVCAR-3	0.0241	0.0244	0.109
***Ovarian Cancer***	OVCAR-4	0.106	0.0741	1.43
	OVCAR-5	0.0806	0.312	0.431
	OVCAR-8	Nd ^f^	Nd ^f^	0.403
	NCI/ADR-RES	0.0353	0.0265	0.125
	SK-OV-3	0.182	0.0254	0.392
	786-0	0.0694	0.0412	0.340
	A498	0.0271	0.0226	0.103
	ACHN	0.0971	0.0511	Nd ^f^
***Renal Cancer***	CAKI-1	0.122	0.126	Nd ^f^
	RXF 393	0.0271	0.0300	0.170
	SN12C	0.0837	0.0446	0.925
	TK-10	0.0922	0.126	14.3
	UO-31	0.0644	0.0821	0.342
	MCF-7	0.0343	0.0310	0.0447
***Breast Cancer***	MDA-MB-231/ATCC	0.0686	0.0316	0.488
	HS 578T	0.0553	0.0428	0.229
	BT-549	0.174	0.0397	0.525
	T-47D	15.8	11.6	16.9
	MDA-MB-468	0.0508	0.0226	0.154
	MID GI_50_^g^	0.0741	0.0537	0.407
	MID TGI ^h^	23.4	10.7	10.0
	MID LC_50_^i^	81.3	91.2	83.2

^a^ Data obtained from NCI in vitro human tumour cell screen 5 dose assay. ^b^ GI50 is the molar concentration of the compound causing a 50% inhibition of growth of the tumour cells; ^c^ NSC 762040; ^d^ NSC 762041; ^e^ NSC 792961; ^f^ Nd: Not determined; ^g^ MID GI50: MG-MID Full panel mean-graph mid-point; ^h^ TGI (total growth inhibition) TGI is the mean concentration required to completely inhibit the growth of all cells over the total NCI 60 cell line panel; ^i^ LC50 is the mean concentrations required to kill 50% of the cells over the total NCI 60 cell line panel in the assay. ^j^ GI50 values determined in leukaemia cell lines for compound **16d** are CCRF-CEM (0.278), HL-60(TB) (0.214), K-562 (0.0545), MOLT-4 (0.347), RPMI-8226 (0.361) and SR (0.0765) μM.

**Table 7:** Summary of NCI 60 cell line screening results for selected compounds.

Compound	NCI Ref. No	Structure	GI_50_ (nM) ^a^	TGI (µM) ^b^	LC_50_ (µM) ^a^
**10e**	D-762040	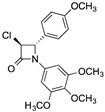	74.13	10.72	91.20
**11n**	D-762041	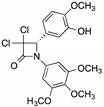	53.70	10.00	83.18
**16d**	S-792961	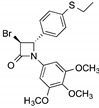	407	23.44	81.28
**2a CA-4**	D-613729	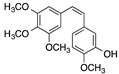	99.30	10.30	85.50

^a^ GI_50_ and LC_50_ are the concentrations required to inhibit the growth and kill 50% of the cells in the assay, respectively. ^b^ TGI is the concentration required to completely inhibit the growth of all cells.

**Table 8:** Inhibition of tubulin polymerisation for compounds **10e**, **11n** and **CA-4** and their corresponding IC_50_ values in MCF-7 and MDA-MB-231 breast cancer cells.

Compound Number	MCF-7 IC_50_ (µM) ^a^	MDA-MB-231 IC_50_ (µM) ^a^	V_max_ at 10 µM (mOD/min) ^b^	Fold-Reduction ^c^
**10e**	0.034 ± 0.004	0.0686	0.0039	1.8
**11n**	0.031 ± 0.005	0.0316	0.0041	1.7
**CA-4**	0.0039 ± 0.00032	0.043	0.0022	6.3

^a^ IC_50_ values are the compound concentration required to inhibit cell proliferation by 50%. Data are expressed as the mean ± S.E.M. from the dose–response curves of at least three independent experiments performed in triplicate. ^b^ The V_max_ values at 10 µM (mOD/min) for the vehicle ethanol is 0.0069. ^c^ Fold inhibition of tubulin polymerisation was calculated using V_max_ value for each reaction.

## Data Availability

Data sharing not applicable.

## Supplementary Materials

The following are available online at https://www.mdpi.com/1424-8247/14/11/1119/s1: Table S1: Tier-1 Profiling Screen of Selected 3-chloroazetidinones, 3,3-dichloroazetidinones and related compounds; Table S2: ADMET and Lipinski Properties for Selected 3-chloroazetidinones, 3,3-dichloroazetidinones and related compounds; Table S3: Standard COMPARE Analysis of compounds 10e, 11n and 16d; Table S4: Cell cycle analysis of MCF-7 cells following treatment with 10n. Figure S1: Effect of control compound CA-4 on tubulin polymerization in vitro; Figures S2–S21: ^1^H NMR and ^13^C NMR spectra.

## Abbreviations

ADC	Antibody-drug conjugate
ATR	Attenuated total reflection
CA-4	Combretastatin A-4
DAMA	N-deacetyl-N-(2-mercaptoacetyl)colchicine
DBU	1,8-Diazabicyclo[5.4.0]undec-7-ene
DCM	Dichloromethane
DEPT	Distortionless Enhancement by Polarisation Transfer
DMEM	Dulbecco’s Mod
DMSO	Dimethylsulfoxide
ECACC	European Collection of Animal Cell Cultures
EGFR	Epidermal growth factor receptor
ER	Estrogen receptor
FACS	Fluorescence activated cell sorting
FBS	Foetal bovine serum
GI_50_	50% Growth inhibitory concentration
HER2	Human epidermal growth factor receptor 2
HDBC	Hormone-dependent breast cancer
HR	Hormone receptor
IC_50_	Half-maximal inhibitory concentration
LC_50_	Median lethal concentration
MDR	Multidrug resistant
MEM	Minimum essential media
MTA	Microtubule-targeting agent
NCI	National Cancer Institute
NMR	Nuclear magnetic resonance
NSCLC	Non-small-cell lung cancer
PBS	Phosphate buffered saline
PBST	Phosphate buffered Saline with Tween^®^ 20
PI	Propidium iodide
PIK3	Phosphatidylinositol-4,5-Bisphosphate 3-Kinase
PR	Progesterone receptor
SERM	Selective estrogen receptor modulator
*t*-BAF	*tert-*Butylammonium fluoride
TBDMSCl	*tert*Butyldimethylsilyl chloride
TGI	Total growth inhibitory concentration
THF	Tetrahydrofuran
TLC	Thin layer chromatography
TNBC	Triple-negative breast cancer
TPSA	Topological polar surface area
VDA	Vascular targeting agent
