# Correction: Malebari et al. Synthesis and Antiproliferative Evaluation of 3-Chloroazetidin-2-ones with Antimitotic Activity: Heterocyclic Bridged Analogues of Combretastatin A-4. *Pharmaceuticals* 2021, *14*, 1119

**DOI:** 10.3390/ph18081219

**Published:** 2025-08-19

**Authors:** Azizah M. Malebari, Shu Wang, Thomas F. Greene, Niamh M. O’Boyle, Darren Fayne, Mohemmed Faraz Khan, Seema M. Nathwani, Brendan Twamley, Thomas McCabe, Daniela M. Zisterer, Mary J. Meegan

**Affiliations:** 1 Department of Pharmaceutical Chemistry, College of Pharmacy, King Abdulaziz University, Jeddah 21589, Saudi Arabia; amelibary@kau.edu.sa; 2 School of Pharmacy and Pharmaceutical Sciences, Trinity College Dublin, Trinity Biomedical Sciences Institute, 152-160 Pearse Street, Dublin 2, DO2R590 Dublin, Ireland; wangsh@tcd.ie (S.W.); tgreene@tcd.ie (T.F.G.); niamh.oboyle@tcd.ie (N.M.O.); 3 Molecular Design Group, School of Biochemistry and Immunology, Trinity College Dublin, Trinity Biomedical Sciences Institute, 152-160 Pearse Street, Dublin 2, DO2R590 Dublin, Ireland; fayned@tcd.ie (D.F.); mfkhan@tcd.ie (M.F.K.); 4 School of Biochemistry and Immunology, Trinity College Dublin, Trinity Biomedical Sciences Institute, 152-160 Pearse Street, Dublin 2, DO2R590 Dublin, Ireland; seema.nathwani@outlook.com (S.M.N.); dzistrer@tcd.ie (D.M.Z.); 5 School of Chemistry, Trinity College Dublin, Dublin 2, DO2R590 Dublin, Ireland; twamleyb@tcd.ie (B.T.); tmccabe@tcd.ie (T.M.)

## Error in Figure and Legend

In the original publication [1], there was a mistake in Figure 8 as published. The corrected Figure 8 and Legend appears below. 

## Text Correction

There was an error in the original publication [1]. A correction has been made to Section 3.3.8. Immunofluorescence Assay:

Confocal microscopy was used to study the effects on MCF-7 cytoskeleton following treatment with compound **10n** following the protocols previously reported [34]. For each experiment, all images were collected on the same day using identical parameters. MCF-7 cells were seeded (1 × 10^5^ cells/mL) on eight chamber glass slides. Cells were either untreated or treated with vehicle (1% ethanol (*v*/*v*)), CA-4 (10 nM), Paclitaxel (1 μM) and β-lactam compound **10n** at 50 nM, 100 nM and 500 nM concentrations for 16 h. The cells were then gently washed in PBS and then fixed for 30 min with 100% ice-cold MeOH. Cells were washed three times in PBS for 10 min and then permeabilised in 0.5% Triton X-100. The cells were subsequently washed in PBS containing 0.1% Tween (PBST), and then blocked in bovine serum albumin (5%) diluted in PBST. Cells were then incubated with a mouse monoclonal anti-α-tubulin−FITC antibody (clone DM1A) (1:200) for 2 h at 20 °C. Following washes in PBST, cells were incubated with Alexa Fluor 488 dye (1:500) for 1 h at 20 °C. Following further washes in PBST, the cells were mounted in Ultra Cruz Mounting Media, which contained 4,6-diamino-2-phenolindol dihydrochloride (DAPI). The images were photographed using Leica SP8 confocal microscopy with the Leica application suite X software program. Experiments were performed on three independent occasions.

The authors state that the scientific conclusions are unaffected. This correction was approved by the Academic Editor. The original publication has also been updated.

## Figures and Tables

**Figure 8:**
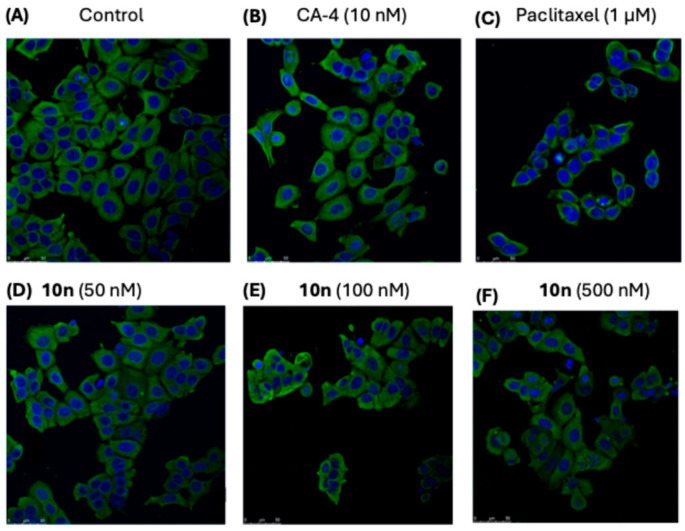
Compound **10n** depolymerises the microtubule network of MCF-7 breast cancer cells. Cells were treated with (**A**) vehicle control (0.1% ethanol (*v*/*v*)), (**B**) CA-4 (10 nM), (**C**) paclitaxel (1 μM) and compound **10n**, (**D**) (50 nM), (**E**) (100 nM) and (**F**) (500 nM) for 16 h. Cells were fixed in ice-cold methanol and stained with mouse monoclonal anti-α-tubulin–FITC antibody (clone DM1A) (green) and Alexa Fluor 488 dye, and counterstained with DAPI (blue). Images were obtained with Leica SP8 confocal microscopy with Leica application suite X software. Representative confocal micrographs of three separate experiments are shown. Scale bar indicates 25 μm.
