# Supplementary material for: Synthesis and Antiproliferative Evaluation of 3-Chloroazetidin-2-ones with Antimitotic Activity: Heterocyclic Bridged Analogues of Combretastatin A-4
Source: Pharmaceuticals (Basel). 2021 Oct 31;14(11):1119. doi: 10.3390/ph14111119 (PMC8624998; doi:10.3390/ph14111119)
Supplement: Supplementary file 1 [file pharmaceuticals-14-01119-s001.zip › pharmaceuticals-1441951-supplementary.pdf]

Supplementary Information

Synthesis and antiproliferative evaluation of 3-chloroazetidin-2-ones with antimitotic activity: Heterocyclic bridged analogues of Combretastatin A-4

Azizah M. Malebari*[1], Shu Wang*[2], Thomas F. Greene[2], Niamh M. O'Boyle[2], Darren Fayne[3], Mohemmed Faraz Khan[3], Seema M. Nathwani[4], Brendan Twamley[5], Thomas McCabe[5], Daniela M. Zisterer[4] and Mary J. Meegan[2,]**

[1] Department of Pharmaceutical Chemistry, College of Pharmacy, King Abdulaziz University, Jeddah 21589, Saudi Arabia; amelibary@kau.edu.sa (A.M.M.).

[2] School of Pharmacy and Pharmaceutical Sciences, Trinity College Dublin, Trinity Biomedical Sciences Institute, 152-160 Pearse Street, Dublin 2 DO2R590, Ireland; wangsh@tcd.ie (S.W.); tgreene@tcd.ie (T.F.G.); Niamh.OBoyle@tcd.ie (N.M.O'B.)

[3] Molecular Design Group, School of Biochemistry and Immunology, Trinity College Dublin, Trinity Biomedical Sciences Institute, 152-160 Pearse Street, Dublin 2 DO2R590, Ireland; FAYNED@tcd.ie (D.F.): mfkhan@tcd.ie (M.F.K.)

[4] School of Biochemistry and Immunology, Trinity College Dublin, Trinity Biomedical Sciences Institute, 152-160 Pearse Street, Dublin 2 DO2R590, Ireland; seema.nathwani@outlook.com (S.M.N.); dzistrer@tcd.ie (D.M.Z.)

[5] School of Chemistry, Trinity College Dublin, Dublin 2 DO2R590, Ireland; TWAMLEYB@tcd.ie (B.T.); TMCCABE@tcd.ie (T.M.)

* Co-First Authors: contributed equally to the work

** Correspondence: mmeegan@tcd.ie (M.J.M.); Tel.: +353-1-896-2798; Fax: +353-1-8962793

Supplementary Information

Chemistry Experimental

Table S1: Tier-1 Profiling Screen of Selected 3-chloroazetidinones, 3,3-dichloroazetidinones and related compounds

Table S2: ADMET and Lipinski Properties for Selected 3-chloroazetidinones, 3,3-dichloroazetidinones and related compounds

Table S3: Standard COMPARE Analysis of compounds **10e**, **11n** and **16d**

Table S4: Cell cycle analysis of MCF-7 cells following treatment with **10n**.

Figure S1: Effect of control compound CA-4 on tubulin polymerization *in vitro*

Figures S2-S21: ^{1}H NMR and ^{13}C NMR spectra

References

Chemistry Experimental

All chemicals were commercial purchased and were used without further purification unless otherwise indicated. Solvents were either purchased dry or purified by distillation in accordance with literature methods. Dichloromethane was dried by distillation from calcium hydride prior to use. Tetrahydrofuran (THF) was distilled immediately prior to use from Na/Benzophenone under a slight positive pressure of nitrogen. Toluene was dried by distillation from calcium hydride and stored on activated molecular sieves (4Å). Melting points were determined on a Gallenkamp SMP 11 melting point apparatus using a mercury 300 ˚C thermometer and are uncorrected. Infra-red (IR) spectra were recorded as KBr discs or as thin films on NaCl disk on a Perkin Elmer FT-IR Paragon 1000 spectrometer. ^{1}H and ^{13}C nuclear magnetic resonance (NMR) spectra were recorded at 20 ˚C on a Brucker DPX 400 spectrometer (400.13 MHz, ^{1}H; 100.61 MHz, ^{13}C) in deuterated chloroform ($CDCl_3$), DMSO-d_6 or methanol (CD_3OD) (internal standard tetramethylsilane (TMS)) by Dr. John O'Brien and Dr. Manuel Ruether in the School of Chemistry, Trinity College Dublin. Coupling constants were reported in Hertz (Hz). Abbreviations used in the assigning of the spectra include s = singlet, br s = broad singlet, d = doublet, t = triplet, q = quartet and m = multiplet. For ^{1}H-NMR assignments, chemical shifts were reported: shift value (description of absorption, coupling constant(s) where applicable, and number of protons). High resolution mass spectrometry (HRMS) was obtained in the School of Chemistry by Dr Martin Feeney or School of Pharmacy and Pharmaceutical Sciences by Mr Brian Talbot, Trinity College Dublin. HRMS was carried on in the positive ion mode on a liquid chromatography time-of flight mass spectrometer (Micromass LCT, Waters Ltd., Manchester, UK). The samples were introduced into the ion source by an LC system (Waters Alliance 2795, Waters Corporation, USA) in acetonitrile:water (60:40% v/v) at 200 µL/min. The capillary voltage of the mass spectrometer was at 3 kV. The sample cone (de-clustering) voltage was set at 40 V. For exact mass determination, the instrument was externally calibrated for the mass range m/z 100 to m/z 1000. A lock (reference) mass (m/z 556.2771) was used. High resolution mass spectrometry scans were also performed using Electrospray Ionization operated in negative and positive ion modes on a LTQ / Orbitrap Discovery Mass Spectrometer, and samples were dissolved in CH_3OH. Mass measurement accuracies of < ±5 ppm were obtained. Low resolution mass spectra (LRMS) were acquired on a Hewlett-Packard 5973 MSD GC-MS system in electron impact (EI) mode. Thin layer chromatography was performed using Merck silica gel 60 TLC aluminium sheets with fluorescent indicator visualizing with UV light at 254 nm. Flash column chromatography was carried out on Merck Kiesegel 60 (particle size 0.040 mm-0.063 mm).

Chromatographic separations were also carried out on Biotage SP4 instrument. All products isolated were homogenous on TLC. Microwave experiments were performed with the Biotage initiator and Discover CEM microwave synthesizers. Determination of the purity of the finial compounds was achieved by analytical high-performance liquid chromatography (HPLC) using a Waters 2487 Dual Wavelength Absorbance detector, Waters 1525 binary HPLC pump, Waters In-Line Degasser AF and Waters 717plus Autosampler. The column used was a Varian Pursuit XRs C18 reverse phase 250 × 4.6 mm chromatography column. Samples were detected using a wavelength of 254 nm. All samples were analysed using acetonitrile (60%):water (40%) with 0.1% (v/v) TFA over 10 minutes and a flow rate of 1 mL/min.

3-(*t*-Butyl-dimethyl-silanyloxy)-4-methoxy-benzaldehyde

To a solution of 3-hydroxy-4-methoxybenzaldehyde (5 mmol) and *t*-butyl dimethylsilylchloride (6 mmol) in anhydrous DCM (40 mL) under a nitrogen atmosphere, DBU (8 mmol) was added dropwise *via* a syringe. The reaction mixture was stirred under nitrogen for 4 h (monitored by TLC (50:50 hexane/ethyl acetate) until reaction was complete. The reaction mixture was diluted with dichloromethane (50 mL) and washed with water (2 x 100mL), HCl (0.1M, 2 x 50 mL), saturated $NaHCO_3$ aqueous solution (2 x 50 mL), and dried over Na_2SO_4. The solvent was removed under reduced pressure to yield the product as a colourless oil, 1.25g, (94%). IRυ max: (NaCl) 1689 cm^{-1} (C=N). ^{1}H NMR (400MHz, $CDCl_3$): δ 0.11 (6H, s, $Si(CH_3)_2, C(CH_3)_3$), 0.93 (9H, s, $Si(CH_3)_2$ $C(CH_3)_3$), 3.81 (3H, s, -OMe), 6.88 (1H, d, J=8.52, H_5), 7.32 (1H, s, H_2), 7.48 (1H, d, J=2Hz, H_6). ^{13}C NMR (100 MHz, $CDCl_3$): δ -5.19, 17.86, 25.15 (TBDMS), 54.95 (OMe), 110.69 (C_5), 119.31 (C_2), 125.90 (C_6), 129.66 (C_1), 145.02 (C_3), 156.07 (C_4), 190.27 (CHO). HRMS: found 266.1349 [M^+]; $C_{14}H_{22}O_3Si$ requires 266.1338.

General method for Preparation of 3-chloroazetidin-2-ones and 3,3-dichloroazetidin-2-ones

To a stirring, refluxing solution of the imine (5 mmol) and triethylamine (6 mmol) in anhydrous dichloromethane (40 mL), a solution of chloro or dichloroacetyl chloride (6 mmol) in anhydrous dichloromethane (10 mL) was injected dropwise through a rubber septum over 45 minutes under nitrogen. The reaction was refluxed during the day (5 h) and at room temperature overnight, continuously under nitrogen. The reaction was transferred to a separating funnel and washed with water (2×100 mL), with the organic layer being retained each time. The reaction was dried over anhydrous sodium sulfate before the solvent was removed under reduced

pressure. The crude product was purified by flash chromatography over silica gel eluted with 4:1 *n*-hexane: ethyl acetate as eluent.

3-Chloro-4-(4-ethoxyphenyl)-1-(3,4,5-trimethoxyphenyl)azetidin-2-one (10f) preparation as described in the general method above from imine **9f** and chloroacetyl chloride**,** Yield: 6.6 %, 130 mg, brown oil [1]. IR (NaCl, film) ν_{max}: 2605, 2497, 1763 (C=O, β-lactam), 1568, 1508, 1240, 1128 cm^{-1}. ^{1}H NMR (400MHz, $CDCl_3$): δ 1.28 (t, J = 7.28 Hz, 3H, $OCH_2C\underline{H_3}$), 3.72 (s, 6H, OCH_3), 3.78 (s, 3H, OCH_3), 4.05 (q, J = 7.04 Hz, 2H, $OC\underline{H_2}CH_3$), 4.63 (d, J = 2.00 Hz, 1H, H_4), 4.94 (br s, 1H, H_3), 6.55 (s, 2H, H_2' H_6'), 6.95 (d, J = 8.52 Hz, 2H, H_3'' H_5''), 7.32 (d, J = 8.52 Hz, 2H, H_2'' H_6''). ^{13}C NMR (100MHz, $CDCl_3$): δ 14.28 ($OCH_2\underline{C}H_3$), 55.59, 60.51 (OCH_3), 62.74 (C_3), 63.18 ($O\underline{C}H_2CH_3$), 65.71 (C_4), 94.77 (C_2', C_6'), 114.86 (C_3'', C_5''), 126.13 (C_4'), 127.08 (C_2'', C_6''), 132.53 (C_1''), 134.59 (C_1'), 153.07 (C_3', C_5'), 159.44 (C_4''), 160.31 (C_2). HRMS: found 414.1100 [M+Na]$^+$; $C_{20}H_{22}{}^{35}ClNO_5Na$ requires 414.1084.

3-Chloro-4-(4-(methylthio)phenyl)-1-(3,4,5-trimethoxyphenyl)azetidin-2-one (10i): was synthesised following the general method above from imine **9i** and chloroacetyl chloride to afford the desired product as an orange powder; yield: 57%, Mp: 102 – 104 °C, [1] (HPLC): 97%. IRν_{max} (ATR): 1747.2 (C=O) cm^{-1}. ^{1}H NMR (400 MHz, $CDCl_3$): δ 2.47 (s, 3 H, SCH_3), 3.69 (s, 6 H, OCH_3), 3.75 (s, 3 H, OCH_3), 4.58 (d, *J* = 1.22 Hz, 1 H, H4), 4.91 (d, *J* = 1.22 Hz, 1 H, H3), 6.50 (s, 2 H, ArH), 7.27 - 7.82 (m, 4 H, ArH). ^{13}C NMR (100 MHz, $CDCl_3$): δ 15.35, 56.07, 60.91, 63.07, 66.01, 95.29, 126.54, 126.84, 131.34, 132.80, 135.24, 140.77, 153.57, 160.50 (C_2, C=O). HRMS: found 416.0688 [M + Na]$^+$; $C_{19}H_{20}{}^{35}ClNNaO_4S$ requires 416.0699.

3,3-Dichloro-1-(3,4,5-trimethoxyphenyl)-4-(4-methoxyphenyl)azetidin-2-one (11e) preparation as described in the general method above from imine **9e** and dichloroacetyl chloride, Yield: 28.3 %, 583 mg, yellow oil, (HPLC): 94.1% [1]. IR (NaCl, film) ν_{max}: 2942, 2683, 1777 (C=O, β-lactam), 1598, 1507, 1255, 1126 cm^{-1}. ^{1}H NMR (400MHz, $CDCl_3$): δ 3.73 (s, 6H, OCH_3), 3.80 (s, 3H, OCH_3), 3.85 (s, 3H, OCH_3), 5.45 (s, 1H, H_4), 6.56 (s, 2H, H_2' H_6'), 6.97 (d, J = 8.52 Hz, 2H, H_3'' H_5''), 7.29 (d, J = 8.56 Hz, 2H, H_2'' H_6''). ^{13}C NMR (100MHz, $CDCl_3$): δ 54.89, 55.68, 60.52 (OCH_3), 73.61 (C_4), 83.80 (C_3), 95.41 (C_2', C_6'), 113.89 (C_3'', C_5''), 122.90 (C_4'), 128.80 (C_2'', C_6''), 131.45 (C_1''), 135.19 (C_1'), 153.17 (C_3', C_5'), 157.96 (C_4''), 160.36 (C_2). HRMS: found 412.0715 [M+H]$^+$; $C_{19}H_{20}{}^{35}Cl_2NO_5$ requires 412.0719.

3,3-Dichloro-4-(4-ethoxyphenyl)-1-(3,4,5-trimethoxyphenyl)azetidin-2-one (11f) preparation as described in the general method above from imine **9f** and dichloroacetyl chloride, Yield: 28.9 %, 615 mg, brown oil [1]. IR (NaCl, film) ν_{max}: 2605, 2498, 1778 (C=O, β-lactam), 1600, 1507, 1241, 1127 cm^{-1}. ^{1}H NMR (400MHz, $CDCl_3$): δ 1.44 (t, J = 7.02Hz, 3H, $OCH_2C\underline{H_3}$), 3.73 (s, 6H, OCH_3), 3.80 (s, 3H, OCH_3), 4.06 (q, J = 7.00 Hz, 2H, $OC\underline{H_2}CH_3$), 5.44 (s, 1H, H_4), 6.56 (s, 2H, $H_{2'}$ $H_{6'}$), 6.96 (d, J = 8.76 Hz, 2H, $H_{3''}$ $H_{5''}$), 7.27 (d, J = 8.80 Hz, 2H, $H_{2''}$ $H_{6''}$). ^{13}C NMR (100MHz, $CDCl_3$): δ 14.29 ($OCH_2\underline{C}H_3$), 55.66, 60.52 (OCH_3), 63.13 ($O\underline{C}H_2CH_3$), 73.65 (C_4), 83.80 (C_3), 95.40 ($C_{2'}$, $C_{6'}$), 114.33 ($C_{3''}$, $C_{5''}$), 122.68 ($C_{4'}$), 128.77 ($C_{2''}$, $C_{6''}$), 131.47 ($C_{1''}$), 135.15 ($C_{1'}$), 153.15 ($C_{3'}$, $C_{5'}$), 157.98 ($C_{4''}$), 159.78 (C_2) HRMS: found 424.0724 $[M-H]^-$, $C_{20}H_{20}{}^{35}Cl_2NO_5$ requires 424.0719.

3,3-Dichloro-4-(4-(methylthio)phenyl)-1-(3,4,5-trimethoxyphenyl)azetidin-2-one (11i): was synthesised following the general method above from imine **9i** and dichloroacetyl chloride to afford the product as a brown powder; yield: 63%, Mp: 118 – 119 °C, [1] (HPLC): 98.9%. IRν_{max} (ATR): 1770.2 (C=O) cm^{-1}. ^{1}H NMR (400 MHz, $CDCl_3$): δ 2.48 (s, 3 H, SCH_3), 3.70 (s, 6 H, OCH_3), 3.77 (s, 3 H, OCH_3), 5.42 (s, 1 H, H4), 6.52 (s, 2 H, ArH), 7.20 - 7.23 (m, 2 H, ArH), 7.25 - 7.28 (m, 2 H, ArH). ^{13}C NMR (100 MHz, $CDCl_3$): δ 15.10, 56.16, 60.94, 73.96, 84.00, 95.88, 126.06, 128.13, 131.75, 135.80, 141.38, 153.65, 158.24 (C_2, C=O). HRMS: found 450.0320 $[M + Na]^+$; $C_{19}H_{19}{}^{35}Cl_2NNaO_4S$ requires 450.0310.

3-Chloro-4-(3-chloro-4-methoxyphenyl)-1-(3,4,5-trimethoxyphenyl) azetidin-2-one (10o): was synthesised using the general above from imine **9r**, and chloroacetyl chloride to afford the product as an oil; yield: 42%, purity (HPLC): 99%, [2]. IR νmax (ATR): 1758.6 (C=O) cm^{-1}. ^{1}H NMR (400 MHz, $CDCl_3$): δ 3.76 (s, 6 H, OCH_3), 3.81 (s, 3 H, OCH_3), 3.95 (s, 3 H, OCH_3), 4.63 (d, J = 2.20 Hz, 1 H, H4), 4.93 (d, J = 1.47 Hz, 1 H, H3), 6.55 (s, 2 H, ArH), 6.99 (d, J = 8.07 Hz, 1 H, ArH), 7.27 (d, J = 2.20 Hz, 1 H, ArH), 7.45 (d, J = 2.20 Hz, 1 H, ArH). ^{13}C NMR (100 MHz, $CDCl_3$): δ 56.12, 60.93, 63.07, 65.39, 95.29, 112.60, 123.67, 125.56, 128.04, 132.68, 153.62, 155.90, 160.36 (C2, C=O). HRMS: calculated for $C_{19}H_{19}{}^{35}Cl_2NaNO_5$ $[M+Na]^+$ 434.0538; found 434.0521.

3,3-Dichloro-4-(4-methoxy-3-methylphenyl)-1-(3,4,5-trimethoxyphenyl)azetidin-2-one (11o): was synthesised using the general procedure above from imine **9p** and dichloroacetyl chloride to afford the product as yellow solid; yield: 50%, Mp: 150 °C, purity (HPLC): 95%,

[2]. IR ν max (ATR): 1766.4 (C=O) cm^{-1}. ^{1}H NMR (400 MHz, $CDCl_3$): δ ppm 2.17 (s, 3 H, CH_3), 3.67 (s, 6 H, OCH_3), 3.73 (s, 3 H, OCH_3), 3.79 (s, 3 H, OCH_3), 5.37 (s, 1 H, H4), 6.52 (s, 2 H, ArH), 6.80 (d, J = 8.29 Hz, 1 H, ArH), 7.01 - 7.16 (m, 2 H, ArH). ^{13}C NMR (100 MHz, $CDCl_3$): δ ppm 16.31, 55.30, 56.09, 60.86, 84.31, 95.96, 109.87, 122.76, 126.61, 127.32, 129.88, 131.94, 135.65, 153.57, 158.41, 162.11 (C2, C=O). HRMS: calculated for $C_{20}H_{22}{}^{35}Cl_2NO_5$ $[M + H]^+$ 426.0875; found 426.0863.

Table S1: Tier-1 Profiling Screen of Selected 3-chloroazetidinones, 3,3-dichloroazetidinones and related compounds[a]

Compound	ID	ADMET Solubility[b]	ADMET Solubility Level[c]	ADMET BBB[d]	ADMET BBB Level[e]	ADMET CYP2D6 Prediction[f]	ADMET Hepatotoxic Prediction[g]
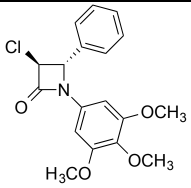	**10a**	-4.4330	2	0.086000	1	false	true
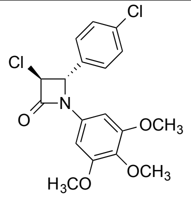	**10b**	-5.0650	2	0.29100	1	false	true
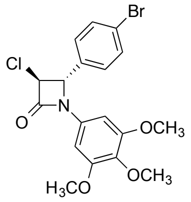	**10c**	-5.1380	2	0.31700	1	false	true
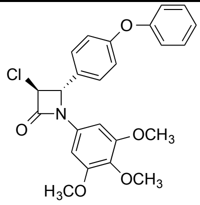	**10g**	-5.6770	2	0.42700	1	false	true
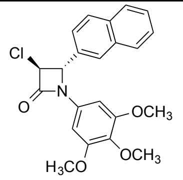	**10k**	-5.7460	2	0.36700	1	false	true
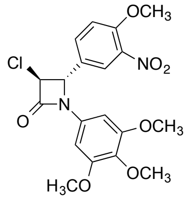	**10m**	-4.3750	2	-0.77100	3	false	true
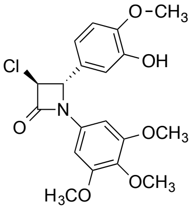	**10n**	-4.0300	2	-0.46400	2	false	true

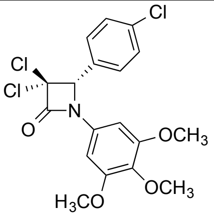	**11b**	-5.6180	2	0.44600	1	false	true
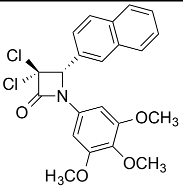	**11k**	-6.2750	1	0.52100	1	false	true
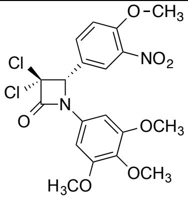	**11m**	-4.9150	2		4	false	true
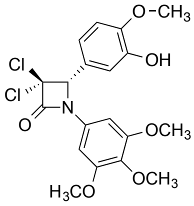	**11n**	-4.6590	2	-0.31000	2	false	true
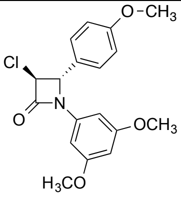	**14a**	-4.4060	2	0.086000	1	false	true
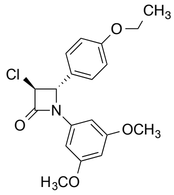	**14b**	-4.6100	2	0.19400	1	false	true
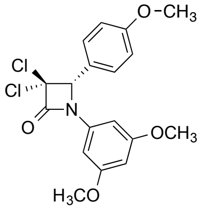	**15a**	-4.9640	2	0.24100	1	false	true
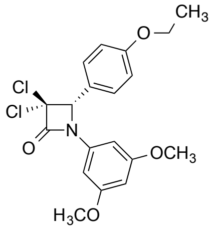	**15b**	-5.1640	2	0.34800	1	false	true

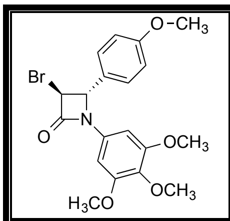	**16a**	-4.4950	2	-0.016000	2	false	true
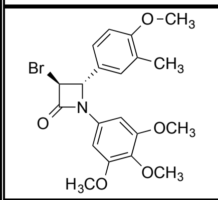	**16e**	-4.9760	2	0.13500	1	false	true
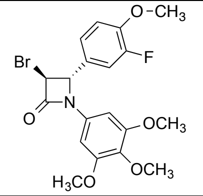	**16f**	-4.7310	2	0.048000	1	false	true
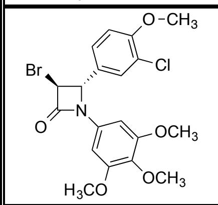	**16g**	-5.1320	2	0.19000	1	false	true
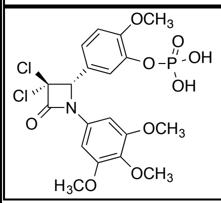	**18**	-4.8040	2		4	false	true

[a]Calculated using Pipeline Pilot Professional (v8.5.0.200) BIOVIA, Dassault Systèmes
[b]ADMET Solubility: Log of the water solubility at 25 °C (LogSw)(mol/L)
[c]ADMET Solubility Level: Ranking of the solubility values into the following classes: 0: Extremely Low; 1: Very Low; 2: Low; 3: Good; 4: Optimal; 5: Very Soluble
[d]ADMET BBB: Predicts the blood brain barrier penetration of a molecule, defined as the ratio of the concentrations of solute (compound) on the both sides of the membrane after oral administration.
[e]ADMET Blood Brain Barrier Absorption (BBB) Level: Ranking of LogBBB values into one of the following levels: 0: Very High; 1: High; 2: Medium; 3: Low; 4: Undefined (molecule is outside the confidence area of the regression model used to calculate LogBB)
[f]CYP2D6 inhibitor prediction
[g]Human hepatoxicity prediction

Table S2: ADMET and Lipinski Properties for Selected 3-chloroazetidinones, 3,3-dichloroazetidinones and related compounds[a]

Compound	ID	ADMET Absorption Level[b]	ADMET EXT PPB Prediction[c]	ALogP[d]	Molecular Weight	Num HBA	Num HBD	Num RotBonds	Molecular Volume	Molecular Polar Surface Area
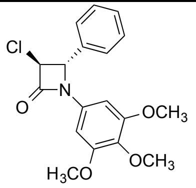	**10a**	0	true	3.205	347.79	4	0	5	226.37	48
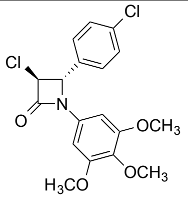	**10b**	0	true	3.870	382.24	4	0	5	247.64	48
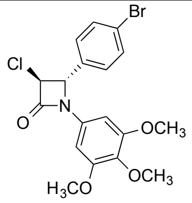	**10c**	0	true	3.954	426.69	4	0	5	257.24	48
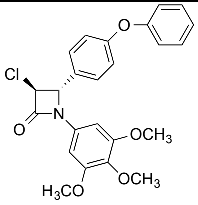	**10g**	0	true	4.766	439.89	5	0	7	285.03	57.230
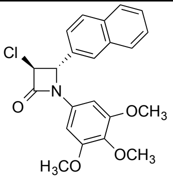	**10k**	0	true	4.660	397.85	4	0	5	259.65	48
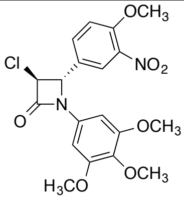	**10m**	0	true	3.083	422.82	7	0	7	270.62	103.04
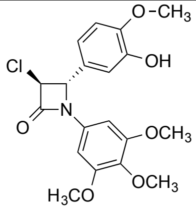	**10n**	0	true	2.670	393.82	6	1	6	255.19	77.460

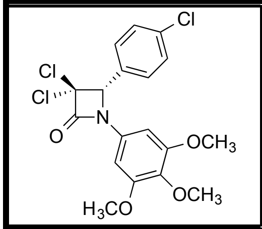	**11b**	0	true	4.370	416.68	4	0	5	262.39	48
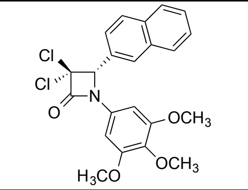	**11k**	0	true	5.840	432.30	4	0	5	275.77	48
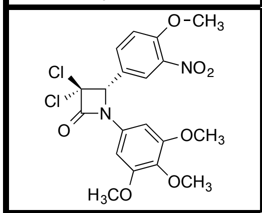	**11m**	0	true	3.584	457.26	7	0	7	284	103.04
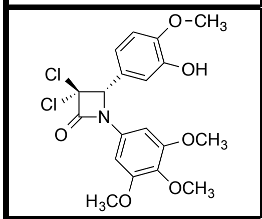	**11n**	0	true	3.850	428.26	6	1	6	270.62	77.460
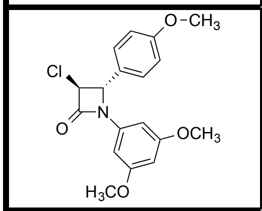	**14a**	0	true	3.205	347.79	4	0	5	231.86	48
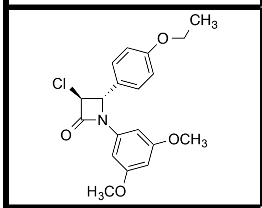	**14b**	0	true	3.554	361.82	4	0	6	238.04	48
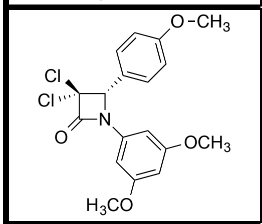	**15a**	0	true	3.706	382.24	4	0	5	247.98	48
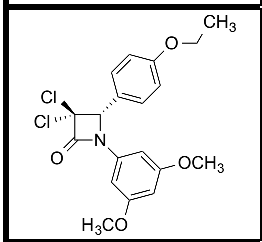	**15b**	0	true	4.054	396.26	4	0	6	255.87	48

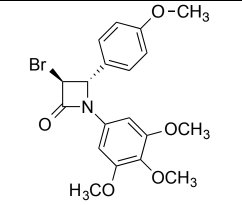	**16a**	0	true	3.540	422.27	5	0	6	259.30	57.230
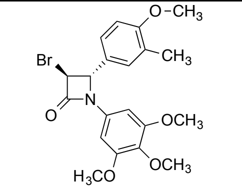	**16e**	0	true	3.820	436.30	5	0	6	278.17	57.230
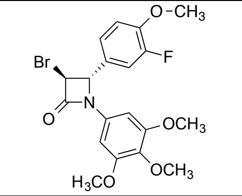	**16f**	0	true	3.850	440.26	5	0	6	265.82	57.230
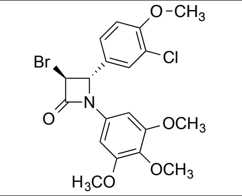	**16g**	0	true	3.998	456.71	5	0	6	278.51	57.230
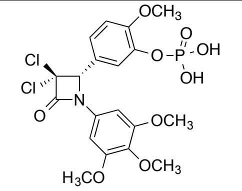	**18**	1	true	3.193	508.24	9	2	8	309.04	133.80

[a]Calculated using Pipeline Pilot Professional (v8.5.0.200) BIOVIA, Dassault Systèmes
[b]ADMET Calculates ADMET Passive Intestinal Absorption properties. Accelrys passive intestinal absorption model. Absorption Level: Ranking of the molecule into one of the following levels: 0: Good; 1: Moderate; 2: Poor; 3: Very Poor
[c]ADMET Plasma Protein Binding (PPB) Prediction: If true, the compound is predicted to be a binder (>=90%). Otherwise, it is predicted to be a weak or nonbinder(<90%).
[d] ChemBioDraw Ultra 13.0.2.3020

Table S3: Standard COMPARE analysis of compound **10e**, **11n** and **16d**[a]

**Rank**	**10e (76240)**	*r*	**11n (76241)**	*r*	**16d (792961)**	*r*
	**Based on GI_{50} mean graph**		**Based on GI_{50} mean graph**		**Based on GI_{50} mean graph**	
1	Vincristine sulfate hiConc:-3.0	0.56	Vincristine sulfate hiConc:-3.0	0.53	Tiazofurin hiConc:-2.0	0.53
2	Maytansine hiConc:-4.0	0.54	Maytansine hiConc:-4.0	0.48	Maytansine hiConc:-2.0	0.51
3	Maytansine hiConc:-7.0	0.50	Glycoxalic acid hiConc:-2.9	0.46	Methotrexate hiConc:-7.0	0.49
4	Vinblastine sulfate hiConc:-4.0	0.49	Vinblastine sulfate hiConc:-4.0	0.43	Brequinar hiConc:-4.0	0.45
5	Maytansine hiConc:-3.0	0.48	Maytansine hiConc:-3.6	0.42	Macbecin II hiConc:-6.0	0.45
	**Based on TGI mean graph**[b]		**Based on TGI mean graph**		**Based on TGI mean graph**	
1	Maytansine hiConc:-3.6	0.72	Vincristine sulfate hiConc:-3.0	0.61	Ftorafur hiConc:-2.3	0.43
2	Maytansine hiConc:-4.0	0.71	Vinblastine sulfate hiConc:-5.6	0.57	Rifamycin SV hiConc:-3.0	0.42
3	Vincristine sulfate hiConc:-3.0	0.69	Maytansine hiConc:-4.0	0.57	Pibenzimol hydrochloride hiConc:-2.7	0.42
4	Vinblastine sulfate hiConc:-5.6	0.68	Vinblastine sulfate hiConc:- 4.0	0.56	Pibenzimol hydrochloride hiConc:-4.0	0.40
5	Vincristine sulfate hiConc:-5.0	0.65	Vincristine sulfate hiConc:-5.0	0.56	Fluorodopan hiConc:-2.9	0.40

[a]The target set was the standard agent database and the target set endpoints were selected to be equal to the seed end points. Standard COMPARE analysis was performed. Correlation values (r) are Pearson correlation coefficients. Vinblastine sulfate and Maytansine appears at different concentrations as it has been tested by the NCI at multiple concentration ranges [b]**10e** Based on TGI Rhizoxin ranked 6, ***r***=0.61, Paclitaxel(Taxol) ranked 13, ***r***=0.49.

[https://dtp.cancer.gov (accessed 20/9/2021)].

Table S4: Cell cycle analysis of MCF-7 cells following treatment with **10n**[a].

		% of cells in different phases of cell cycle		
**Compound**	**Time point (h)**	**G_0-G_1**	**G_2/M**	**Sub-G_1**
**control**	24	49.66 ± 3	27.6 ±1	1.73±0.5
	48	50.00 ± 1	22.67 ±3	2.53±0.2
	72	50.66 ± 0.8	21.33±2	7.00±1
**50 nM**	24	19.66 ± 0.6	59.66±5	2.33±0.4
	48	13.50 ± 2	60.76±2	5.10±0.5
	72	11.00 ± 0.7	62.17±2	13.16±0.7
**500 nM**	24	8.40 ± 0.6	79.33±3	3.50±0.2
	48	8.06 ± 0.2	77.53±1	7.60±0.7
	72	8.46 ± 0.3	63.33±2	24.46±0.3

[a]Cell cycle analysis of MCF-7 cells treated without and with different concentrations of **10n**. Data were an average ± SD of three sets of experiments.

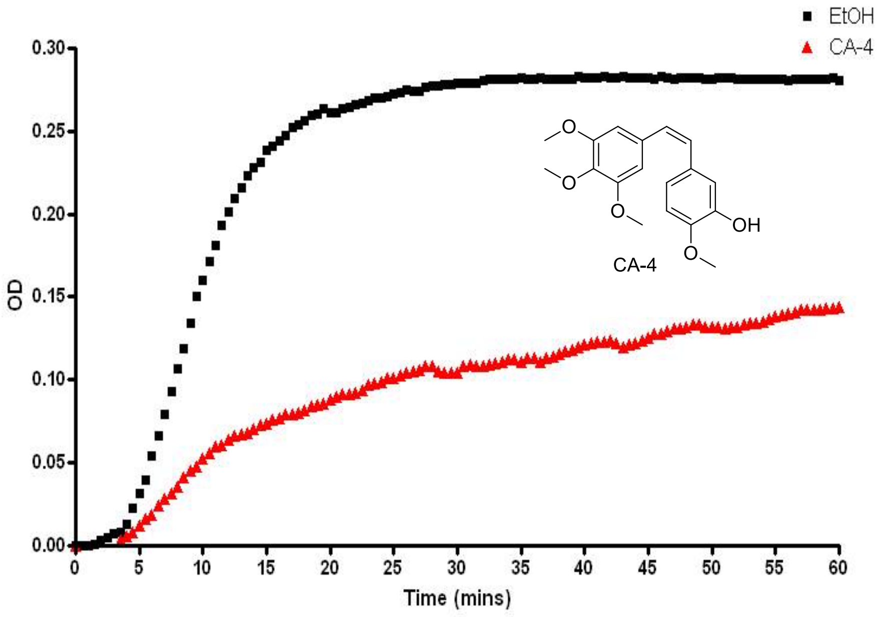

Figure S1: Effect of CA-4 on tubulin polymerization *in vitro*

Effect of CA-4 (10 µM), on tubulin polymerization *in vitro* ethanol (1% *v/v*) used as vehicle control. Purified bovine tubulin and GTP were mixed in a 96-well plate. The polymerization reaction was initiated by warming the solution from 4 °C to 37 °C. The effect on tubulin assembly was monitored in a Spectramax 340PC spectrophotometer at 340 nm at 30 s intervals for 60 min at 37 °C. Fold inhibition of tubulin polymerization was calculated using the v_{max} value for each reaction. The results represent the mean for three separate experiments.

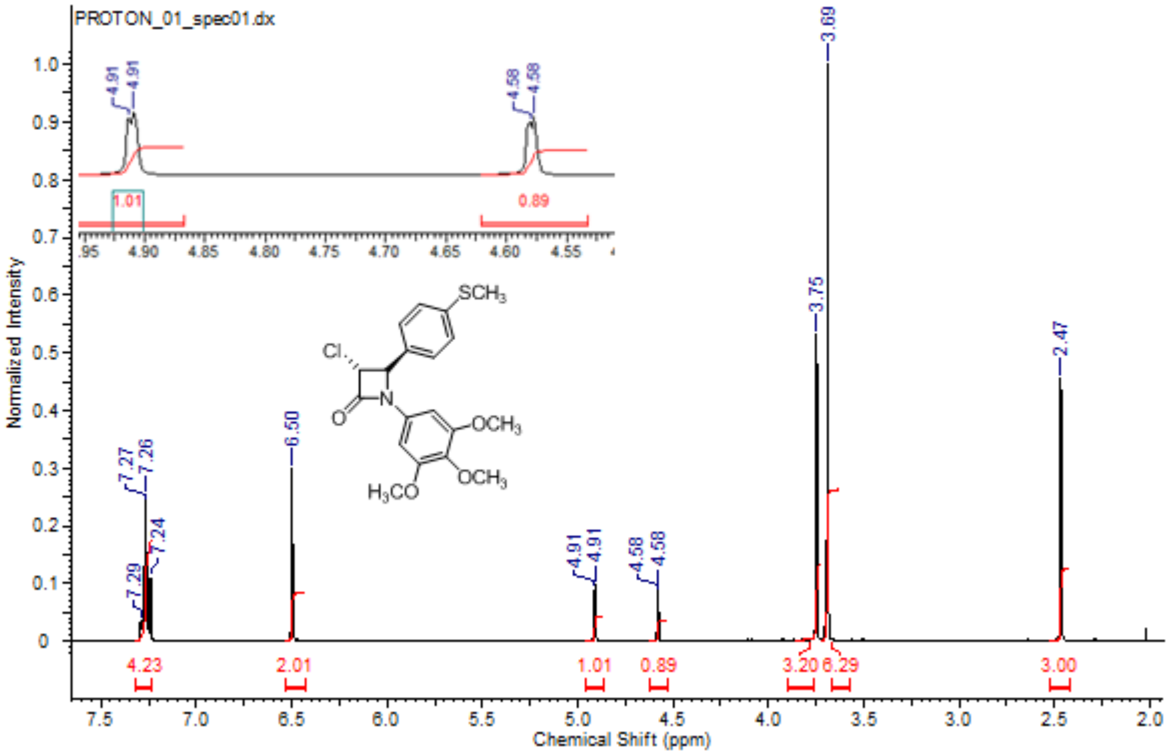

Figure S2 ^{1}H NMR spectrum of 3-chloro-β-lactam **10i**

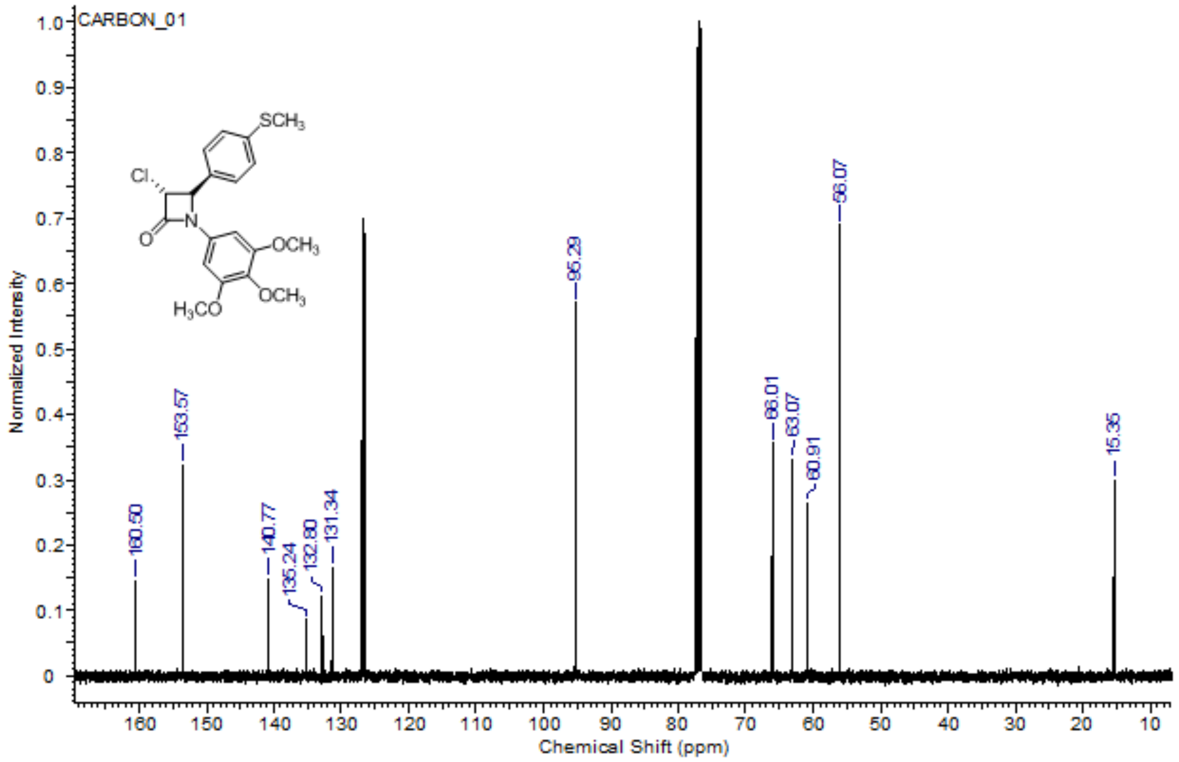

Figure S3: ^{13}C NMR spectrum of 3-chloro-β-lactam **10i**

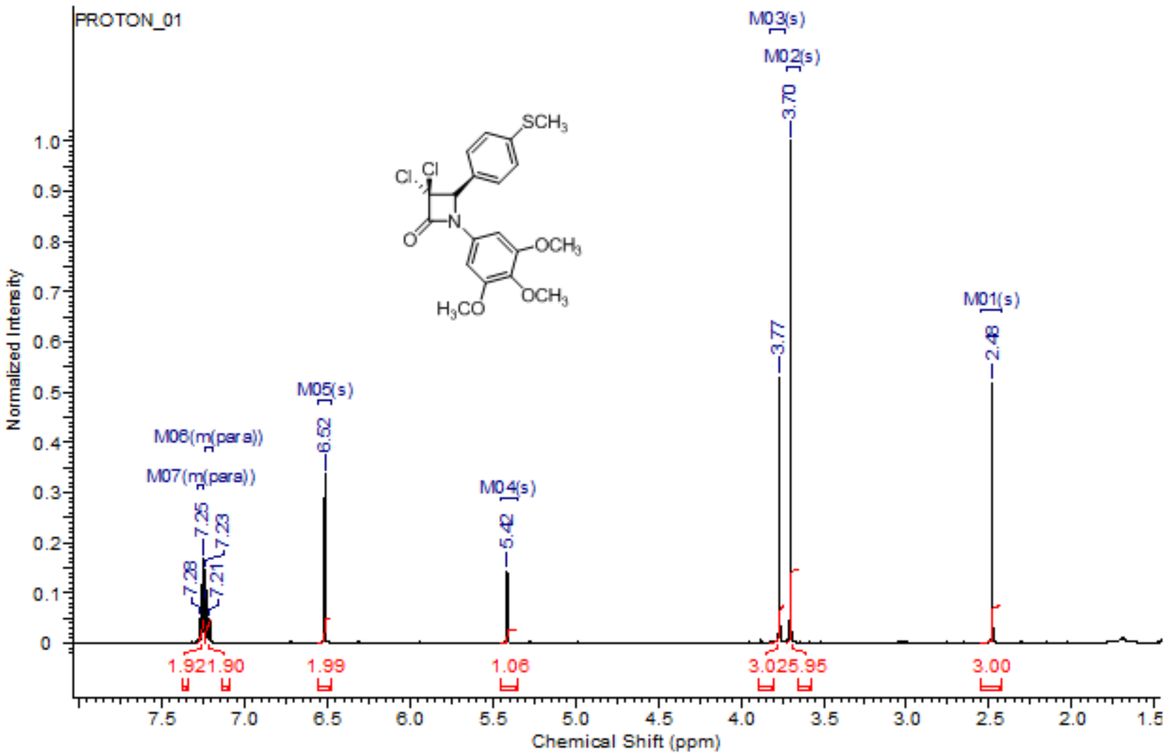

Figure S4: ^{1}H NMR spectrum of 3,3-dichloro-β-lactam **11i**

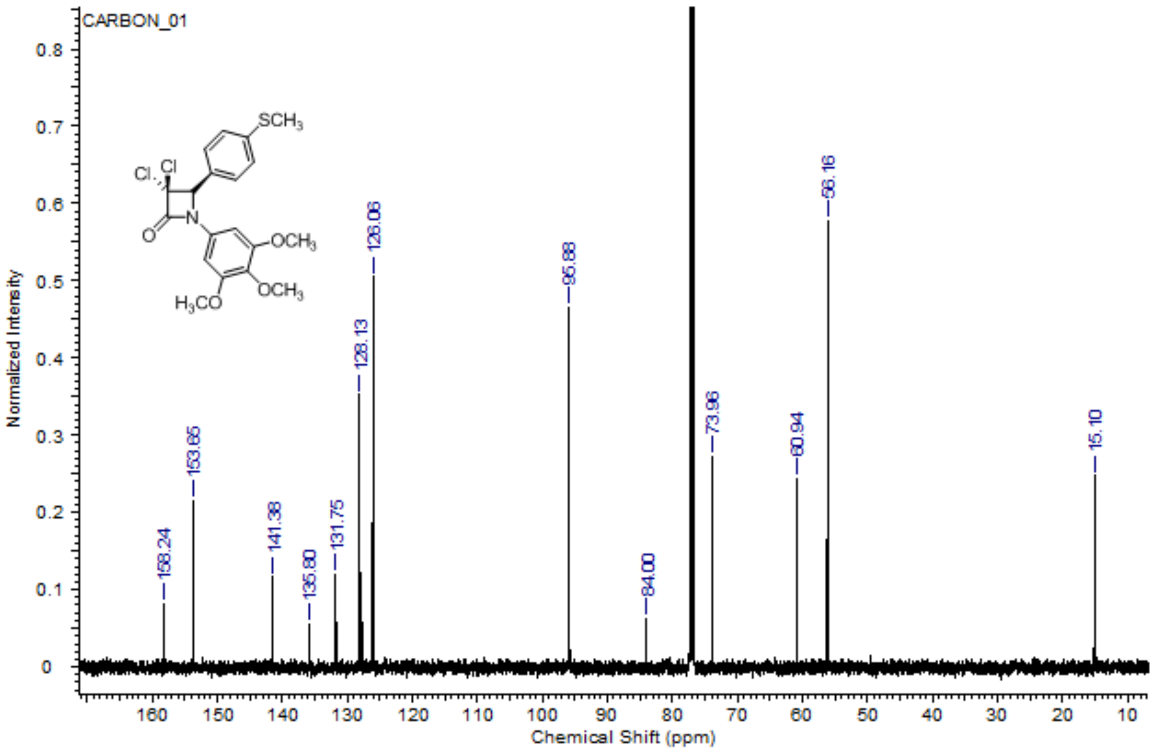

Figure S5: ^{13}C NMR spectrum of 3,3-dichloro-β-lactam **11i**

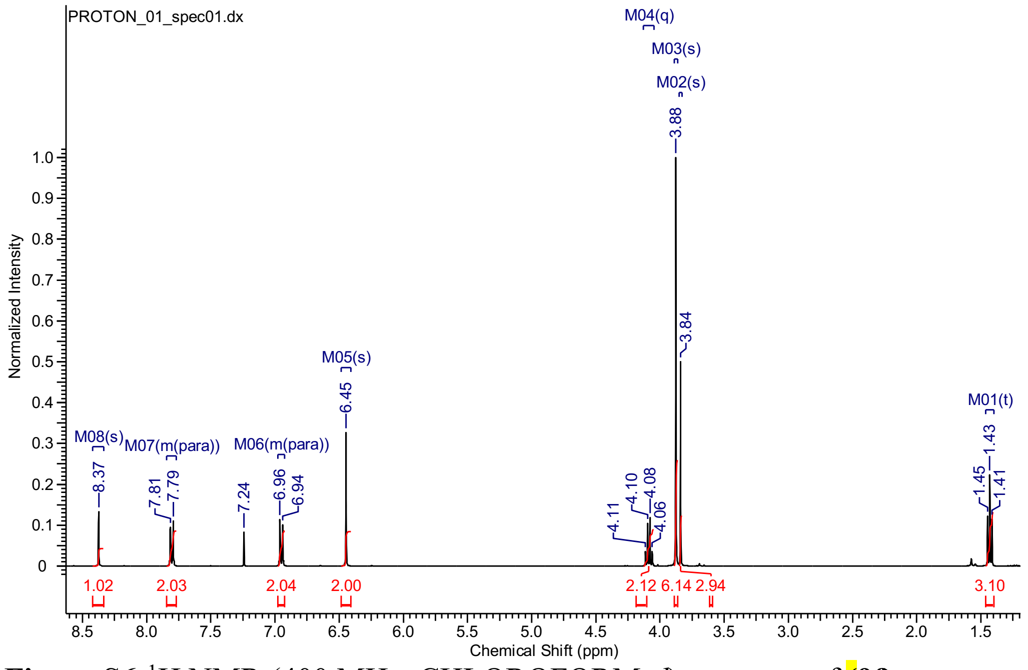

Figure S6 ^{1}H NMR (400 MHz, CHLOROFORM-*d*) spectrum of **(9f)**

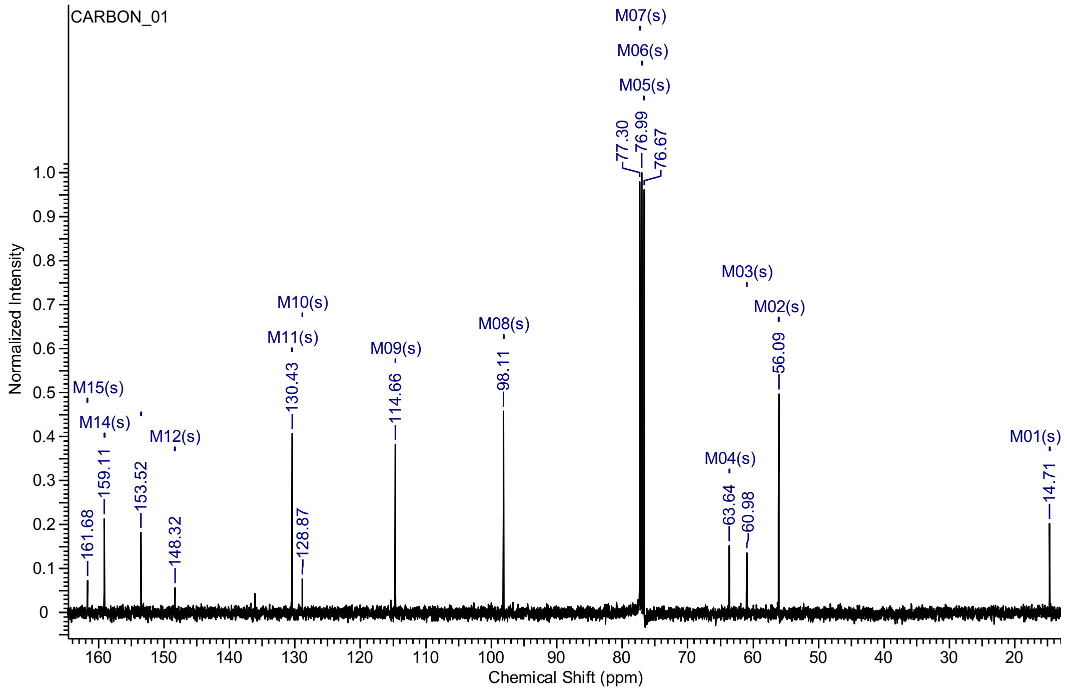

Figure S7: ^{13}C NMR (101 MHz, CHLOROFORM-*d*) spectrum of **(9f)**

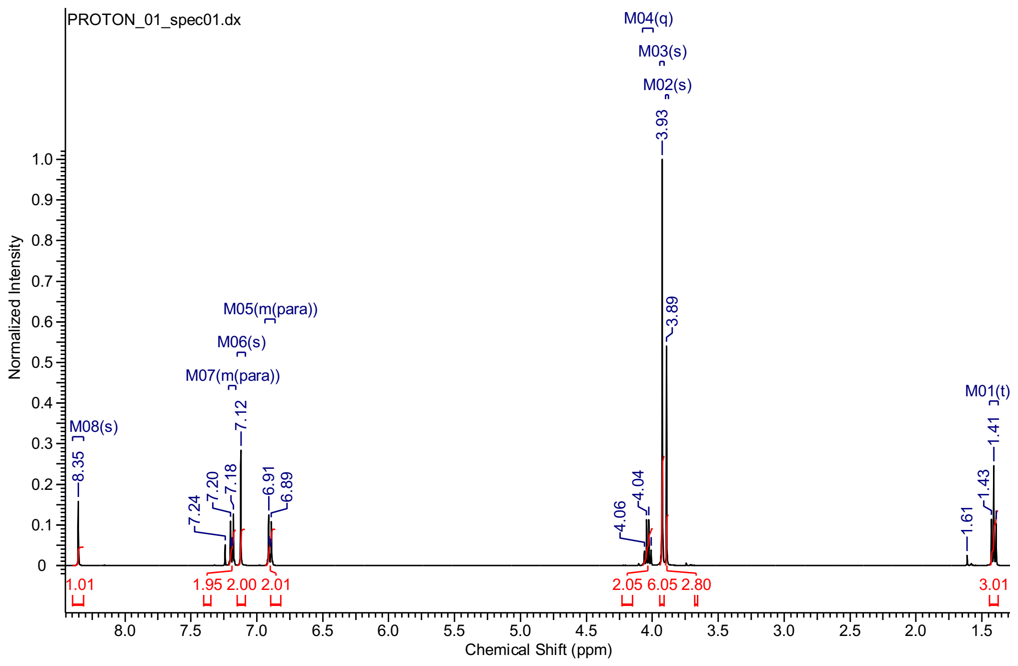

Figure S8: ^{1}H NMR (400 MHz, CHLOROFORM-*d*) spectrum of **(9u)**

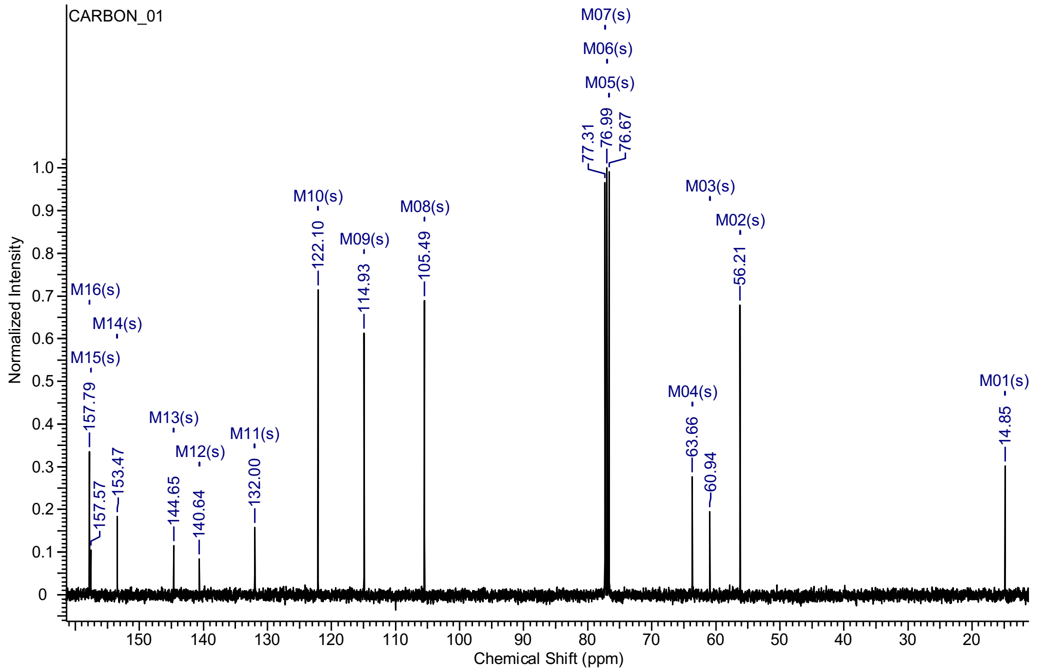

Figure S9: ^{13}C NMR (101 MHz, CHLOROFORM-*d*) spectrum of **(9u)**

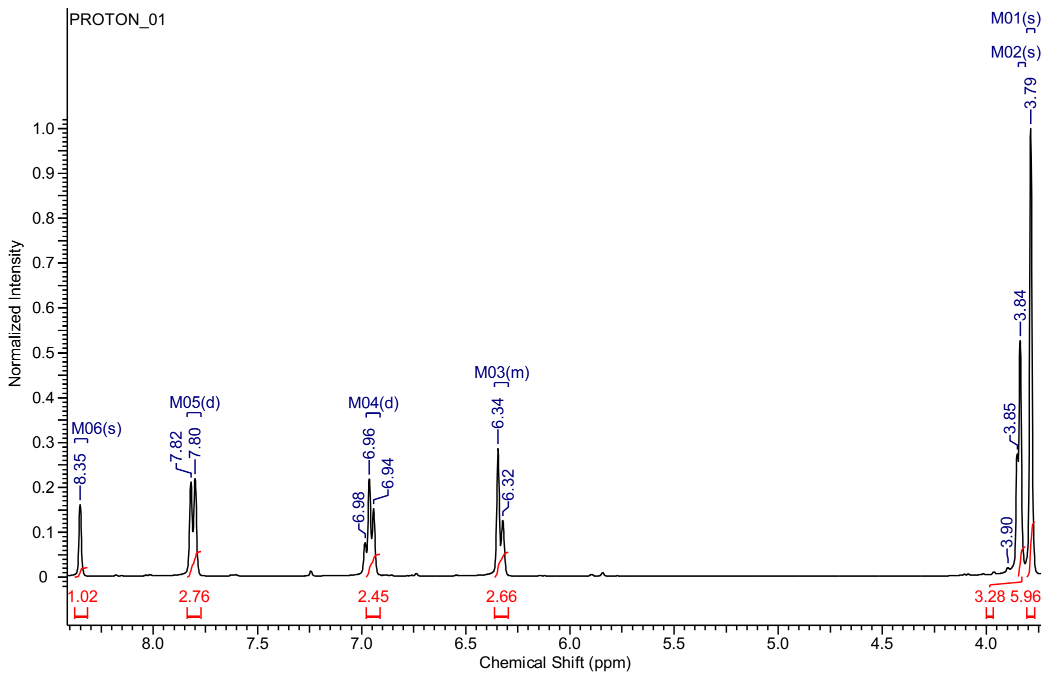

Figure S10: ^{1}H NMR (400 MHz, CHLOROFORM-*d*) spectrum of **(9w)**

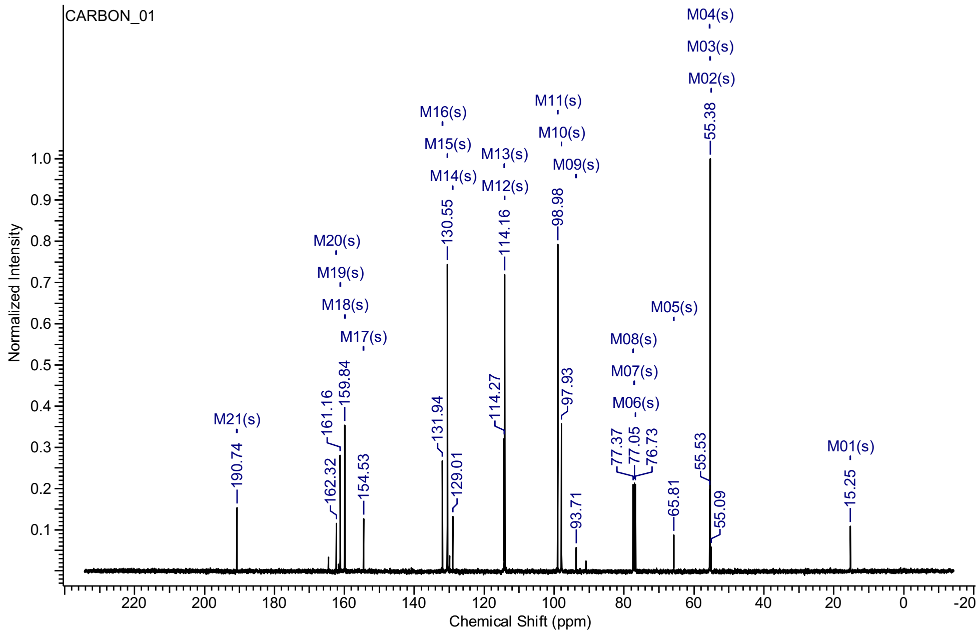

Figure S11: ^{13}C NMR (101 MHz, CHLOROFORM-*d*) spectrum of **(9w)**

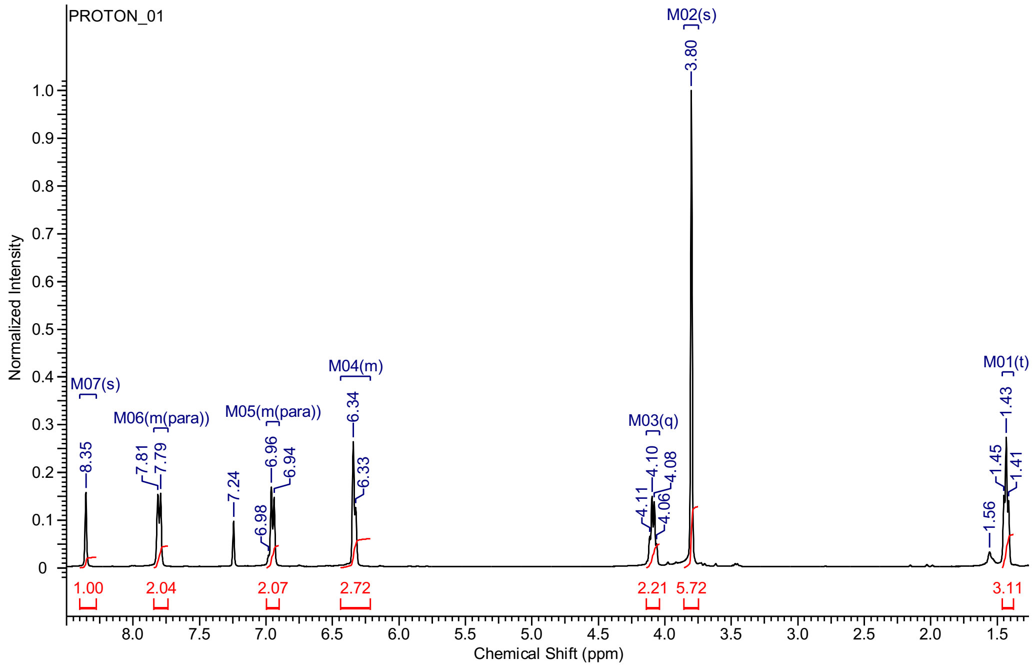

Figure S12: ^{1}H NMR (400 MHz, CHLOROFORM-*d*) spectrum of **(9x)**

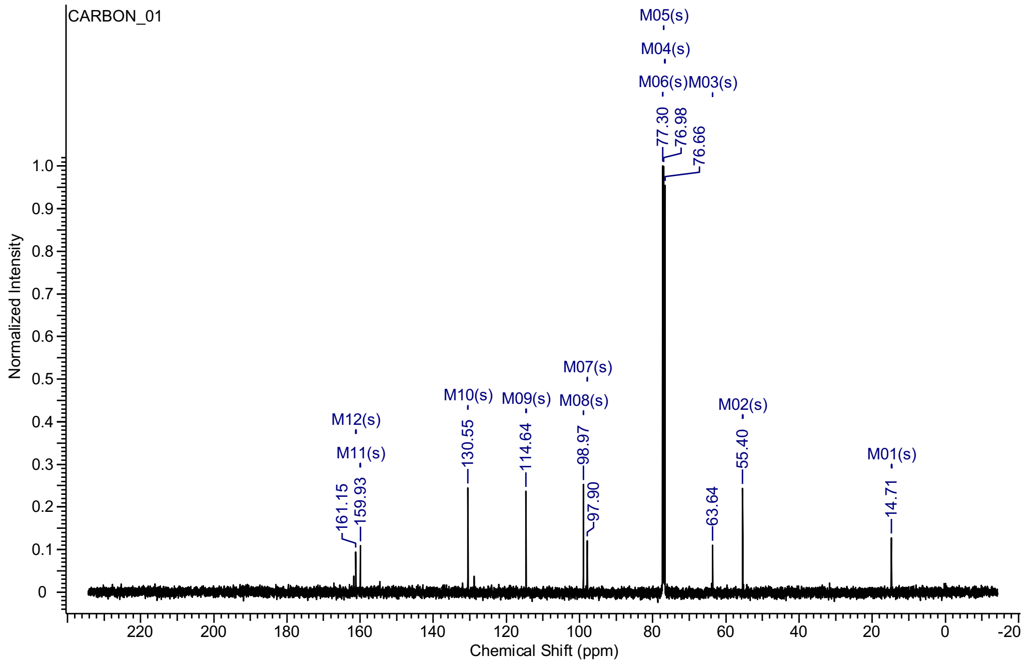

Figure S13: ^{13}C NMR (101 MHz, CHLOROFORM-*d*) spectrum of **(9x)**

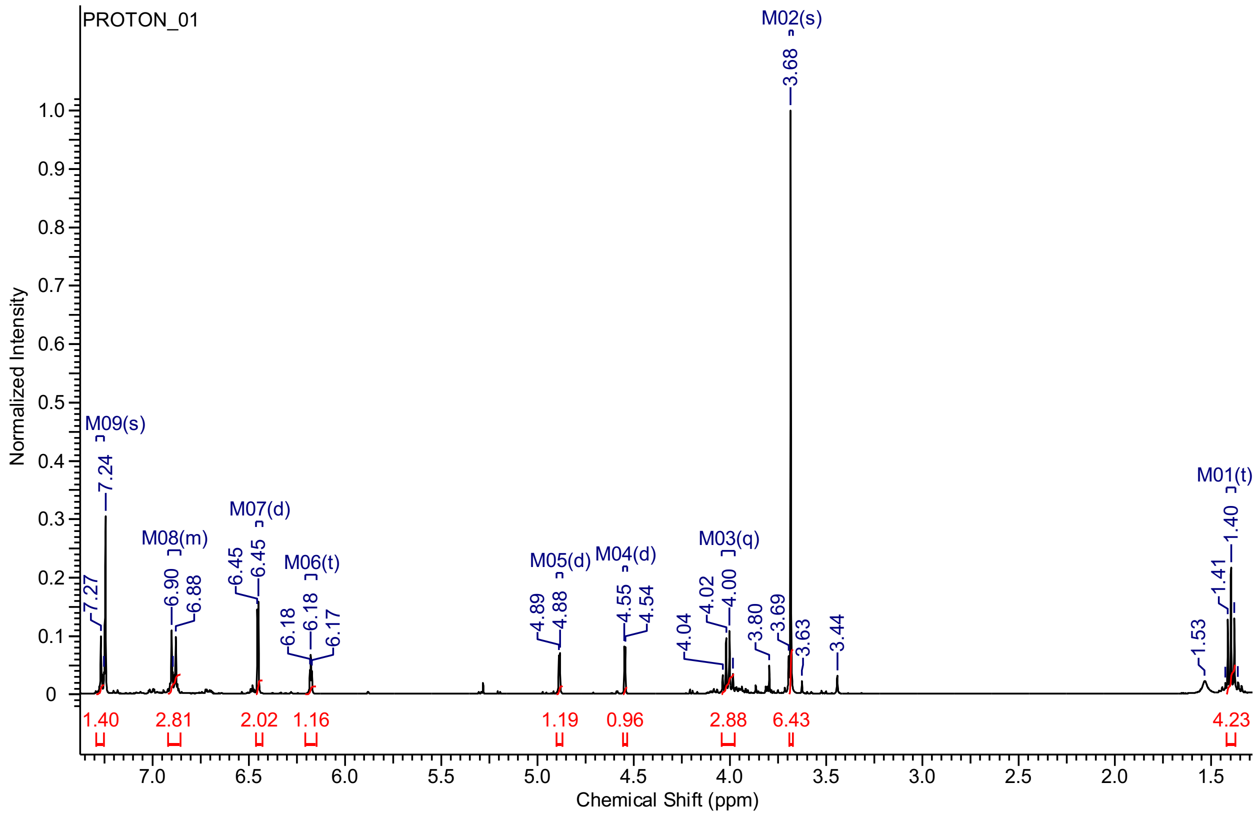

Figure S14: ^{1}H NMR (400 MHz, CHLOROFORM-*d*) spectrum of **(14b)**

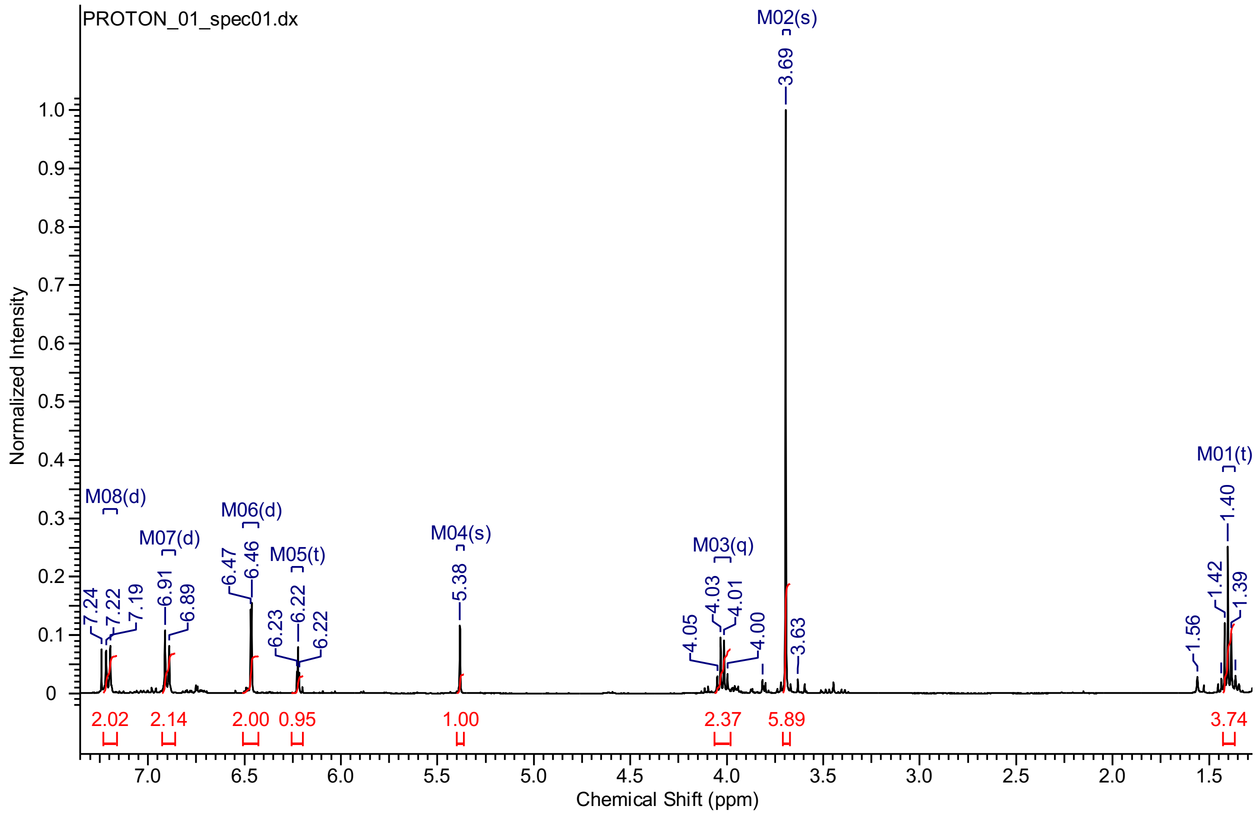

Figure S15: ^{1}H NMR (400 MHz, CHLOROFORM-*d*) spectrum of **(15b)**

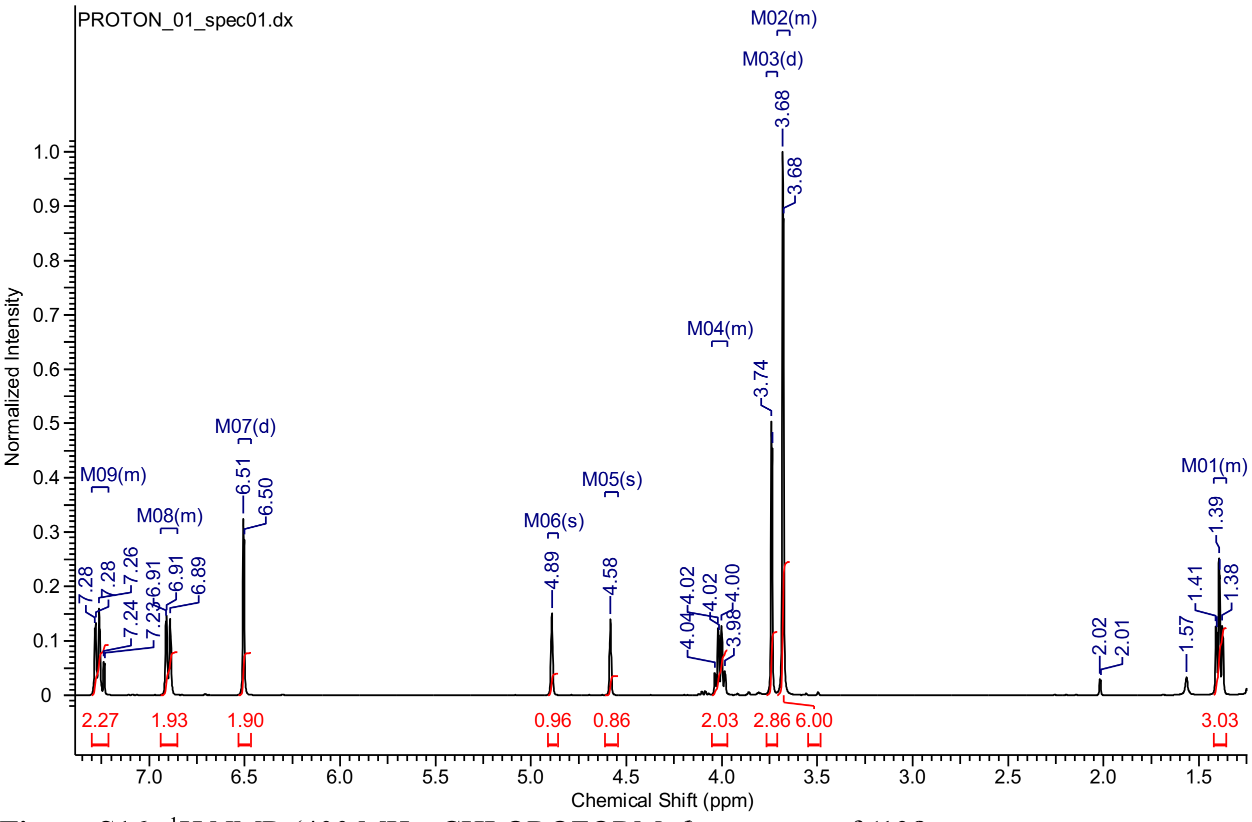

Figure S16: ^{1}H NMR (400 MHz, CHLOROFORM-*d*) spectrum of **(10f)**

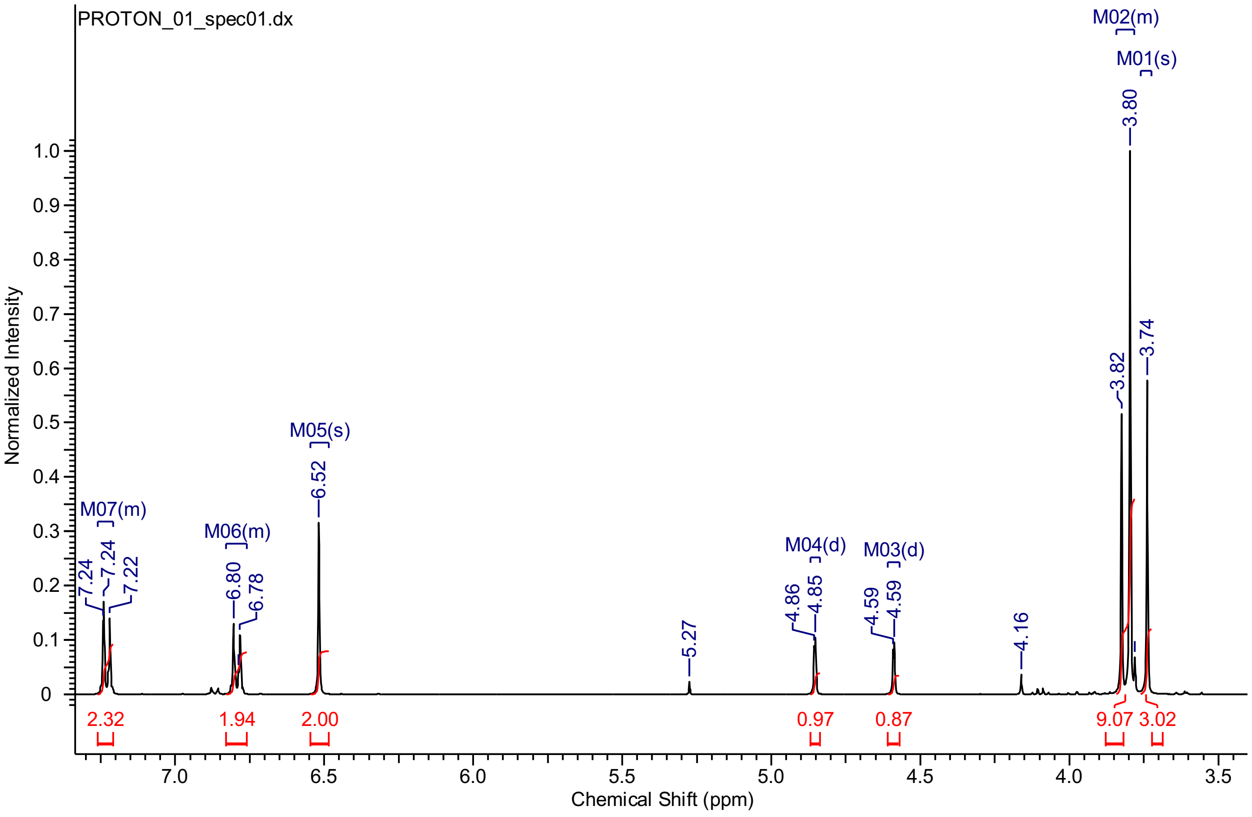

Figure S17: ^{1}H NMR (400 MHz, CHLOROFORM-*d*) spectrum of **(12a)**

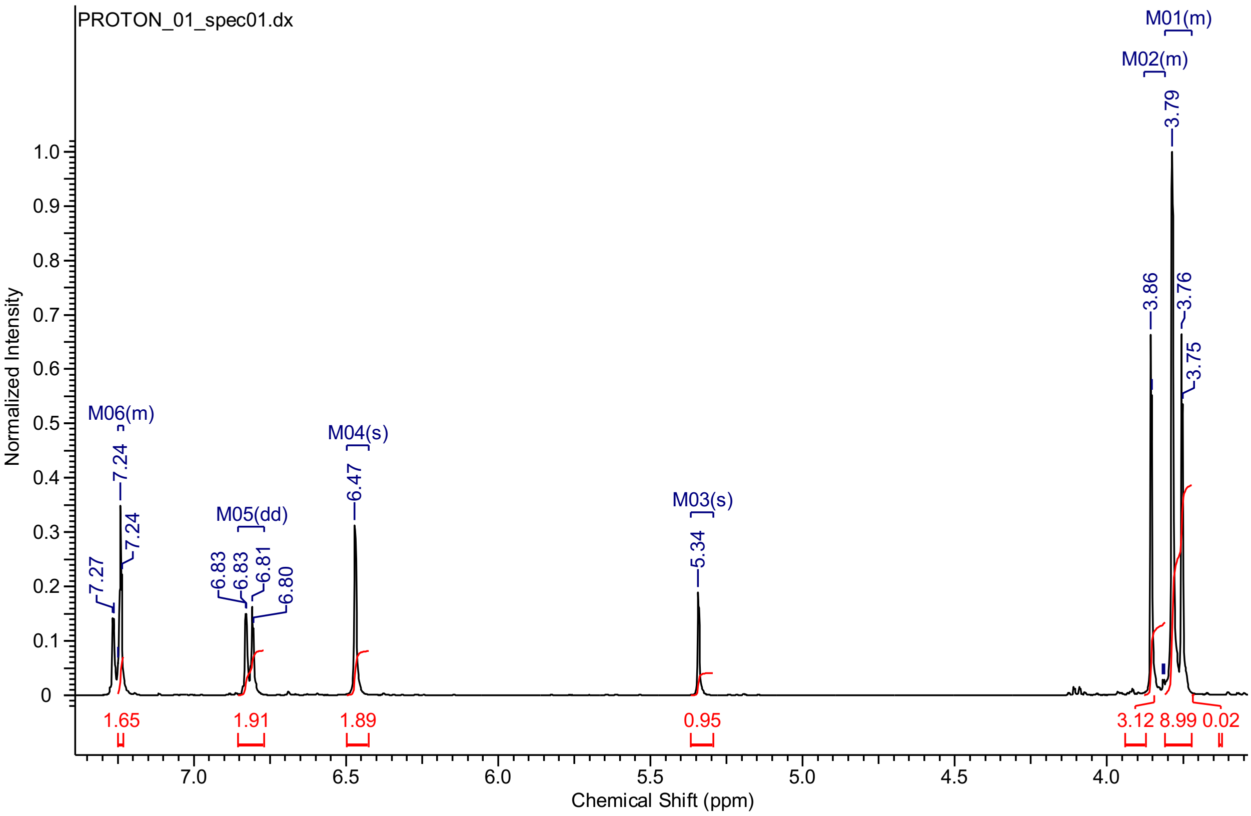

Figure S18: ^{1}H NMR (400 MHz, CHLOROFORM-*d*) spectrum of **(13a)**

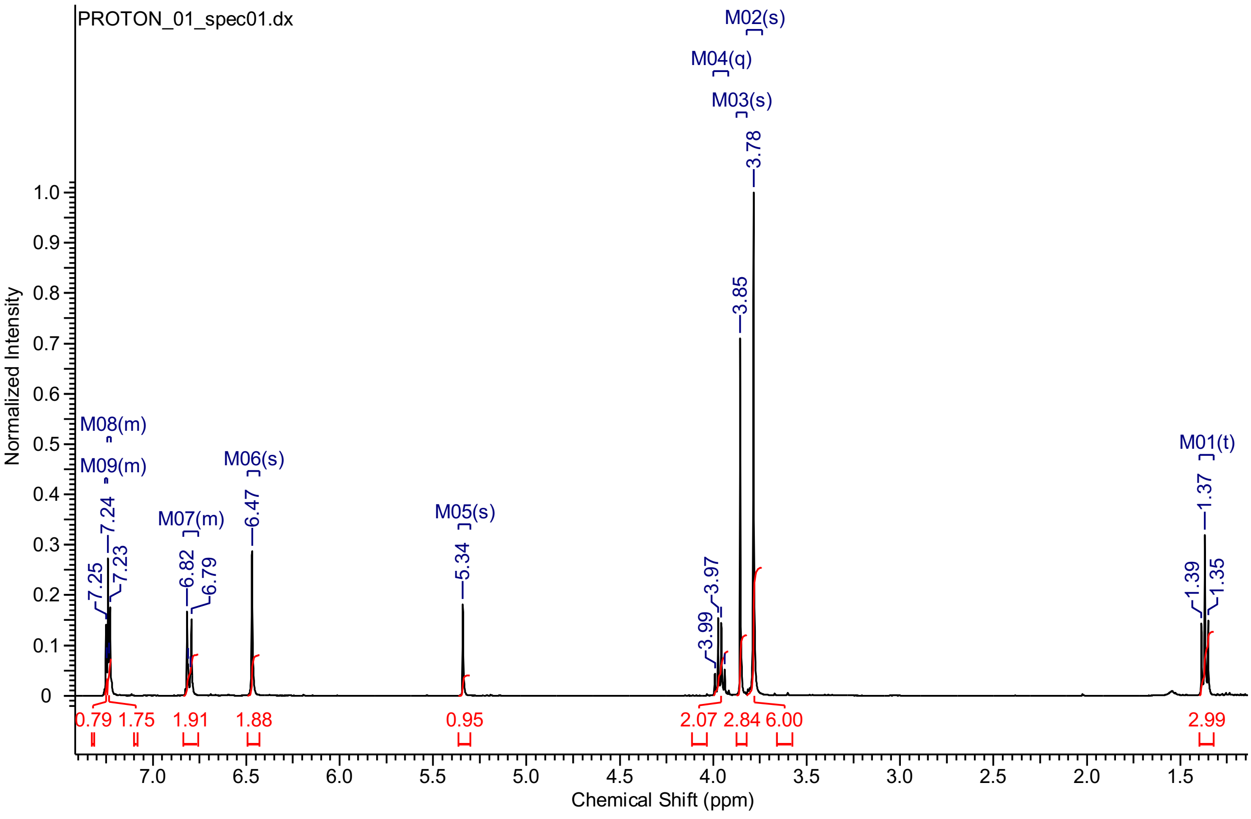

Figure S19: ^{1}H NMR (400 MHz, CHLOROFORM-*d*) spectrum of **(11f)**

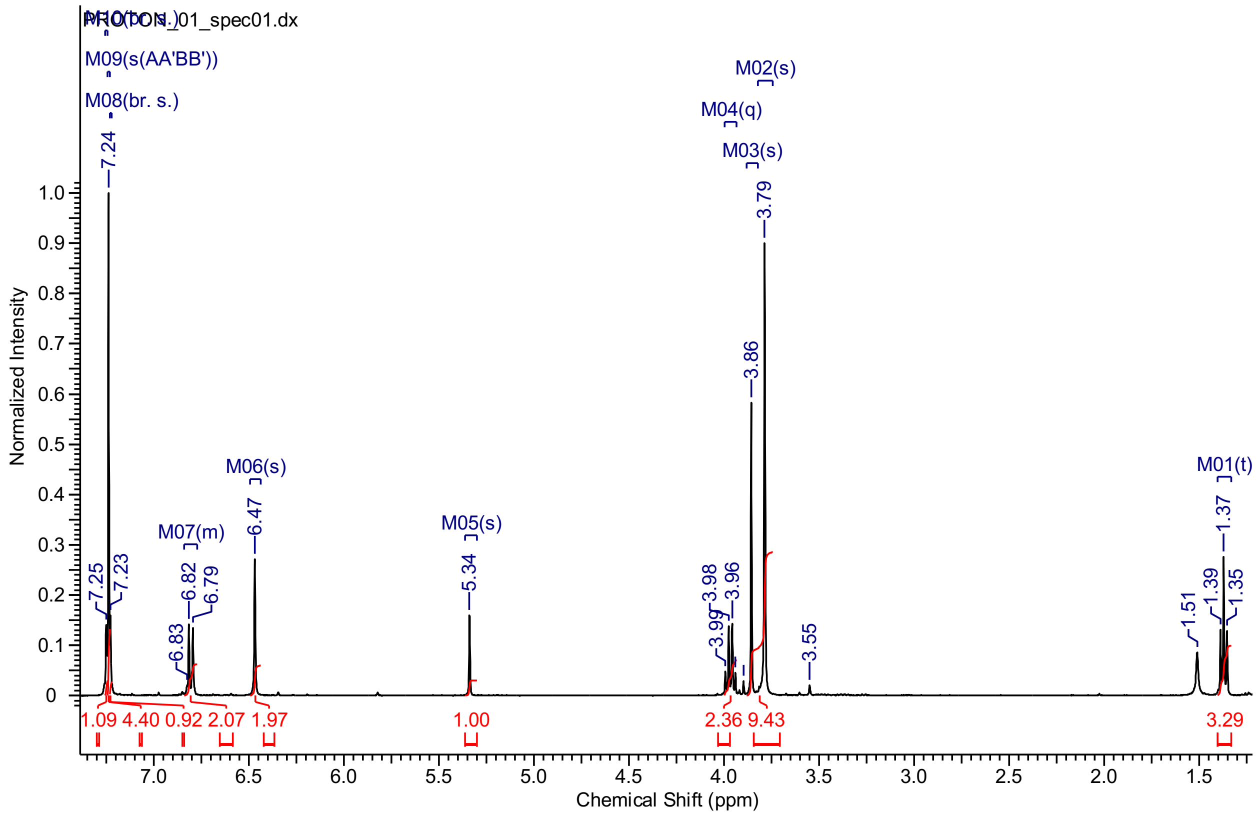

Figure S20: ^{1}H NMR (400 MHz, CHLOROFORM-*d*) spectrum of **(13b)**

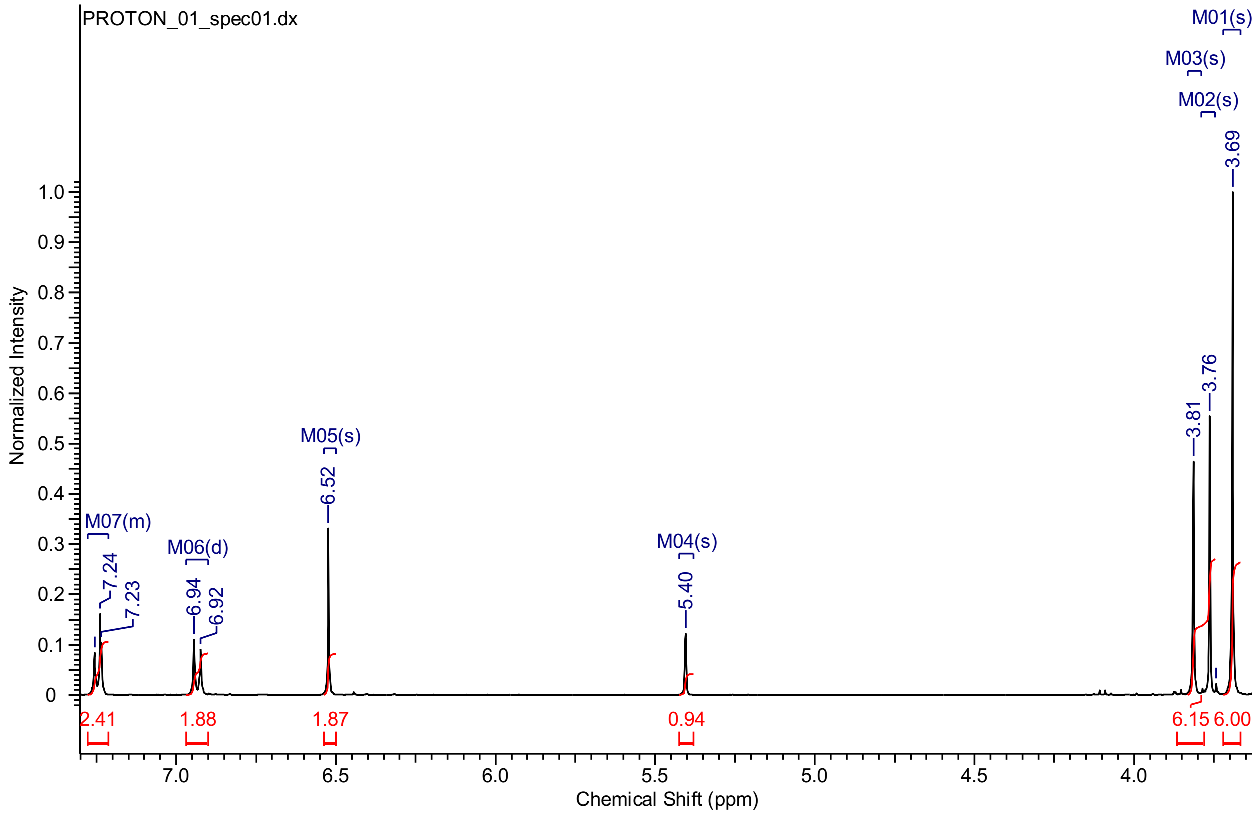

Figure S21: ^{1}H NMR (400 MHz, CHLOROFORM-*d*) spectrum of **(11e)**

References

1. Malebari, A.M., et al., *beta-Lactam analogues of combretastatin A-4 prevent metabolic inactivation by glucuronidation in chemoresistant HT-29 colon cancer cells.* Eur J Med Chem, 2017. **130**: p. 261-285.
2. Malebari, A.M., et al., *beta-Lactams with antiproliferative and antiapoptotic activity in breast and chemoresistant colon cancer cells.* Eur J Med Chem, 2020. **189**: p. 112050.
